# snoRNA and piRNA expression levels modified by tobacco use in women with lung adenocarcinoma

**DOI:** 10.1371/journal.pone.0183410

**Published:** 2017-08-17

**Authors:** Natasha Andressa Nogueira Jorge, Gabriel Wajnberg, Carlos Gil Ferreira, Benilton de Sa Carvalho, Fabio Passetti

**Affiliations:** 1 Laboratory of Functional Genomics and Bioinformatics, Oswaldo Cruz Institute, Fundação Oswaldo Cruz, Rio de Janeiro, RJ, Brazil; 2 D’or Institute for Reserach and Education, Rio de Janeiro, RJ, Brazil; 3 Department of Statistics, State University of Campinas, Campinas, SP, Brazil; Institut de Pharmacologie Moleculaire et Cellulaire, FRANCE

## Abstract

Lung cancer is one of the most frequent types of cancer worldwide. Most patients are diagnosed at advanced stage and thus have poor prognosis. Smoking is a risk factor for lung cancer, however most smokers do not develop lung cancer while 20% of women with lung adenocarcinoma are non-smokers. Therefore, it is possible that these two groups present differences besides the smoking status, including differences in their gene expression signature. The altered expression patterns of non-coding RNAs in complex diseases make them potential biomarkers for diagnosis and treatment. We analyzed data from differentially and constitutively expressed PIWI-interacting RNAs and small nucleolar RNAs from publicly available small RNA high-throughput sequencing data in search of an expression pattern of non-coding RNA that could differentiate these two groups. Here, we report two sets of differentially expressed small non-coding RNAs identified in normal and tumoral tissues of women with lung adenocarcinoma, that discriminate between smokers and non-smokers. Our findings may offer new insights on metabolic alterations caused by tobacco and may be used for early diagnosis of lung cancer.

## Introduction

Lung cancer is one of the leading causes of death from cancer in both men and women worldwide [1]. The most common type of lung cancer is non-small-cell lung cancer (NSCLC), accounting for 85% of the cases [2]. Lung cancer is often detected when the disease is at an advanced clinical stage and thus has a poor prognosis [3–5] and with a high mortality rate.

Smoking is one of the risk factors for this disease [6]. There are many well-known carcinogens in tobacco that bind to DNA and create somatic mutations such as the ones observed in the KRAS gene in lung cancer and in TP53 in several cancer types [7]. However, most smokers do not develop lung cancer while 20% of women with lung adenocarcinoma are non-smokers [8]. Therefore, other factors than smoking status may contribute to the development of lung cancer [9].

On the other hand, cigarette smoke is known to change gene expression in the transcriptome of MSK-Leuk1 cell line [6], epithelial cells [10] and buccal mucosa [11] and has been reported to alter biological pathways related to signal transduction, asthma and cell proliferation [6,12,13]. Thus, many studies have searched for differences in gene expression that may reveal changes in the genetic profile between smokers and non-smokers [6, 12] but none have evaluated the expression patterns of small nucleolar RNA (snoRNA) or PIWI-interacting RNA (piRNA).

piRNA ranges from 26 to 31 nucleotides long and is one of the least investigated classes of sncRNAs, and many aspects of its biogenesis and mechanism are still unknown. The major role of piRNA is to silence transposable elements [14,15] in germline cells (reviewed by [12, 13]), thus acting in stem cell division, apoptosis, epigenetic control of transposons and telomeres, and translational control [16]. However, piRNAs are also expressed in somatic tissues [17–19], where they act as gene expression regulators by inducing histone modifications and DNA methylation [20,21]. Importantly, these molecules are altered in several types of cancer [22,19,23,24]. Thus, a better understanding of the expression pattern of these molecules could contribute to lung cancer biology, early detection and survival.

On the other hand, snoRNAs are an abundant sncRNA class that comprises 60 to 300 nucleotides long sncRNA molecules, which associate with the nucleolar enzymes methylase and pseudouridine synthase to form the small ribonucleoprotein responsible for rRNA methylation and pseudouridylation, respectively [25]. However, some snoRNAs do not have an identified target rRNA. Different groups have been reporting 20 to 24 nucleotides long small RNAs derived from the further processing of snoRNAs. These new RNAs seems to act as miRNAs [26] suggesting other functions for these molecules [27]. Several snoRNAs have been found to be altered under hypoxia [28] and oxidative stress conditions [29]. Additionally, SNORA42 is amplified and up-regulated in NSCLC and its levels inversely correlate with survival in NSCLC [30].

One of the most common approaches to study sncRNA is to produce a large-scale profile with techniques like microarray, then validate the findings using strategies such as RT-qPCR [31]. However, these approaches require the use of probes or primers, which means that some sequence fragments must be known *a priori*, making the identification of truly novel genes a complicated task. Recently, the high-throughput sequencing (HTS) technology was used to study sncRNA. The technology is highly precise [32] and sensitive, allowing for the inference at gene expression level, detection of chromosome rearrangements, mutations, novel transcripts, isoforms, and low expressed genes besides the identification of novel genes [33,34]. For instance, Müller and collaborators [24] used high-throughput sequencing technology to evaluate the coding and non-coding transcriptome of six pancreatic cancer samples. Besides several deregulated miRNAs, the authors found that the snoRNAs HBII-296B and U104 as well as piRNA piR-017061 were differentially expressed in tumors when compared to normal cells. However, the role of piRNAs and snoRNAs in cancer is still unknown and warrants further investigation.

There are many commercially available HTS platforms, each with its specifications, such as throughput, read size, error frequency, cost, and the number of sequenced reads [32]. The vast amount of data produced can be stored in public databases and made available to the global scientific community. Different datasets can be combined, after quality control, normalization, and evaluation of the information regarding each experiment, to improve the detection of weaker signals and generate new knowledge, without the burden of one single research center generating all data. Rung and Brazma [35] performed a survey on how often public data from ArrayExpress were mentioned in published papers in 2011 and found that almost one in four papers used data that were available in that public database to answer new biological questions different from the question raised in the original study that collected the data. For instance, Kröger and colleagues (2016) used several public microarray experiments to identify new altered genetic pathways in blood mononucleolar cells of systematic lupus erythematosus patients [36], while Gonzalez-Porta and colleagues (2012) used public RNA-Seq data from HapMap to evaluate different splicing patterns in Caucasians and Yorubas [37]. Also, Cao and colleagues (2015) [11] used public microarray data to identify more than 300 genes differentially expressed between smokers and non-smokers.

In this article we detected, for the first time, differentially and constitutively expressed piRNAs and snoRNAs in smoker versus non-smoker women with lung adenocarcinoma. Our data were collected from publicly available datasets from samples of patients and may offer new insights in sncRNA biology and on the effects of tobacco use on molecules and cancer biology.

## Material and methods

We obtained two datasets of small RNA-sequencing from lung adenocarcinoma samples belonging to female patients from The Cancer Genome Atlas [38] (TCGA) and from the work published by Kim and collaborators [39]. It is important to stress that throughout this study we use the term ‘normal tissue’ to describe and discuss data obtained from the normal tissue adjacent to tumors.

Kim and collaborators [39] used the Illumina Genome analyzer IIx to sequence the small RNAs (ranging from 22 to 30 nucleotides long) present in six matched primary lung adenocarcinoma tumors and normal tissues from never-smoker women [NCBI GEO:GSE37764]. Although the authors only assessed the expression of miRNAs, they mention the possibility of the raw data containing sncRNAs other than microRNAs.

The 36-nucleotide length reads from the data published by Kim and collaborators were investigated for quality and adaptor presence. The adaptors were removed with Cutadapt [40] and only the reads longer than 15 nucleotides were kept. Also, any read with more than 10% of its bases with quality lower than 20 was removed using the software FASTQ Quality Filter from FASTX-Toolkit (Gordon and Hannon, unpublished). The remaining reads were aligned to the human genome using Novoalign (version 3.02.13, http://www.novocraft.com) returning reads that aligned at a single location in the human reference genome.

The TCGA project is a collaboration between the National Cancer Institute (NCI) and the National Human Genome Research Institute (NHGRI) that generated and made publicly available genomic, transcriptomic and methylomic data from 33 types of cancer. We downloaded (January 2016) paired normal tissue and tumor miRNA-Seq data from samples belonging to the same patient, obtaining a total of 6 paired samples from 6 women (dbgap data access committee Project ID 43224–3).

TCGA data were generated using either the Illumina Genome Analyzer or the Illumina HiSeq. The files downloaded in the BAM file format were converted to FASTQ using BEDtools [41]. Converted files were aligned to the human genome using Novoalign with the same configuration as described above, aiming for the detection of sncRNAs other than miRNA.

The piRNA annotation file was obtained from the piRNAbank database [42], and overlapping annotations that were in the same orientation were grouped into clusters (S1 File). This modified GTF file has 2,049 piRNA clusters and 11,710 piRNAs without any overlap. The annotation file for the snoRNA was obtained from UCSC [43]. The piRNA and snoRNA annotation files were merged into a single file (S2 File). Any piRNA regions that overlapped with snoRNA annotations were considered as snoRNA, and the ones that overlapped with miRNAs annotations were discarded as annotation errors.

The raw counts for each annotated piRNA and snoRNA present in the samples sequenced using the same technology were obtained using BEDtools [41] (S3 File).The identification of the differentially expressed piRNAs and snoRNAs was performed using R version 3.4.0 and following the Bioconductor package EdgeR version 3.18.1 manual [44]. In short, we used the TMM methodology [45] to normalize the count per million (CPM) values obtained from the raw counts, and the negative binomial test to identify the differentially expressed genes. In this step, only small RNAs with at least 50 reads mapped in at least half of the samples, and those with counts greater than 0 in all but two samples were considered. We called differentially expressed all snoRNAs and piRNAs with FDR lower than 0.01 and logarithm of fold change greater than 2 or lower than -2. Aiming to validate the efficacy of our strategy, we first used it to investigate the differentially expressed miRNAs in Kim and collaborators and TCGA data using an annotation file obtained from MiRBase version 20 [46].

According to Eisenberg and Levanon, housekeeping genes are constitutively expressed in all cell types under normal conditions [47]. Therefore, to identify only the snoRNAs and piRNAs altered by tobacco usage and not by tumor status, we performed a data dispersion analysis on the samples from non-smokers and smokers according to the procedures described in [47]. Briefly, we kept only the sncRNAs with more than 1 CPM in all samples, whose expression did not differ more than 2 times from the average log2 normalized counts, and those with standard deviation lower than 1. These sncRNAs were considered putative housekeeping genes.

## Results

### Methodology validation

Kim et al. [39] pointed out 40 differentially expressed miRNAs in their work. Using our approach, we obtained 23 miRNAs that were differentially expressed between normal tissue and tumor samples belonging to non-smokers. Twenty of these were similar to those found by the original authors. Four other miRNAs found by Kim et al. [39] were also found by us but removed either by the false discovery rate (FDR) or logarithm of fold change filter (S4 File).

The same approach described above was adopted between normal tissue and tumor samples using smokers’ data. We found 23 differentially expressed miRNAs (S5 File). Only 5 miRNAs were found in both analyses. Out of those, 2 were found up-regulated in tumors, hsa-miR-183-5p, hsa-miR-210-3p, while 3 were down-regulated in tumors: hsa-miR-144-5p, hsa-miR-451a, and hsa-miR-30c-2-3p. The comparison between normal tissue samples from non-smokers and smokers revealed very distinct expression profiles with 130 differentially expressed miRNAs (S6 File). Although this distinction was not as clear when tumor samples were compared, we still found 135 miRNAs that were differentially expressed (S7 File).

### Comparison between lung tissues of smokers and non-smokers

**Fig 1:**
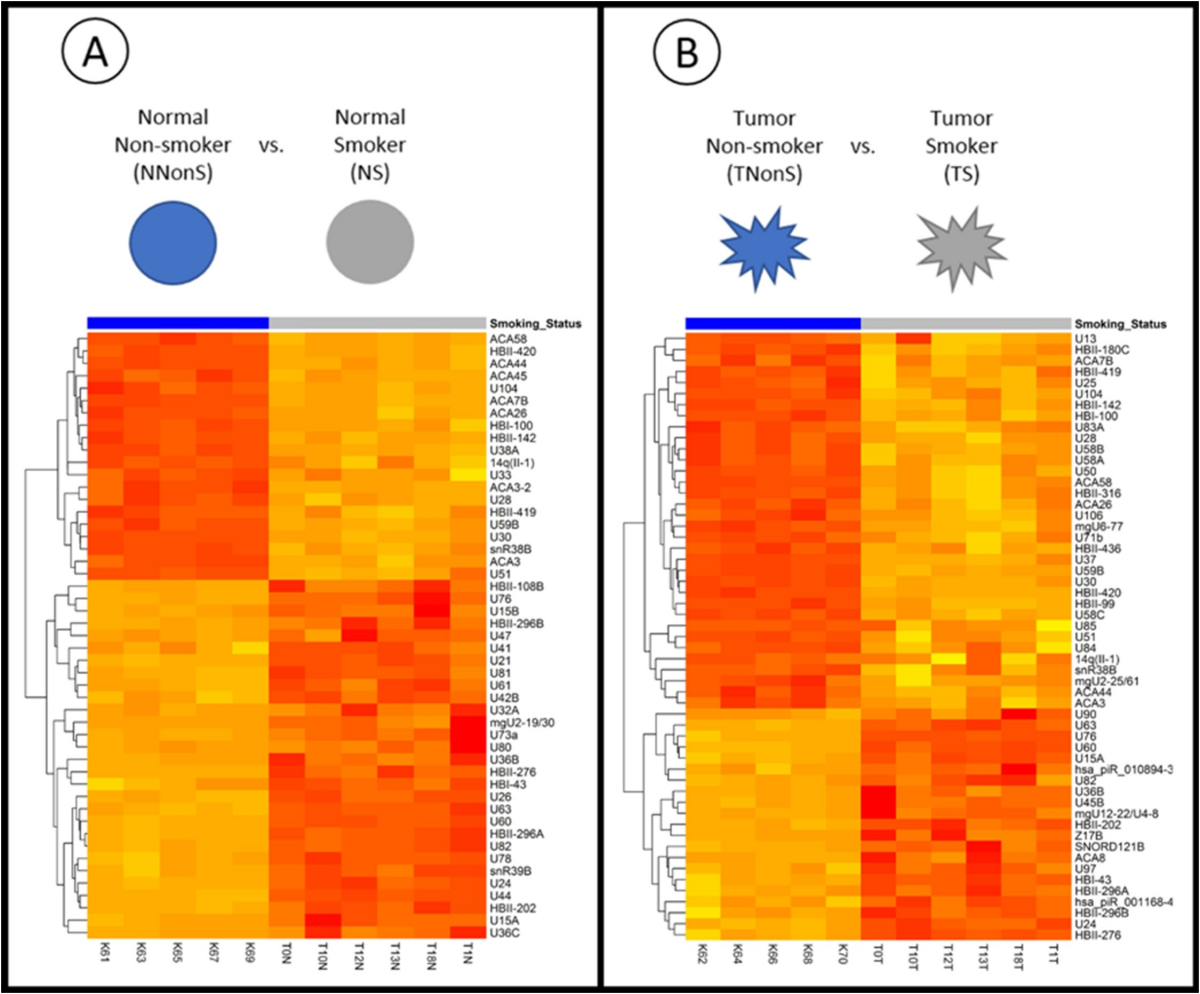
Differential expression comparisons performed in this study and heatmaps. A) Non-smoker normal versus Smoker normal samples. B) Non-smoker tumor versus Smoker tumor samples. Heatmap of Log_2_ of Normalized CPM counts for differentially expressed genes. Data below the blue bar are from non-smokers, and those below the gray bar are from smokers. The figure shows four sets of genes whose expressions are very different according to the smoking status.

Aiming to detect differences in the expression levels of piRNA and snoRNA between lung samples belonging to smoker and non-smoker women, we performed two differential expression analysis involving four groups of samples: normal non-smoker (NNonS) versus normal smoker (NS) samples (Fig 1A), and tumor non-smoker (TNonS) versus tumor smoker (TS) samples (Fig 1B).

First, we analyzed the differentially expressed genes in normal tissue samples from non-smokers as compared to smokers. After applying our FDR and logFC criteria, we detected 49 differentially expressed snoRNA between NNonS and NS samples (S8 File). According to our analysis, 29 snoRNAs are more expressed in NNonS and 20 are down-regulated (Fig 1A). U60 (SNORD60) is the most up-regulated snoRNA (logFC = 6.22) and HBII-420 (SNORD99) is the most down-regulated snoRNA (logFC = -5.25). The magnitudes of fold-change are similar between the up- and–down regulated genes. Table 1 shows the logFC and logCPM of the 10 greatest changes found in this analysis. No piRNA was found differentially expressed between NNoS and NS.

**Table 1:** Top 10 differentially expressed snoRNAs between normal tissue samples from non-smokers and smokers.

Gene	logFC	logCPM	FDR
U60	6.22	18.78	2.31E-57
U76	5.84	11.88	6.63E-30
U15A	5.50	11.34	5.55E-24
U44	4.23	12.19	6.63E-30
HBII-296A	3.82	11.00	9.94E-21
HBII-142	-3.44	15.29	4.53E-22
snR38B	-3.85	15.37	1.52E-21
ACA7B	-4.21	9.70	1.21E-20
U30	-5.23	16.87	7.16E-36
HBII-420	-5.25	13.92	2.01E-30

logFC: logarithm of Fold Change; logCPM: logarithm of CPM; FDR: false discovery rate; Positive logFC indicates genes more expressed in normal tissue samples from non-smokers.

The differential expression analysis of the Non-smokers tumor (TNonS) and Smokers tumor (TS) samples allowed the identification of 55 piRNA or snoRNA that presented altered levels of expression according to smoking status (S9 File and Fig 1B). In this analysis, 34 genes were found up-regulated in TNonS and 21 down-regulated. Again, U60 is the most up-regulated gene (logFC = 5.63), while U30 is the most down-regulated gene (logFC = -6.72). The magnitudes of fold changes are similar between the two groups as well. Table 2 shows the 10 greatest logFC changes. In this analysis, two piRNAs were found up-regulated in TNonS: has-piR-010894-3 (logFC = 3.43) and has-piR-001168-4 (logFC = 2.95).

**Table 2:** Top 10 differentially expressed snoRNA between tumor samples from smokers and non-smokers.

Gene	logFC	logCPM	FDR
U60	5.63	19.02	1.69E-28
U76	4.00	11.35	6.78E-22
U15A	3.89	10.75	2.34E-12
U63	3.01	13.33	2.67E-12
HBII-142	-3.00	13.91	2.34E-12
U58C	-3.60	9.94	5.95E-13
U37	-3.69	9.45	5.46E-13
HBII-99	-4.15	8.07	3.21E-15
HBII-420	-4.80	13.20	7.43E-30
U30	-6.72	17.81	5.38E-35

logFC: logarithm of Fold Change; logCPM: logarithm of CPM; FDR: false discovery rate; Positive logFC indicates genes more expressed in non-smoker tumor samples.

### Discriminative expression profile between groups

**Fig 2:**
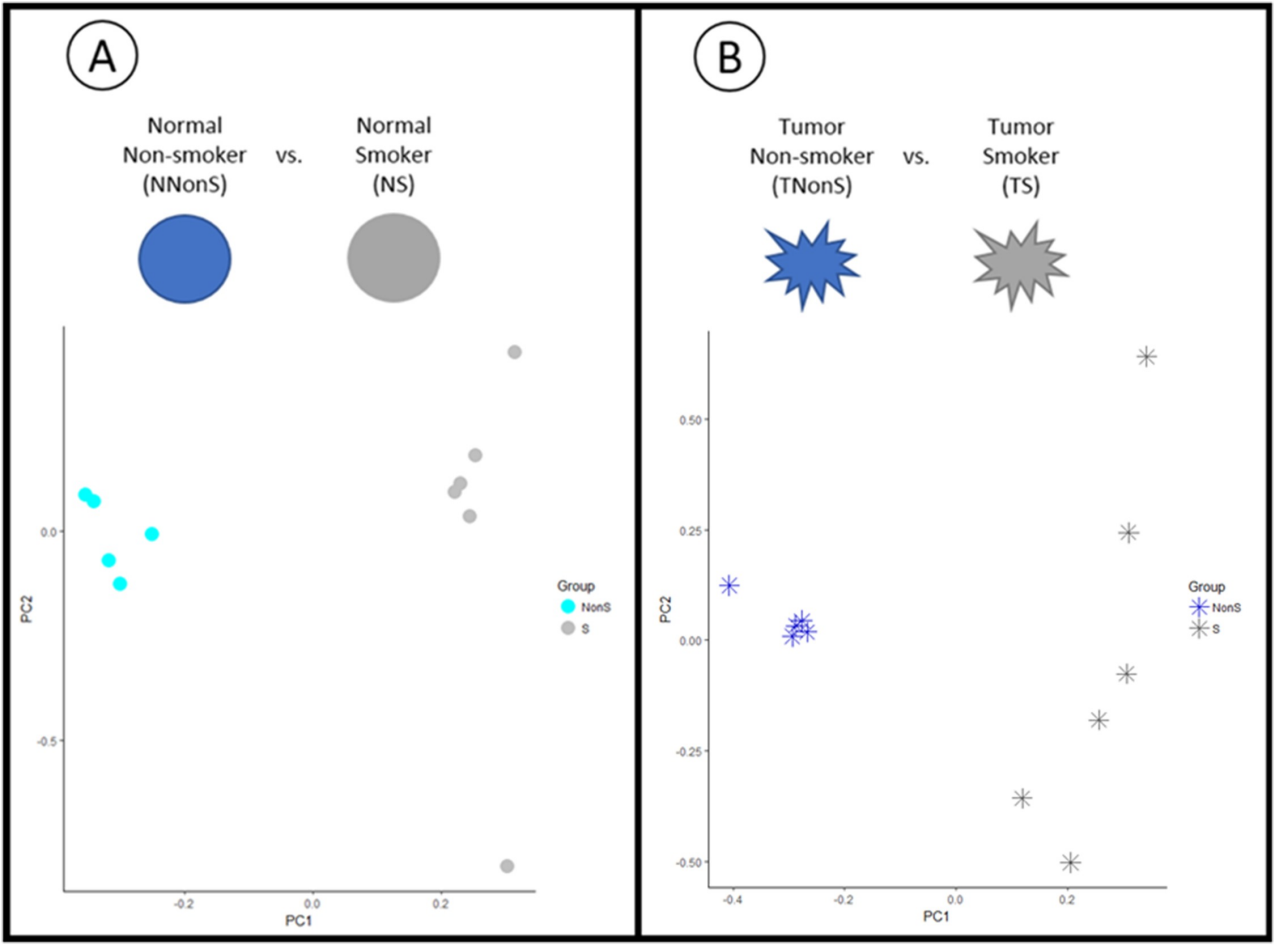
PCA analysis of the differentially expressed piRNAs/snoRNAs. A) Non-smoker normal (NNonS) versus Smoker normal (NS) samples. B) Non-smoker tumor (TNonS) versus Smoker tumor (TS) samples. The dots represent the normal samples, and stars the tumor samples. Light blue indicates Non-smokers normal samples, dark blue the Non-smokers tumor samples, light gray the Smokers normal samples and dark gray the Smokers tumor samples. Both analysis show distinct expression patterns between non-smokers and smokers.

Next, we sought to evaluate if the differentially expressed sncRNAs found in both comparisons can distinguish non-smokers from smokers by performing principal component analysis (PCA). This analysis showed distinct groups from non-smokers and smokers normal samples (Fig 2A) and tumor samples (Fig 2B). The greatest distinction was found in the comparison between non-smokers and smokers tumor, where the non-smoker samples showed a similar pattern while the smokers samples are dispersed.

According to our analysis, U60 (SNORD60) is up-regulated in the NNonS when compared to its expression in the NS and the same gene is also up-regulated in TNonS samples when compared to its expression in TS smokers. The same trend is found in U30 (SNORD30) that is down-regulated in NNonS when compared to its expression in NS and in TNonS when compared to its expression in TS. In fact, more than half of the differentially expressed snoRNAs were detected in both analyses (28 out of 49 normal samples and 28 out of 55 tumor samples) (Table 3). The complete list of differential expressed sncRNAs is provided as supplemental material (S8 and S9 Files). Additional comparisons between Normal and Tumor samples from Non-smokers and Normal and Tumor samples from Smokers are on S10 and S11 Files, respectively.

**Table 3:** snoRNAs differentially expressed in both normal tissue and tumor comparisons of non-smokers’ and smokers’ samples.

Gene	Normal		Tumor	
	logFC	logCPM	logFC	logCPM
**U60**	**6.22**	**18.78**	**5.63**	**19.02**
U76	5.84	11.88	4	11.35
U15A	5.5	11.34	3.89	10.75
HBII-296B	4.55	9.25	2.55	8.61
U36B	3.93	8.9	2.65	7.92
HBII-296A	3.82	11	2.68	10.41
HBII-202	3.72	10.82	2.22	9.74
U24	3.49	12.99	2.76	12.15
HBII-276	3.47	10.62	2.13	10.11
U82	3.1	12.44	2.05	11.71
**U63**	**2.63**	**13.61**	**3.01**	**13.33**
HBI-43	2.24	10.87	3.38	10.32
**U28**	**-2.06**	**10.96**	**-2.33**	**10.89**
**U51**	**-2.17**	**10.76**	**-3.03**	**10.96**
**U104**	**-2.21**	**16.35**	**-2.27**	**15.16**
**HBII-419**	**-2.27**	**13.59**	**-2.34**	**13.21**
**U59B**	**-2.66**	**10.53**	**-2.78**	**9.88**
ACA26	-3.04	7.84	-4.23	9.84
ACA3	-3.33	7.33	-2.2	7.47
**HBII-142**	**-3.44**	**15.29**	**-3**	**13.91**
ACA58	-3.75	9.25	-4.66	9.44
snR38B	-3.85	15.37	-2.05	13.25
**HBI-100**	**-3.89**	**10.9**	**-3.18**	**9.98**
ACA44	-3.96	8.77	-2.81	8.15
ACA7B	-4.21	9.7	-2.17	8.92
14q(II-1)	-5.01	11.04	-3.57	9.52
**U30**	**-5.23**	**16.87**	**-6.72**	**17.81**
**HBII-420**	**-5.25**	**13.92**	**-4.8**	**13.2**

logFC: logarithm of Fold Change; logCPM: logarithm of CPM; Positive logFC indicates genes up-regulated in normal tissue samples if compared to tumor samples. A total of 28 genes were found to be differentially expressed in both analysis. All genes found presented the same expression pattern (up- or down-regulated) regardless of the pathological status. The snoRNAs in bold face type showed low dispersion in samples from non-smokers and smokers.

**Fig 3:**
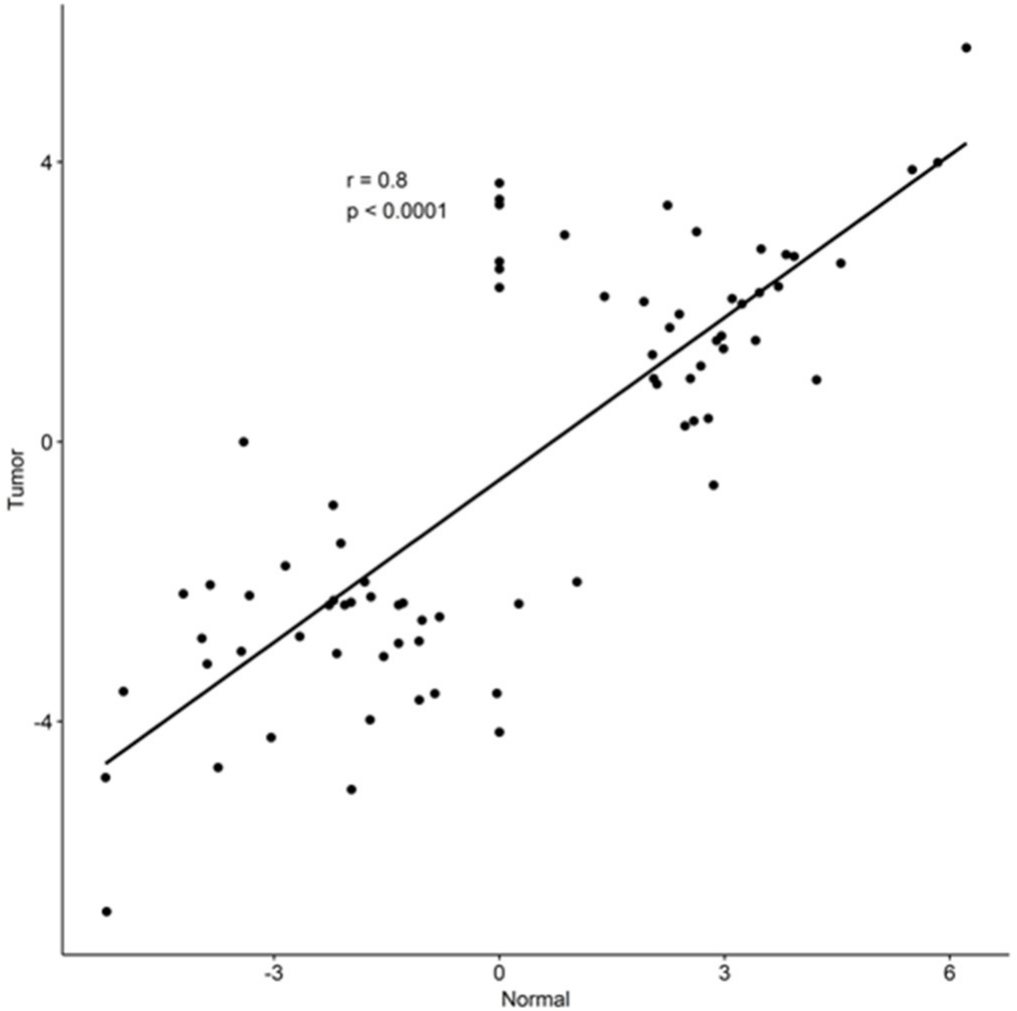
Scatterplot of the log2 fold change for the 28 snoRNAs shared between analysis.

**Fig 4:**
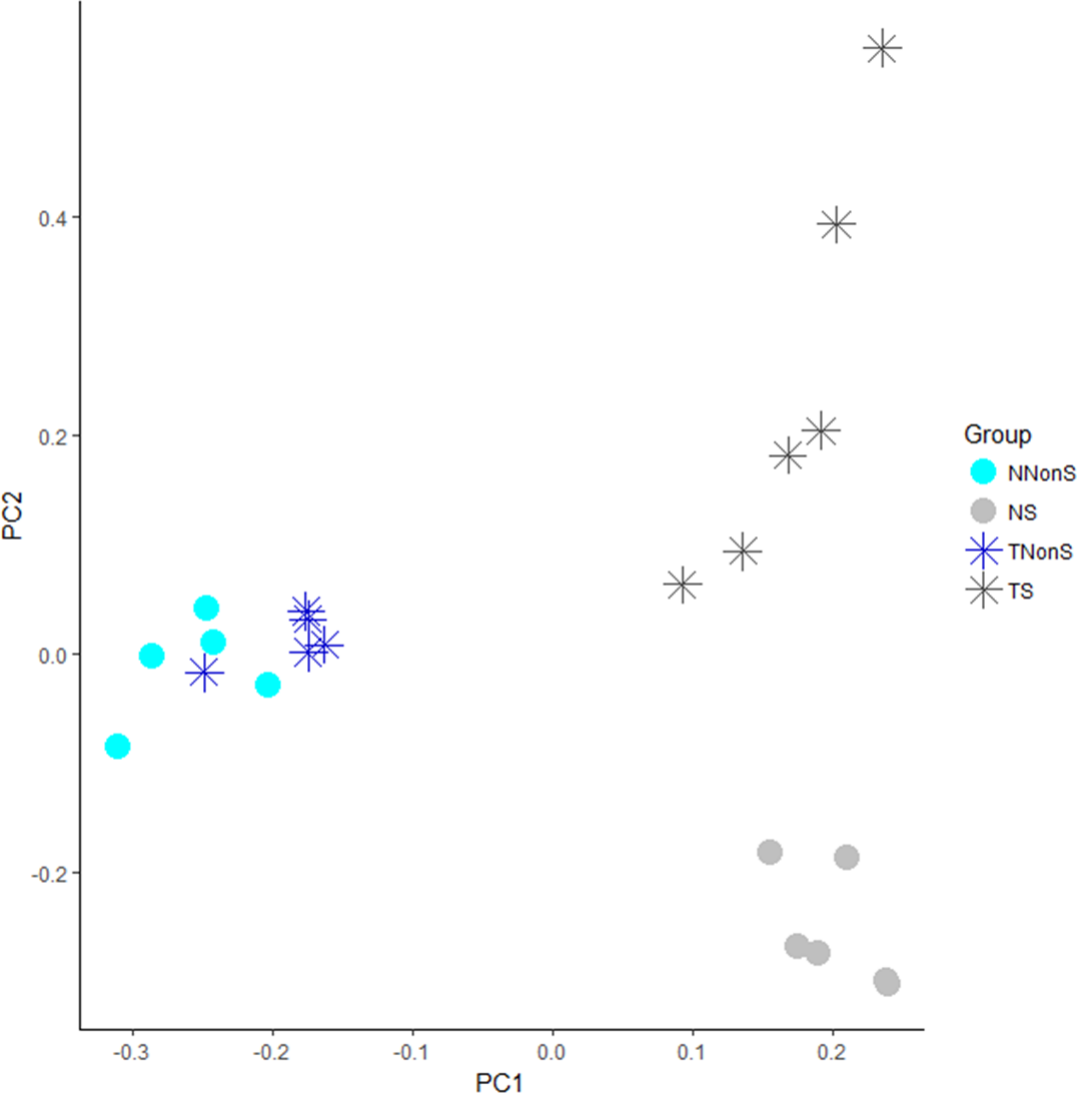
PCA analysis of the differentially expressed piRNAs/snoRNAs. The dots represent the normal samples, and the stars the tumor samples. Light blue indicates Non-smokers normal samples, dark blue the Non-smokers tumor samples, light gray the Smokers normal samples and dark gray the Smokers tumor samples. This PCA shows 3 distinct groups that correspond to Smokers normal samples, Smokers tumor samples and Non-smokers samples.

Based on this result, we investigated if the expression pattern of these snoRNAs presented the same trend, regardless of the sample pathological status. In this analysis, only snoRNAs and piRNAs that were differentially expressed in at least one set of samples were considered. According to our results, there was not a single sncRNA that showed opposite expression between the Normal and Tumor analysis (Pearson coefficient = 0.80) (Fig 3). The principal component analysis with all differentially expressed piRNAs or snoRNAs also shows clearly distinct expression differences between non-smokers and smokers (Fig 4). The normal and tumor samples from smokers also show distinct patterns, while the non-smokers samples present the same trend.

### sncRNA expression dispersion analysis between non-smokers and smokers

We investigated patterns of data dispersion for samples belonging to non-smokers and smokers, regardless of their pathological status. We performed two data dispersion analysis: NNosS and TNonS and NS and TS.

After applying our constitutive expression criteria, we found 179 snoRNA or piRNAs to be constitutively expressed in samples from non-smokers (S12 File) and 33 genes identified as constitutive in samples from smokers (S13 File). The log2 of the normalized CPM of each sample, and the variance and the standard deviation of the 10 snoRNAs that presented the lowest standard deviation in non-smokers and smokers are shown in the Tables 4 and 5, respectfully. All constitutive snoRNAs found in the smokers were also found in the non-smokers samples. However, the lowest variations and standard deviations were found in the non-smokers samples. The only exception is U25, whose expression pattern is more uniform for smokers (Table 6). A total of 11 snoRNAs found to be constitutively expressed in both analyses had expression levels changed in the non-smokers versus smokers comparisons: 2 are up-regulated in non-smokers and 9 are up-regulated in smokers (Boldface type in Table 3).

**Table 4:** Top 10 constitutively expressed snoRNA in samples from non-smokers.

Gene	K61	K62	K63	K64	K65	K66	K67	K68	K69	K70	Var	SD
U51	8.45	8.48	8.54	8.58	8.79	8.37	8.57	8.76	8.53	8.35	0.02	0.15
U57	12.20	12.38	12.34	12.30	12.07	11.92	12.37	12.36	12.25	11.67	0.05	0.23
HBII-429	9.42	9.37	9.57	9.40	9.81	9.60	9.34	8.92	9.72	9.42	0.06	0.25
HBII-95	5.89	5.62	5.37	5.94	5.87	5.94	5.77	6.12	5.38	5.63	0.06	0.25
U21	13.34	13.27	14.15	13.56	13.60	13.52	13.54	13.68	13.77	13.30	0.07	0.26
U35B	4.98	4.85	4.84	4.14	4.83	4.45	4.93	4.82	4.96	4.74	0.07	0.26
U37	6.65	6.37	5.98	5.95	6.25	6.13	6.21	6.73	6.32	6.67	0.08	0.28
U75	9.08	9.38	8.77	9.23	8.66	8.88	9.01	8.94	8.90	8.42	0.08	0.28
U95	12.08	12.11	12.30	11.66	12.48	11.85	12.26	11.87	12.36	12.45	0.08	0.28
ACA8	5.96	5.27	4.95	5.11	5.50	5.64	5.45	5.52	5.36	5.68	0.08	0.29

SD: standard deviation; Var: variance. Columns 2 to 11 represent each of the non-smokers samples analyzed in this study. Samples ending in odd numbers are from the normal tissue adjacent to tumor and those ending in even numbers represent tumor samples. The numbers bellow each sample corresponds to log2 of the normalized CPM counts. Overall, the non-smokers samples presented the lowest expression variation.

**Table 5:** Top 10 constitutively expressed snoRNA in samples from smokers.

Gene	T0N	T0T	T10N	T10T	T12N	T12T	T13N	T13T	T18N	T18T	T1N	T1T	Var	SD
U59B	11.50	11.05	11.56	11.58	11.53	11.03	11.90	11.49	11.74	10.87	11.08	11.23	0.10	0.32
U25	13.24	13.95	12.68	12.93	12.97	12.98	13.14	13.57	13.13	13.52	12.62	12.57	0.17	0.41
U57	12.71	12.29	12.37	12.40	12.28	11.78	12.25	12.91	12.67	11.72	11.69	11.88	0.17	0.41
U48	12.81	13.26	13.23	13.56	13.49	13.46	13.34	12.75	13.38	14.38	13.39	12.89	0.19	0.43
U27	14.04	14.30	13.27	14.65	14.07	13.93	14.05	14.04	13.93	13.40	12.96	13.92	0.21	0.46
U21	11.90	11.95	12.52	11.94	12.28	12.87	11.28	12.62	12.36	12.85	13.21	12.03	0.29	0.53
HBII-420	14.95	14.53	15.03	14.01	14.24	14.78	14.67	14.85	14.49	14.90	16.25	14.67	0.30	0.55
U20	13.15	13.07	13.00	11.70	12.94	12.96	12.70	13.61	12.90	11.75	12.98	12.22	0.33	0.57
U43	13.73	12.91	12.84	12.45	13.74	13.25	13.43	12.72	14.26	13.67	14.02	12.59	0.36	0.60
snR39B	12.67	12.55	12.00	11.33	12.42	13.40	12.90	12.97	12.19	12.85	11.79	13.31	0.38	0.62

SD: standard deviation; Var; variance. Columns 2 to 13 represent each of the smokers samples analyzed in this Samples ending with “N” are from the normal tissue adjacent to tumor and those ending with “T” are from tumor samples. The variances and standard deviation for smokers were greater than the non-smokers, with exception of U25.

**Table 6:** snoRNAs found constitutively expressed in samples from both smoker and non-smoker patients.

Gene	Non-smoker			Smoker		
	Mean	Var	SD	Mean	Var	SD
U59B	7.78	0.09	0.30	11.38	0.10	0.32
U25	10.34	0.27	0.52	13.11	0.17	0.41
U57	12.19	0.05	0.23	12.25	0.17	0.41
U48	11.47	0.18	0.43	13.33	0.19	0.43
U27	14.34	0.14	0.37	13.88	0.21	0.46
U21	13.57	0.07	0.26	12.32	0.29	0.53
HBII-420	8.87	0.20	0.45	14.78	0.30	0.55
U20	10.69	0.11	0.33	12.75	0.33	0.57
U43	12.68	0.29	0.53	13.30	0.36	0.60
snR39B	13.19	0.23	0.48	12.53	0.38	0.62
U63	13.91	0.14	0.38	11.90	0.38	0.62
U104	13.66	0.37	0.61	16.78	0.42	0.65
HBII-336	11.03	0.44	0.66	11.49	0.43	0.66
HBII-419	11.27	0.23	0.48	14.34	0.44	0.66
SNORD119	12.97	0.29	0.53	12.55	0.45	0.67
U28	8.88	0.24	0.49	11.96	0.46	0.68
U42A	9.70	0.41	0.64	12.09	0.46	0.68
U38A	8.92	0.40	0.64	12.10	0.48	0.69
U59A	9.03	0.15	0.38	10.89	0.49	0.70
HBII-55	9.94	0.21	0.46	11.12	0.52	0.72
U31	13.95	0.17	0.42	15.06	0.53	0.73
HBII-142	11.65	0.25	0.50	15.74	0.55	0.74
ACA45	10.14	0.35	0.59	12.69	0.57	0.75
HBII-210	11.67	0.16	0.40	11.67	0.58	0.76
U52	11.03	0.34	0.58	11.64	0.60	0.78
U95	12.14	0.08	0.28	13.54	0.64	0.80
HBII-251	11.78	0.22	0.47	12.24	0.65	0.81
HBI-100	7.22	0.32	0.57	11.54	0.67	0.82
U60	19.47	0.53	0.73	14.43	0.71	0.84
U51	8.54	0.02	0.15	11.78	0.82	0.91
U50	8.63	0.17	0.41	11.53	0.88	0.94
HBII-295	11.43	0.29	0.54	13.22	0.90	0.95
U30	11.75	0.14	0.37	18.49	0.90	0.95

SD: standard deviation; Var: variance. The mean corresponds to the average of the log 2 normalized CPM expression values of each smoking status group. A total of 33 genes were found to be constitutively expressed in both non-smoker and smoker groups.

## Discussion

Small noncoding RNAs, such as snoRNAs and piRNAs, are involved in fundamental biological pathways, and have been considered as potential lung cancer biomarkers [25,30,48]. In this study, we compared the expression pattern of piRNAs, one of the least studied sncRNA classes, and snoRNAs, one of the most studied and well-known sncRNA classes, obtained from publicly available datasets from lung adenocarcinoma samples belonging to matched smoker and non-smoker women.

We obtained 5 matched samples from normal and tumor tissues belonging to non-smoker women from the work of Kim and colleagues [39]. Additionally, we also used TCGA data from 6 matched samples from normal tissue and lung adenocarcinoma belonging to smoker women. To validate our methodology, we first evaluated the profile of differentially expressed miRNAs and compared our results with the original publication. We found 20 miRNAs shared with the work of Kim and colleagues [39] while 4 other miRNAs found by the authors were removed by our FDR and logFC filters. For smokers from TCGA, 23 miRNAs were found differentially expressed. Several papers confirm our findings [49–53]. Principal component analysis and multidimensional analysis showed clear differences between the normal tissue and tumor samples from smokers and non-smokers (SFile 6 and 7).

In total, we identified 49 differentially expressed snoRNAs or piRNAs between NNonS and NS samples, 20 down-regulated and 29 up-regulated (Fig 1A). A total of 55 snoRNAs or piRNAs with altered expression were also identified between TNonS and TS samples, 34 down-regulated and 21 up-regulated (Fig 1B). The changes in expression profile are also confirmed by principal component analysis and multidimensional analysis (SFile 8 and 9). Twenty-eight snoRNAs were found in both analysis and showed the same expression pattern in both normal tissue and tumor samples (Fig 3), thus further indicating that this alteration in expression profile is related to smoking. One of these is the snoRNA U15A (SNORD15A), which is up-regulated in samples from non-smokers. Curiously, this snoRNA was reported as having an ‘miRNA-like’ function and being capable of silencing the reporter gene [54] as well as being related to the regulation of chromatin structure in fibroblasts [55]. SNORD15A may have other putative roles beyond the modification of ribosomal RNAs and our results suggest that it may be involved in a mechanism in which smoking could alter its cell expression profile.

Out of the 28 sncRNAs altered between non-smokers and smokers, the data dispersion analysis showed that 11 snoRNAs presented a constant expression across normal tissue and tumor samples that belong to individuals in the same smoking status group. Only two of them were up-regulated in the non-smoker samples: U60 (SNORD60) and U63 (SNORD63). U60 has been reported as an attenuator of pulmonary vasoconstriction in rats [56] and as a key factor in the regulation of plasma membrane cholesterol [57], while U63 (SNORD63) is located in a chromosomic region frequently deleted in myelodysplastic syndromes [58]. Thus, we hypothesize that smoking may inhibit the expression of snoRNAs that are important for cell maintenance.

Interestingly, many of the remaining 9 snoRNAs that were found up-regulated in smokers were reported as altered in different cancer types. One example is the HBII-142 (SNORD66), located on a chromosome region frequently amplified in tumors [25]. SNORD66 was already suggested as a biomarker candidate for lung cancer due to its being up-regulated in the plasma of lung cancer patients [59]. For instance, U30 (SNORD30) was reported as up-regulated in pediatric gliomas [60] and seems to correlate with the shorter time to progression in multiple myeloma [61]. Another differentially expressed snoRNA was HBI-100 (SCARNA3), which showed to be up-regulated in breast cancer samples [62]. In peripheral T-cell lymphoma, U59B (SNORD59B) correlated with long-term survival [63]. Taken together, our findings suggest that tobacco use modifies the expression profile of several snoRNAs towards a pattern like that observed in the malignant phenotype.

Many snoRNAs still do not have an identified target and their expression patterns are still unknown. In the work of Lan and collaborators [64], authors assess the expression of HBII-420 (SNORD99) and ACA44 (SNORA44). Both genes are located in introns of the SNHG12 long non-coding RNA, and knocking down the host expression does not alter the snoRNAs expression [64]. Further studies on snoRNAs targets may reveal new biological pathways affected by smoking. These findings can be used to better understand different tobacco-related pathologies and improve their treatment.

We found that U44 was up-regulated in samples from non-smokers and constitutively expressed in samples from non-smokers. Interestingly, this is an snoRNA frequently used for normalization in qRT-PCR experiments [65]. According to our findings, the use of this snoRNA may create bias between non-smokers and smokers. Similar alterations in the expression of this snoRNA were already reported in colorectal and breast cancer [65,66].

Of the 33 snoRNAs found to be constitutively expressed, 16 were not significantly different between smokers and non-smokers, suggesting that they are not modified by tobacco use. Among those, U43 (SNORD43) is frequently used as normalization parameter for miRNA expression profile experiments due to its stable levels, thus reinforcing our findings [67]. More studies on the expression levels of these snoRNAs may reveal other candidates for normalization parameters.

As for piRNAs, it is known that PIWI proteins and piRNAs protect integrity and stability of the genome by regulating transposable elements. However, PIWI proteins have been described to be differently expressed in NSCLC and to be associated with patient survival [68–70]. It is speculated that aberrant transposable elements could increase the number of deletions, rearrangements, and duplications that are frequently observed in the genome of cancer cells [71].

Although snoRNAs and piRNAs are receiving more attention from research groups, there are still few reports assessing changes in their expression under different biological conditions and, until the submission of this paper, we could not find any report that has evaluated the expression patterns of this many snoRNAs and piRNAs in smokers’ and non-smokers’ samples.

To our knowledge, in many lung studies searching for genes playing a role in the disease, data between non-smoker and smoker samples were not separated [30,48–50,53]. However, here we show that these two groups have distinct gene expression profiles. This is line with data generated elsewhere [12,13]. Therefore, data from these two groups of individuals should be considered separately in future studies to avoid the introduction or errors and confusing factors that may lead to misleading results.

Our results show that smoking modifies the expression of many sncRNAs, thus changing their expression profile towards one that is more like to that reported in different types of cancer. Here we report distinct sets of sncRNAs that can be used to distinguish smokers and non-smokers and should be considered when analyzing data from these two groups. Further studies about sncRNA targets should reveal new affected biological pathways.

## Conclusions

The identification of a molecular signature in lung cancer that permit the discrimination of tumors from smokers and non-smokers is still a challenge. In this article, we report several snoRNAs and piRNAs that are differentially expressed in lung adenocarcinoma samples from smoker and from non-smoker women. We believe that these sets of constitutively and differentially expressed snoRNAs and piRNAs can be used in the future to improve the molecular diagnosis and treatment of lung cancer patients. Our findings highlight the importance of studying sncRNAs in cancer biology and their application as potential biomarkers in the era of precision medicine.

## Supporting information

S1 File Overlapping piRNA clusters. (TXT)

S2 File snoRNA piRNA annotation file in BED format. (BED)

S3 File Raw snoRNA and piRNA counts. (TSV)

S4 File Non-smoker Normal vs Tumor miRNA analysis. (PDF)

S5 File Smoker Normal vs Tumor miRNA analysis. (PDF)

S6 File Normal Non-smoker vs Smoker miRNA analysis. (PDF)

S7 File Tumor Non-smoker vs Smoker miRNA analysis. (PDF)

S8 File Normal Non-smoker vs Smoker snoRNA piRNA analysis. (PDF)

S9 File Tumor Non-smoker vs Smoker snoRNA piRNA analysis. (PDF)

S10 File Non-smoker Normal vs Tumor snoRNA piRNA analysis. (PDF)

S11 File Smoker Normal vs Tumor snoRNA piRNA analysis (PDF)

S12 File Non-smoker variance analysis. (PDF)

S13 File Smoker variance analysis. (PDF)
