# Supplementary material for: snoRNA and piRNA expression levels modified by tobacco use in women with lung adenocarcinoma
Source: PLoS One. 2017 Aug 17;12(8):e0183410. doi: 10.1371/journal.pone.0183410 (PMC5560661; doi:10.1371/journal.pone.0183410)
Supplement: S4 File — (PDF) [file pone.0183410.s004.pdf]

Supplemental File 4

miRNA analysis

Normal Non-Smoker x Tumor Non-Smoker

for the manuscript: “snoRNA and piRNA expression levels modified by tobacco use in women with lung adenocarcinoma” by

Natasha Andressa Nogueira Jorge, Gabriel Wajnberg, Carlos Gil Ferreira, Benilton de Sa Carvalho, Fabio Passetti

We evaluated our methodology by comparing our results with those of Kim and collaborators. In our approach, the CPM counts were calculated using the EdgeR Bioconductor package and normalized using the TMM methodology. Figure 1 shows the total raw and normalized counts.

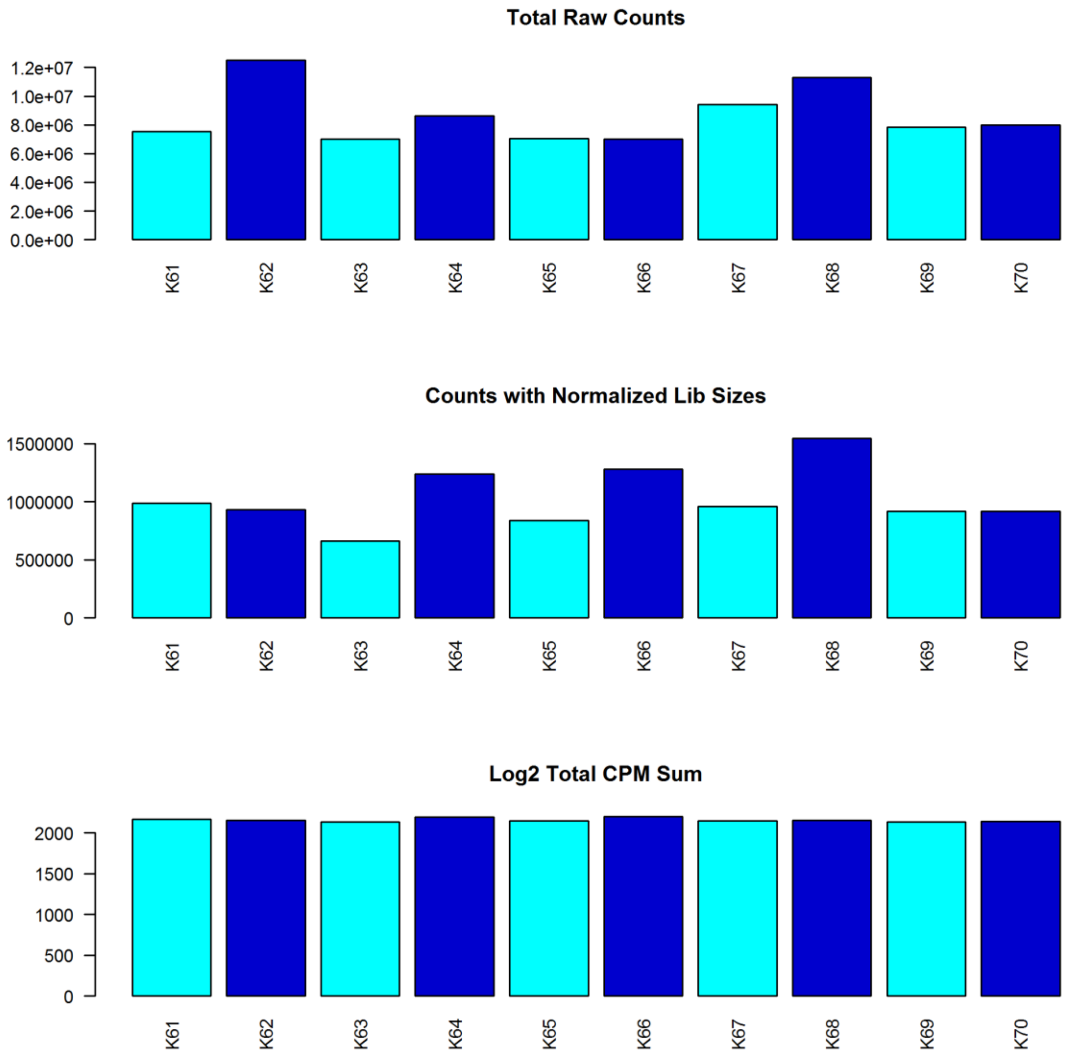

Figure 1. Raw, Normalized and log2 Normalized Total Counts. Light blue bars indicate Non-smokers normal samples and dark blue bars indicate non-smokers tumor samples.

Hierarchical clustering was performed on the normalized CPM counts (Figure 2). The miRNAs evaluated allowed the complete distinction of normal and tumor samples.

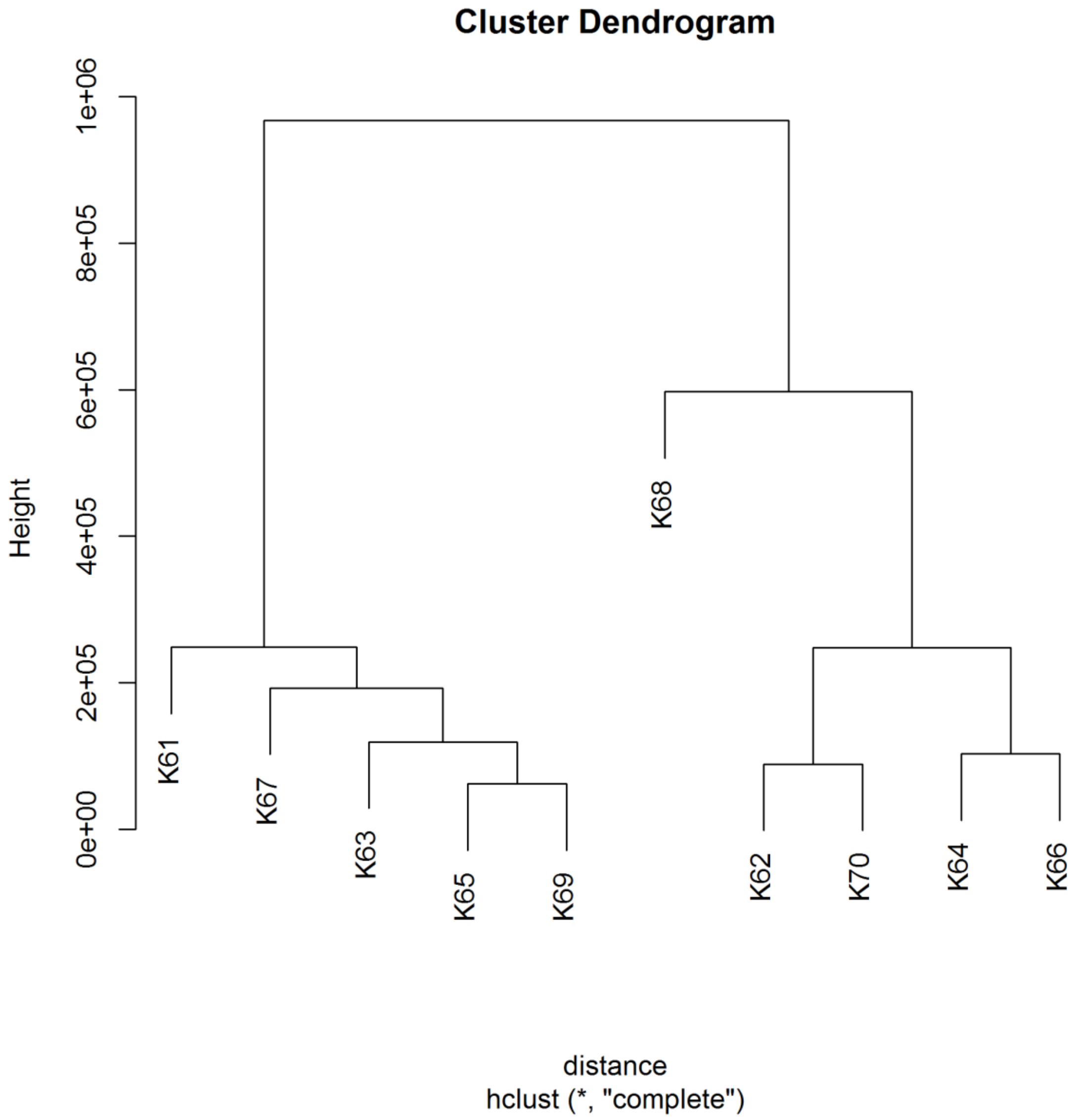

Figure 2. Hierarchical cluster for normalized counts. Samples ending with odd numbers correspond to normal samples and the ones ending in even numbers are tumor samples.

In order to further investigate the distribution of our samples, we used the normalized counts to perform principal component analysis. This analysis revealed two clearly distinct groups that correspond to the normal and tumor samples (Figure 3).

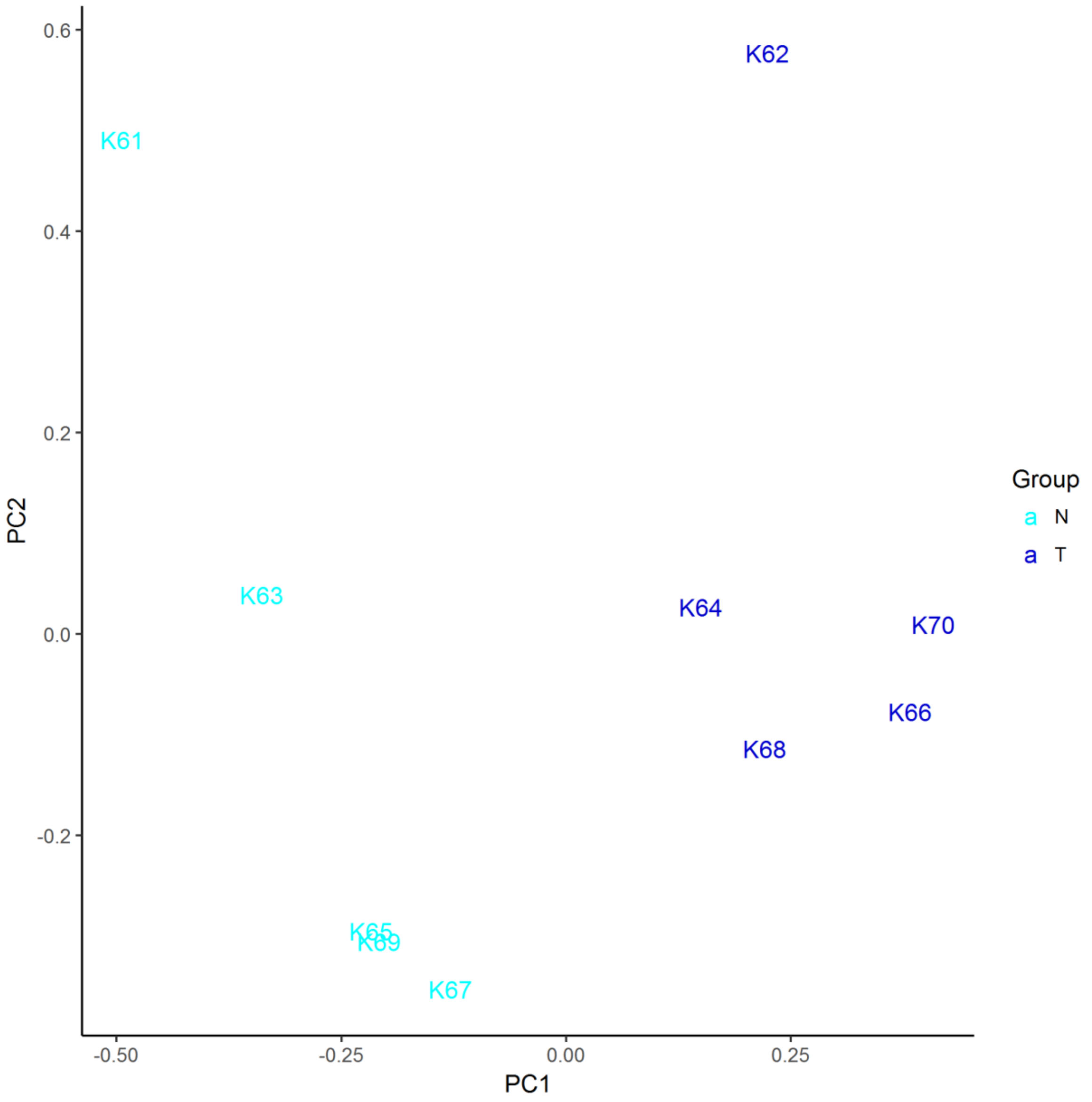

Figure 3. PCA analysis.

After applying our differential expression filters, FDR < 0.01 and logFC > 2 or logFC < -2, we found 23 differentially expressed miRNA (Figure 4). Most miRNAs, 13 miRNAs, are up-regulated in tumor samples, while 10 miRNAs are down-regulated (Figure 5). Out of the 23 miRNAs pointed out as differentially expressed by us, 20 were also found by Kim and collaborators (bold and italic type on Table 1). The miRNAs, hsa-miR-139-5p, hsa-miR-127-3p, hsa-miR-134-5p, and hsa-miR-889-3p were also considered as differentially expressed in the original work but did not pass our logFC threshold filter.

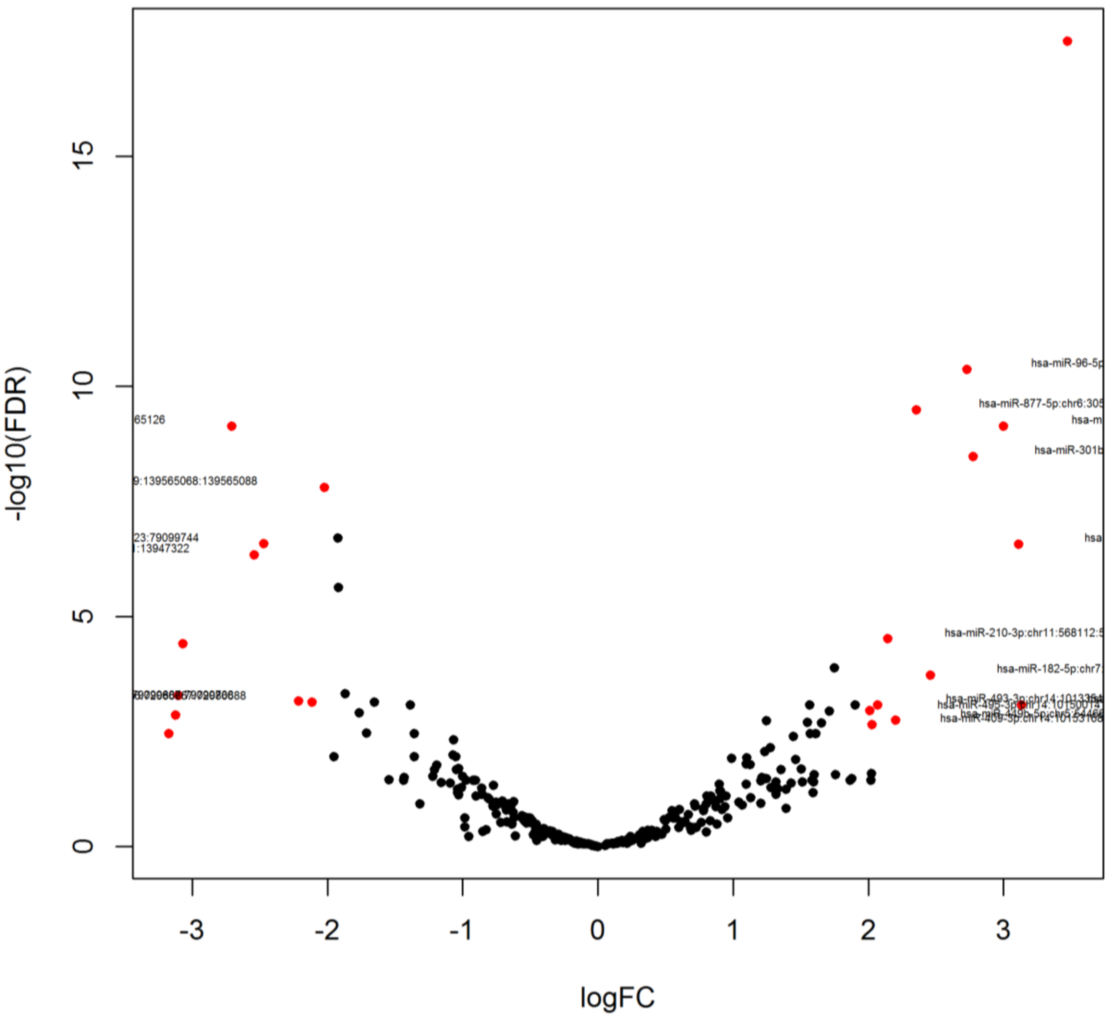

Figure 4. Volcano Plot. The red dots indicate the differentially expressed genes found.

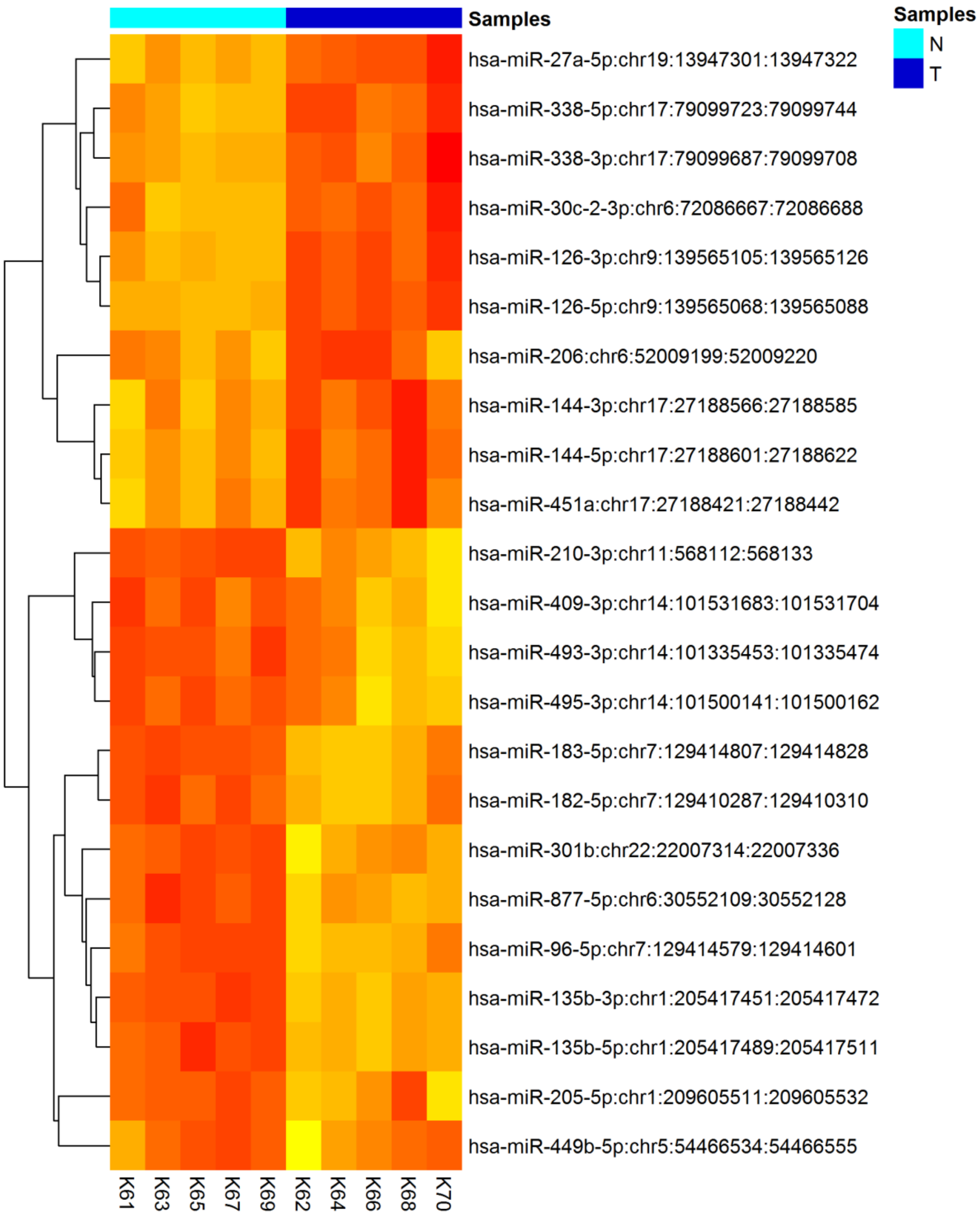

Figure 5. Heatmap. The samples ending in odd numbers refer to normal samples and the ones ending in even numbers to tumor samples. A total of 13 miRNAs were found up-regulated in tumor samples (yellow area on bottom right of the heatmap) and 10 up-regulated normal samples (yellow area on top right).

Table 1. Differentially expressed miRNAs.

Gene	K61	K62	K63	K64	K65	K66	K67	K68	K69	K70	logFC	logCPM	LR	PValue	FDR
hsa-miR-135b-3p:chr1:205417451:205417472	4.84	41.37	2.73	27.34	2.74	52.51	1.53	18.03	2.57	23.53	3.47	4.16	87.21	9.78E-21	3.14E-18
hsa-miR-96-5p:chr7:129414579:129414601	197.59	1274.12	97.29	730.89	80.30	740.43	68.28	571.31	73.45	244.18	2.73	8.67	53.45	2.65E-13	4.26E-11
hsa-miR-877-5p:chr6:30552109:30552128	4.44	24.14	1.41	9.79	2.38	11.67	3.97	15.43	2.34	12.17	2.36	3.17	48.68	3.02E-12	3.23E-10
hsa-miR-135b-5p:chr1:205417489:205417511	260.19	1310.66	153.04	879.40	63.64	1410.11	119.47	639.33	88.31	896.95	3.00	9.19	46.09	1.13E-11	7.27E-10
hsa-miR-126-3p:chr9:139565105:139565126	25439.42	6551.90	58642.82	9075.65	46170.14	6435.59	58875.07	12349.09	50410.05	3543.14	-2.71	14.76	46.08	1.13E-11	7.27E-10
hsa-miR-301b:chr22:22007314:22007336	7.45	105.03	5.83	24.61	2.38	14.59	3.56	14.07	2.57	27.09	2.77	4.39	42.76	6.19E-11	3.31E-09
hsa-miR-126-5p:chr9:139565068:139565088	6636.84	1781.59	7562.02	2253.55	7965.99	1653.70	9220.64	2591.32	7214.30	1383.25	-2.02	12.24	39.43	3.39E-10	1.56E-08
hsa-miR-338-5p:chr17:79099723:79099744	32.54	8.91	50.48	10.65	100.99	26.07	95.04	18.03	89.59	5.40	-2.47	5.46	33.43	7.39E-09	2.63E-07
hsa-miR-183-5p:chr7:129414807:129414828	347.09	4271.44	296.95	4861.25	395.77	4352.50	335.81	3239.93	465.74	820.61	3.11	10.92	33.19	8.36E-09	2.68E-07
hsa-miR-27a-5p:chr19:13947301:13947322	593.95	96.27	233.97	85.34	416.83	64.91	261.73	65.01	400.71	25.03	-2.54	7.81	31.99	1.55E-08	4.52E-07
hsa-miR-210-3p:chr11:568112:568133	84.68	445.83	112.90	188.08	84.34	307.95	82.32	421.62	76.61	756.89	2.14	8.00	23.53	1.23E-06	3.04E-05
hsa-miR-144-5p:chr17:27188601:27188622	23284.20	630.56	6188.65	3817.51	15482.68	2049.53	4105.19	304.57	14966.86	2315.90	-3.07	12.84	22.92	1.69E-06	3.87E-05
hsa-miR-182-5p:chr7:129410287:129410310	1933.05	11464.59	1141.28	16504.32	3053.40	18039.04	1632.84	11937.71	2916.43	3222.04	2.46	12.81	19.66	9.26E-06	0.000185756
hsa-miR-451a:chr17:27188421:27188442	180674.66	2745.89	33783.42	17156.93	102344.73	10952.37	16395.51	1086.62	71561.72	22251.29	-3.10	15.49	17.50	2.87E-05	0.00051169
hsa-miR-338-3p:chr17:79099687:79099708	215.89	90.62	321.58	71.09	514.97	206.21	424.34	99.70	436.27	17.79	-2.21	7.91	16.83	4.09E-05	0.000691753
hsa-miR-30c-2-3p:chr6:72086667:72086688	53.32	40.63	209.44	48.64	172.01	32.09	171.16	48.49	202.34	14.12	-2.11	6.64	16.56	4.72E-05	0.000721199
hsa-miR-493-3p:chr14:101335453:101335474	3.01	5.94	3.76	7.77	3.93	35.01	7.53	21.58	2.22	30.19	2.07	3.61	15.97	6.45E-05	0.000828206
hsa-miR-205-5p:chr1:209605511:209605532	113.17	1929.11	71.63	983.30	87.79	364.11	37.45	34.96	87.96	3283.00	3.13	9.45	15.89	6.71E-05	0.000828206
hsa-miR-495-3p:chr14:101500141:101500162	4.44	10.18	9.49	15.97	4.64	70.38	9.87	32.64	6.90	41.56	2.01	4.37	15.26	9.35E-05	0.00111151
hsa-miR-144-3p:chr17:27188566:27188585	554.22	13.59	50.95	45.47	319.28	15.86	62.07	4.37	151.00	51.32	-3.13	6.99	14.65	0.000129706	0.001387849
hsa-miR-449b-5p:chr5:54466534:54466555	65.73	843.82	5.92	33.39	2.85	17.69	1.63	6.97	5.38	4.48	2.20	6.63	14.13	0.000170744	0.001768024
hsa-miR-409-3p:chr14:101531683:101531704	2.22	6.17	5.73	9.64	2.62	26.62	8.65	17.21	3.74	49.36	2.03	3.74	13.48	0.000240509	0.002205807
hsa-miR-206:chr6:52009199:52009220	4.70	0.37	6.49	0.29	31.29	0.18	9.87	3.14	45.73	35.70	-3.17	3.81	12.46	0.000416666	0.003447559
