# Supplementary material for: snoRNA and piRNA expression levels modified by tobacco use in women with lung adenocarcinoma
Source: PLoS One. 2017 Aug 17;12(8):e0183410. doi: 10.1371/journal.pone.0183410 (PMC5560661; doi:10.1371/journal.pone.0183410)
Supplement: S5 File — (PDF) [file pone.0183410.s005.pdf]

Supplemental File 5

<u>miRNA analysis</u>

<u>Normal Smoker x Tumor Smoker</u>

for the manuscript: “snoRNA and piRNA expression levels modified by tobacco use in women with lung adenocarcinoma” by

Natasha Andressa Nogueira Jorge, Gabriel Wajnberg, Carlos Gil Ferreira, Benilton de Sa Carvalho, Fabio Passetti

We also performed the differential expression analysis between normal and tumor samples from smoker patients. The CPM counts were calculated using the EdgeR Bioconductor package and normalized using the TMM methodology. Figure 1 shows the total raw and normalized counts.

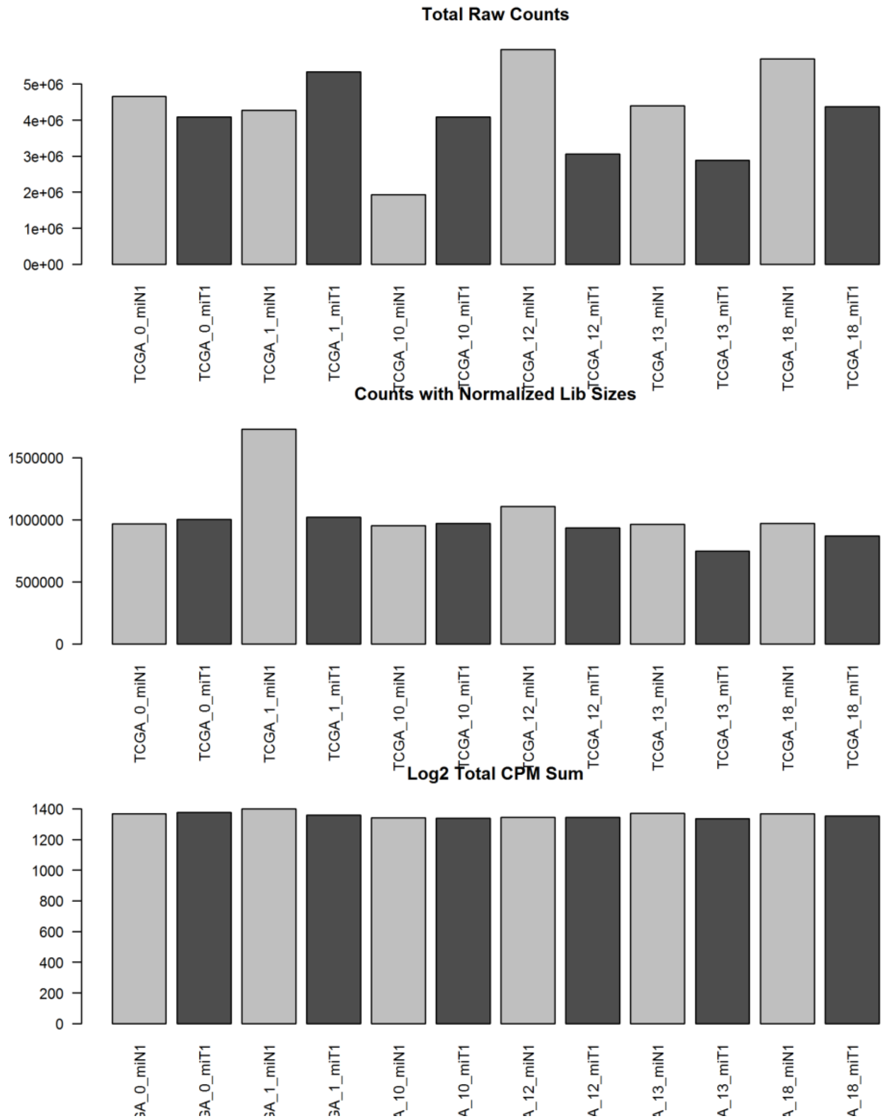

Figure 1. Raw, Normalized and log2 Normalized Total Counts. Light gray bars indicate normal samples and dark gray bars indicate tumor samples.

Hierarchical clustering was performed on the normalized CPM counts (Figure 2). The miRNAs evaluated allowed the complete distinction of normal and tumor samples.

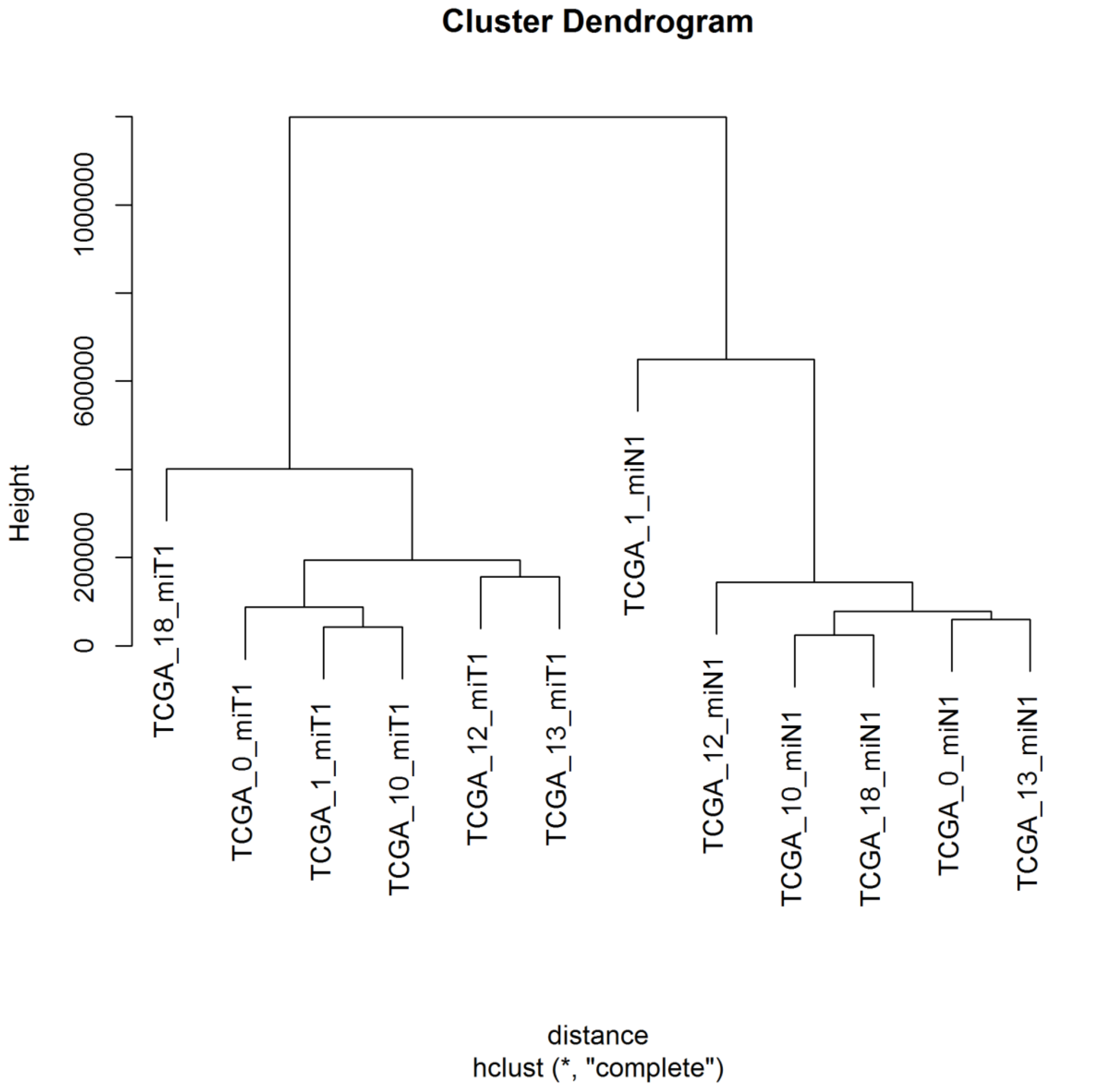

Figure 2. Hierarchical cluster for normalized counts. Samples ending in 'N1' correspond to normal samples and the ones ending in 'T1" are tumor samples.

To further investigate the distribution of our samples, we used the normalized counts to perform principal component analysis. This analysis revealed two clearly distinct groups that correspond to the normal and tumor samples (Figure 3).

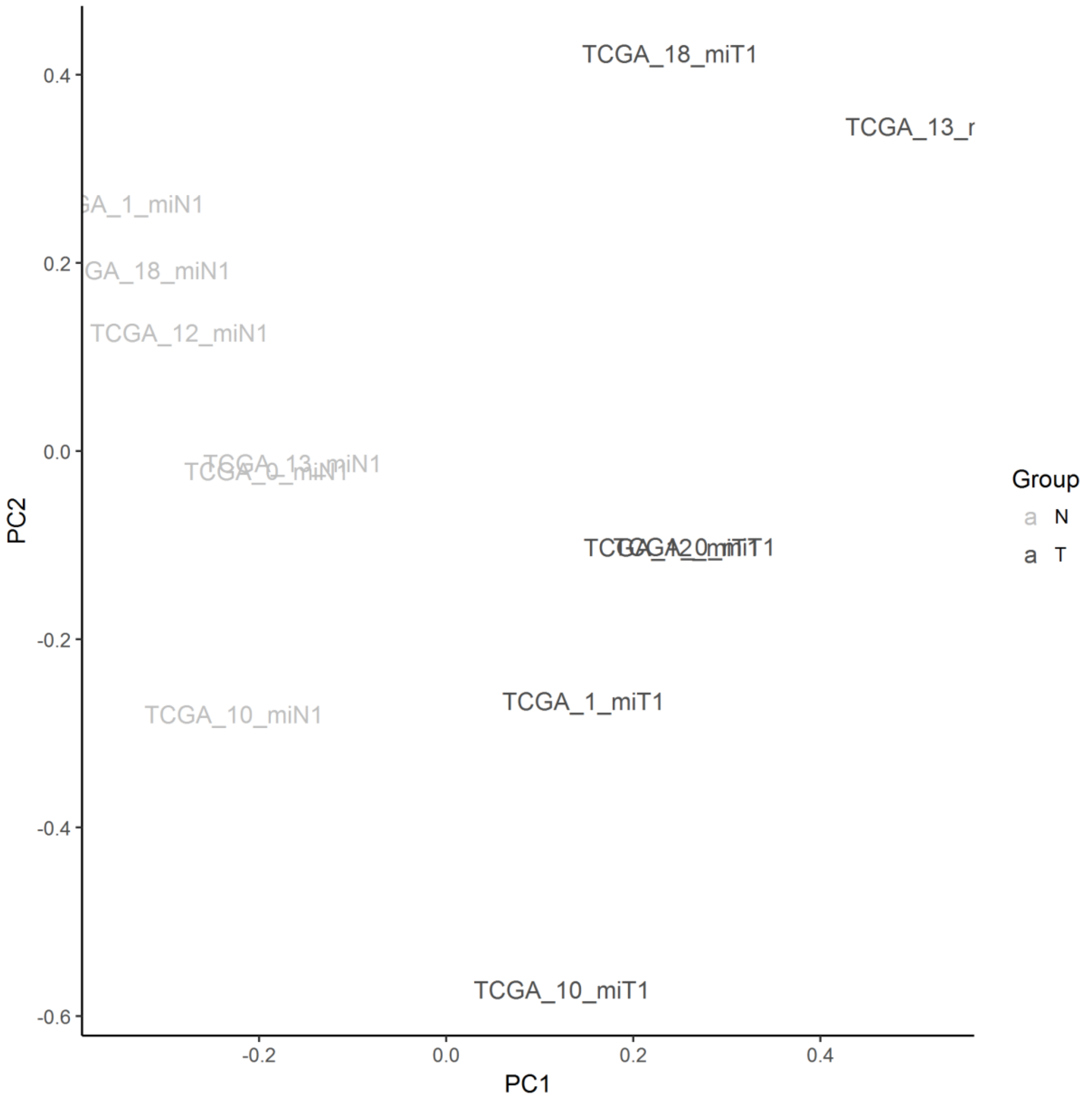

Figure 3. PCA analysis.

After applying our differential expression filters, FDR < 0.01 and logFC > 2 or logFC < -2, we found 23 differentially expressed miRNA (Figure 4). Most miRNAs, 14 miRNAs, are up-regulated in normal samples, while 9 miRNAs are down-regulated (Figure 5). Table 1 shows cpm, logFC, and FDR for each miRNA.

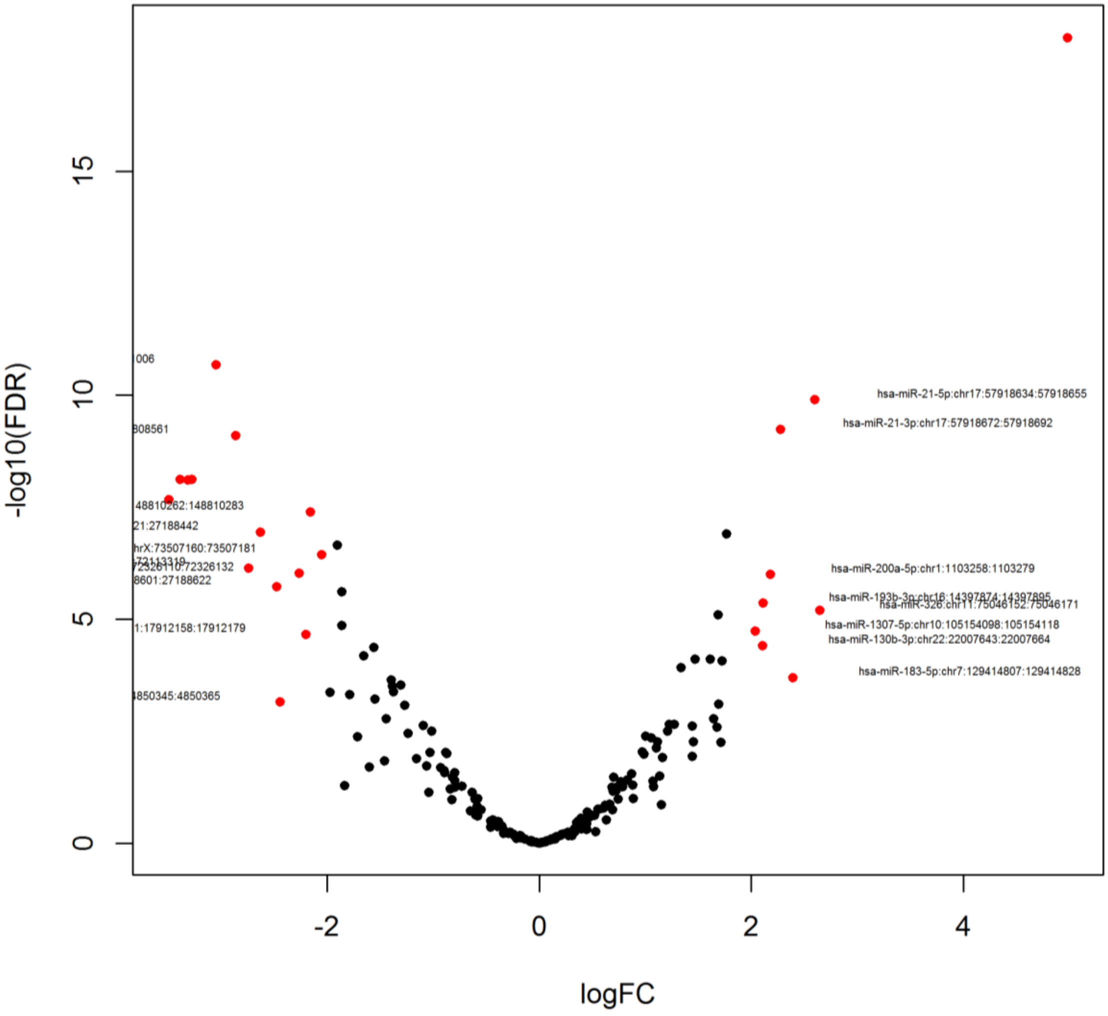

Figure 4. Volcano Plot. The red dots indicate the differentially expressed genes found.

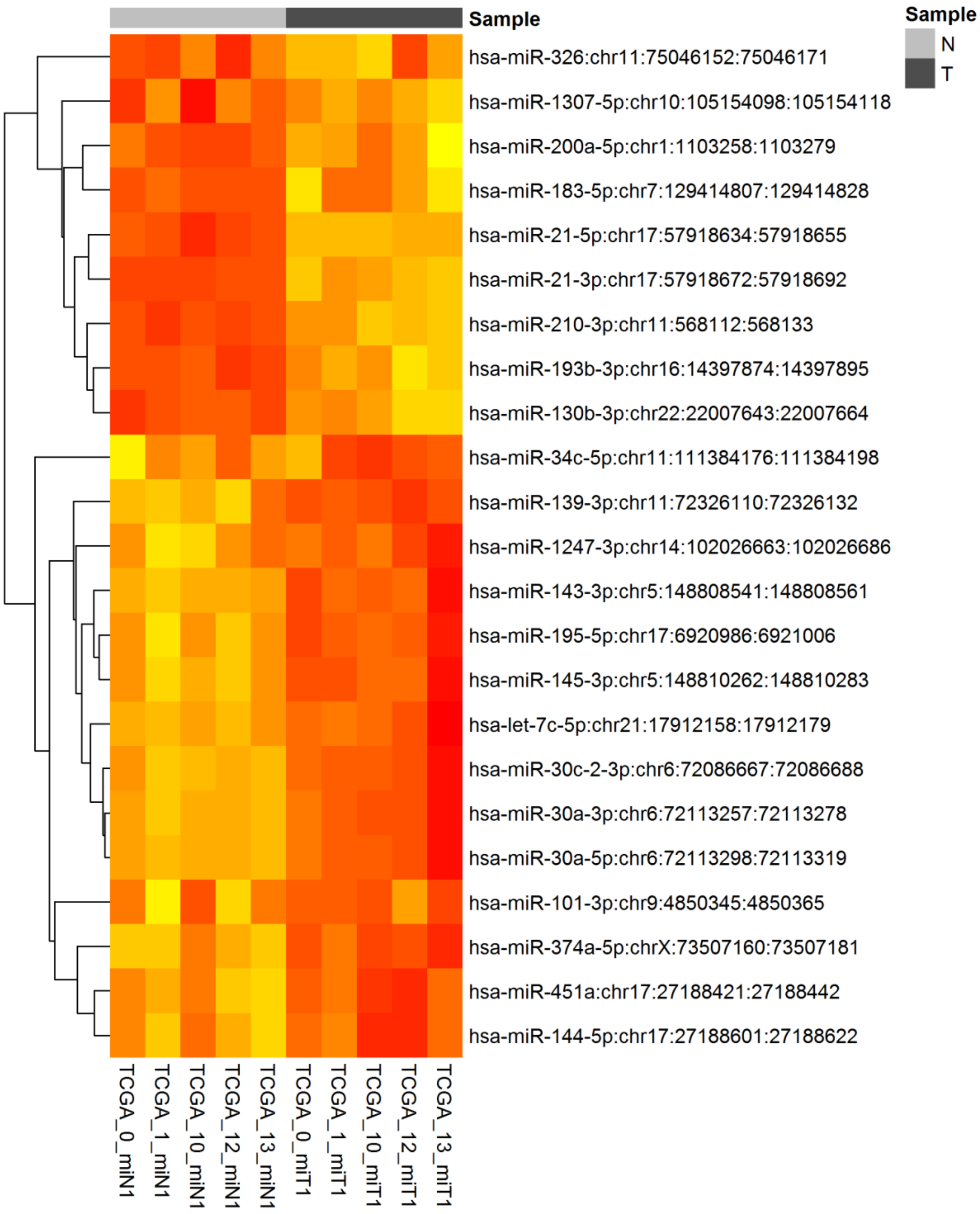

Figure 5. Heatmap. The samples ending with 'N1' refer to normal samples and the ones ending in 'T1' to tumor samples. A total of 14 miRNAs were found up-regulated in normal samples (yellow area on bottom left of the heatmap) and 10 up-regulated tumor samples (yellow area on top right).

Table 1. Differentially expressed miRNAs.

Gene	TCGA_0_miN1	TCGA_0_miT1	TCGA_1_miN1	TCGA_1_miT1	TCGA_10_miN1	TCGA_10_miT1	TCGA_12_miN1	TCGA_12_miT1	TCGA_13_miN1	TCGA_13_miT1	TCGA_18_miN1	TCGA_18_miT1	*logFC*	logCPM	LR	PValue	FDR
hsa-miR-210-3p:chr11:568112:568133	8.94	63.84	2.02	80.39	6.90	451.41	5.21	307.58	8.32	478.88	9.86	153.19	*4.98*	7.04	88.35	5.48E-21	1.04E-18
hsa-miR-195-5p:chr17:6920986:6921006	88.98	13.26	397.04	27.69	80.33	32.92	259.83	28.71	75.96	5.44	207.26	20.29	*-3.05*	6.69	53.80	2.22E-13	2.10E-11
hsa-miR-21-5p:chr17:57918634:57918655	101673.59	563039.07	85461.51	577258.27	43743.87	540305.58	68593.04	440678.34	92473.84	434603.89	53206.22	191158.67	*2.60*	18.02	49.49	2.00E-12	1.27E-10
hsa-miR-21-3p:chr17:57918672:57918692	523.49	4919.70	552.05	2149.51	631.33	2613.25	734.30	3483.54	753.05	4472.61	704.76	2140.10	*2.27*	10.95	45.97	1.20E-11	5.69E-10
hsa-miR-143-3p:chr5:148808541:148808561	560308.57	72465.98	1163621.74	136256.83	572616.99	121011.70	619243.41	159412.04	515854.13	20705.47	576374.23	105468.63	*-2.86*	18.56	44.90	2.07E-11	7.86E-10
hsa-miR-34c-5p:chr11:111384176:111384198	916.21	204.28	42.09	4.20	69.00	1.66	10.60	4.58	85.59	8.55	148.60	6.76	*-3.28*	6.98	40.00	2.53E-10	7.56E-09
hsa-miR-30c-2-3p:chr6:72086667:72086688	116.42	34.87	359.80	22.91	240.51	23.68	207.75	17.41	240.58	3.37	263.71	64.06	*-3.39*	7.06	39.82	2.78E-10	7.56E-09
hsa-miR-1247-3p:chr14:102026663:102026686	38.88	24.55	289.38	11.46	225.23	23.45	41.85	5.19	17.73	0.78	137.38	10.15	*-3.31*	6.11	39.53	3.22E-10	7.66E-09
hsa-miR-30a-3p:chr6:72113257:72113278	14742.05	5357.97	45269.02	2926.86	24833.44	1923.82	23045.32	2069.35	29122.88	344.98	25064.04	4946.89	*-3.49*	13.87	37.31	1.01E-09	2.13E-08
hsa-miR-145-3p:chr5:148810262:148810283	49.27	17.68	133.16	17.38	66.04	24.87	105.46	25.66	45.31	5.18	116.47	27.85	*-2.16*	5.74	35.86	2.12E-09	4.02E-08
hsa-miR-451a:chr17:27188421:27188442	892.09	386.71	1801.05	579.91	715.12	172.65	3242.98	161.27	4119.44	490.54	5562.40	474.89	*-2.63*	10.60	33.66	6.56E-09	1.13E-07
hsa-miR-374a-5p:chrX:73507160:73507181	94.39	21.85	103.21	35.71	34.01	15.87	67.70	21.38	101.79	12.69	105.59	13.93	*-2.05*	5.72	30.92	2.69E-08	3.64E-07
hsa-miR-30a-5p:chr6:72113298:72113319	28182.25	12756.06	58869.55	8883.31	48241.07	6912.54	38761.20	5638.11	56036.53	1303.27	57119.20	14348.65	*-2.74*	14.78	29.46	5.71E-08	7.24E-07
hsa-miR-139-3p:chr11:72326110:72326132	107.90	19.64	155.42	24.63	94.13	18.95	178.18	11.61	30.21	16.58	56.96	21.09	*-2.27*	5.95	28.85	7.82E-08	9.28E-07
hsa-miR-200a-5p:chr1:1103258:1103279	191.27	514.63	104.82	393.16	80.83	152.29	81.65	403.79	130.25	1870.47	87.73	588.49	*2.18*	8.58	28.58	9.00E-08	1.01E-06
hsa-miR-144-5p:chr17:27188601:27188622	272.35	164.75	836.98	286.23	142.43	38.37	627.17	36.96	1202.25	164.46	896.72	65.65	*-2.48*	8.63	27.25	1.78E-07	1.88E-06
hsa-miR-193b-3p:chr16:14397874:14397895	9.15	20.87	9.71	37.04	11.34	28.89	6.70	90.10	7.88	64.23	14.28	40.59	*2.11*	4.85	25.42	4.62E-07	4.39E-06
hsa-miR-326:chr11:75046152:75046171	20.37	316.24	14.97	255.68	73.43	504.70	7.81	11.61	65.67	123.80	6.29	61.48	*2.65*	6.94	24.61	7.03E-07	6.36E-06
hsa-miR-1307-5p:chr10:105154098:105154118	20.17	79.80	111.71	169.37	7.89	74.84	89.46	188.76	39.84	394.19	46.93	204.12	*2.03*	6.90	22.31	2.32E-06	1.84E-05
hsa-let-7c-5p:chr21:17912158:17912179	2581.47	697.31	3043.57	900.13	1923.57	572.91	2934.60	365.31	1490.78	66.82	2385.46	1611.09	*-2.20*	10.60	21.89	2.88E-06	2.19E-05
hsa-miR-130b-3p:chr22:22007643:22007664	3.53	18.17	6.48	15.47	7.89	24.63	6.70	58.03	4.16	56.72	4.42	5.77	*2.10*	4.18	20.74	5.27E-06	3.85E-05
hsa-miR-183-5p:chr7:129414807:129414828	1868.17	24878.90	2768.35	2966.19	1733.33	2736.88	1839.29	7083.76	1680.79	24136.92	850.98	11700.23	*2.39*	12.78	17.12	3.51E-05	2.02E-04
hsa-miR-101-3p:chr9:4850345:4850365	7.90	4.17	224.62	3.82	1.97	2.37	108.62	23.21	7.22	1.29	282.75	26.86	*-2.45*	5.87	14.34	1.53E-04	7.08E-04
