# Supplementary material for: snoRNA and piRNA expression levels modified by tobacco use in women with lung adenocarcinoma
Source: PLoS One. 2017 Aug 17;12(8):e0183410. doi: 10.1371/journal.pone.0183410 (PMC5560661; doi:10.1371/journal.pone.0183410)
Supplement: S6 File — (PDF) [file pone.0183410.s006.pdf]

Supplemental File 6

miRNA analysis

Normal Non-Smoker x Normal Smoker

for the manuscript: “snoRNA and piRNA expression levels modified by tobacco use in women with lung adenocarcinoma” by

Natasha Andressa Nogueira Jorge, Gabriel Wajnberg, Carlos Gil Ferreira, Benilton de Sa Carvalho, Fabio Passetti

We also performed the miRNA differential expression analysis between non-smokers normal and smokers normal samples. The CPM counts were calculated using the EdgeR Bioconductor package and normalized using the TMM methodology. Figure 1 shows the total raw and normalized counts.

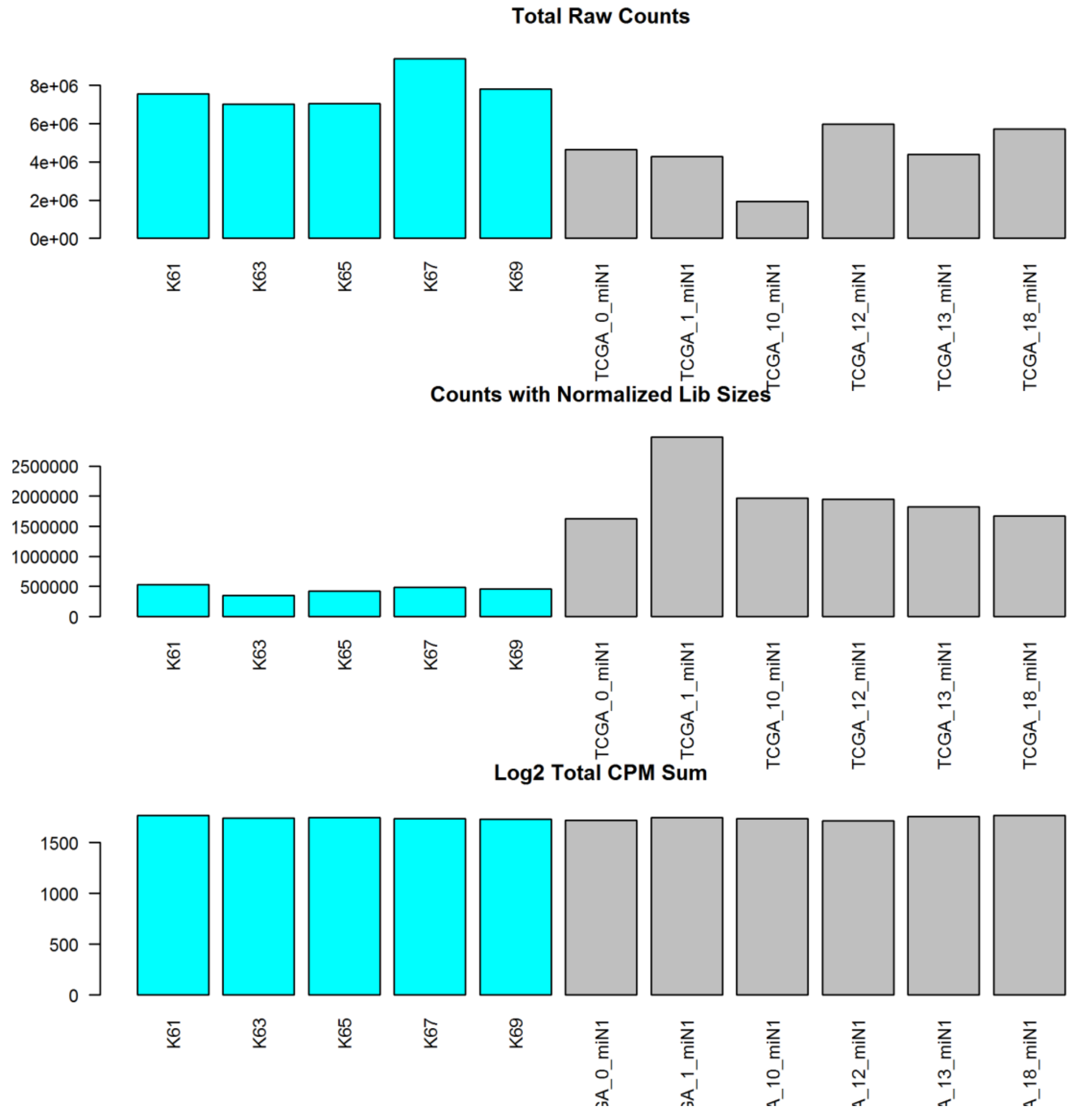

Figure 1. Raw, Normalized and log2 Normalized Total Counts. Light blue indicates non-smokers and light gray bars indicate smokers samples.

Hierarchical clustering was performed on the normalized CPM counts (Figure 2). The miRNAs evaluated allowed the complete distinction between non-smokers and smokers samples.

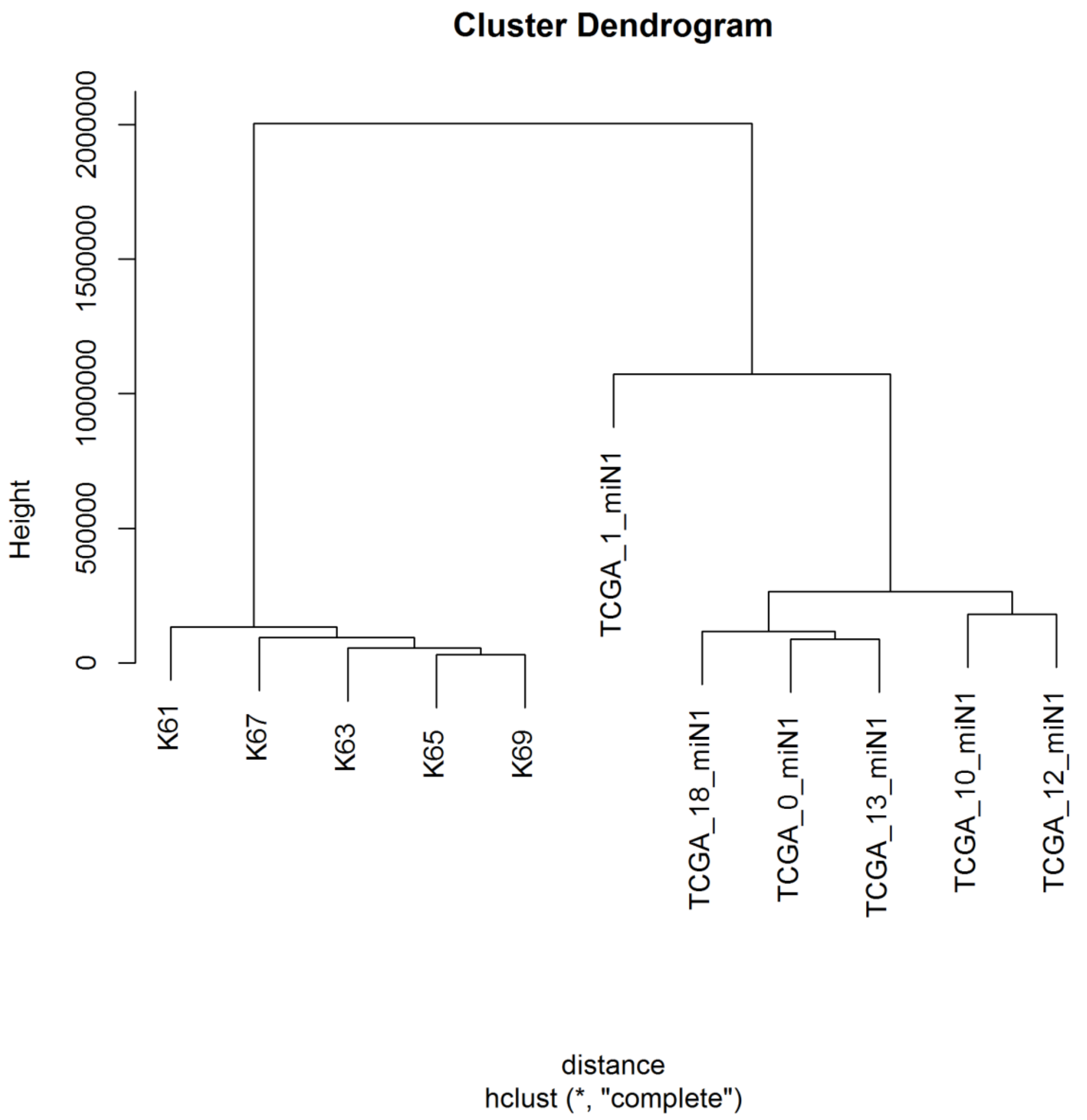

Figure 2. Hierarchical cluster for normalized counts. Samples starting with 'K' correspond to the non-smokers and the ones starting with 'T" belong to samples.

To further investigate the distribution of our samples, we used the normalized counts to perform principal component analysis. This analysis revealed two clearly distinct groups that correspond to the non-smokers and smokers samples (Figure 3).

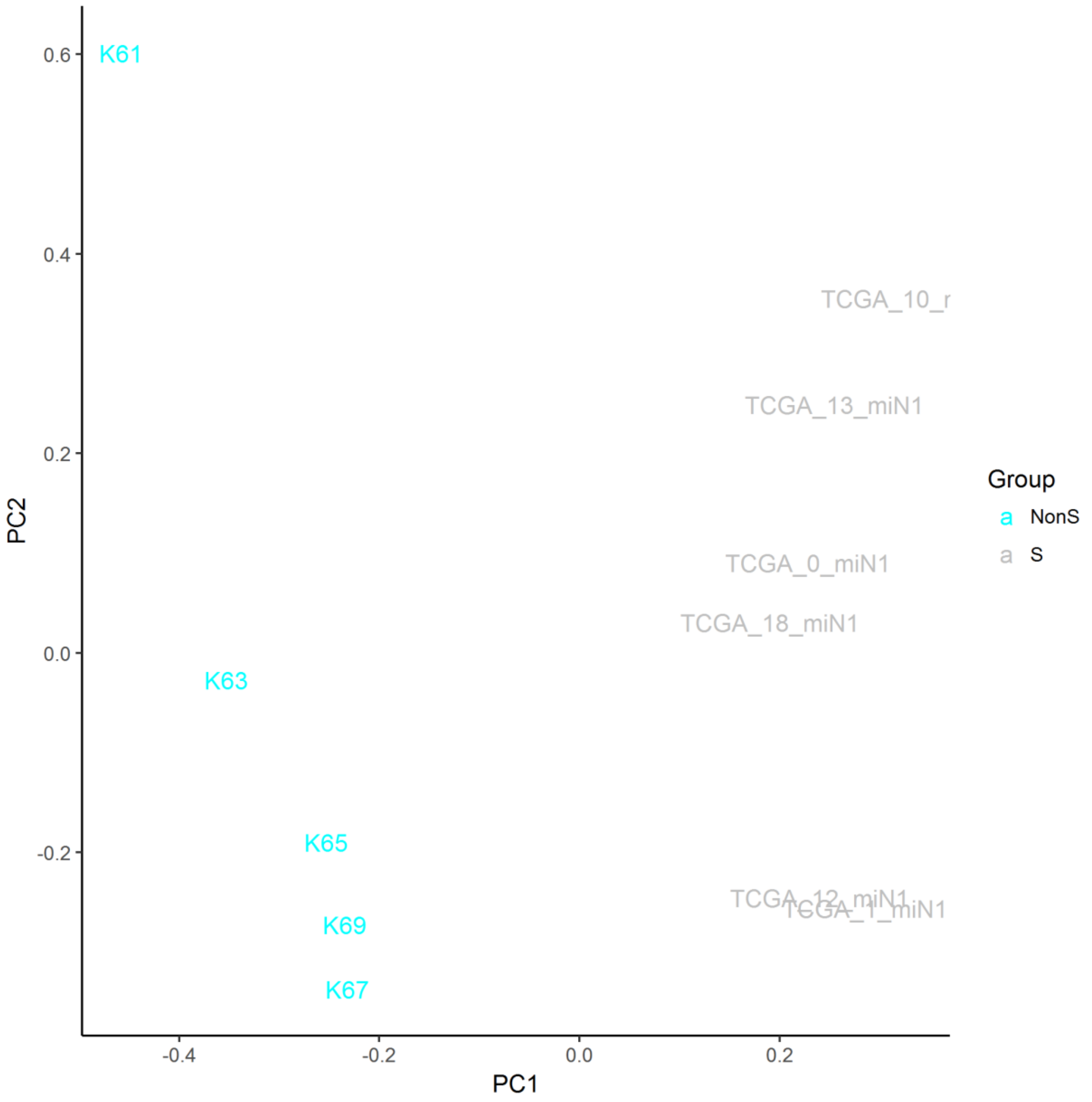

Figure 3. PCA analysis.

After applying our differential expression filters, FDR < 0.01 and logFC > 2 or logFC < -2, we found 130 differentially expressed miRNA (Figure 4). Half of the miRNAs are up-regulated in normal samples and the other half are down-regulated (Figure 5). Table 1 shows cpm, logFC, and FDR for each miRNA.

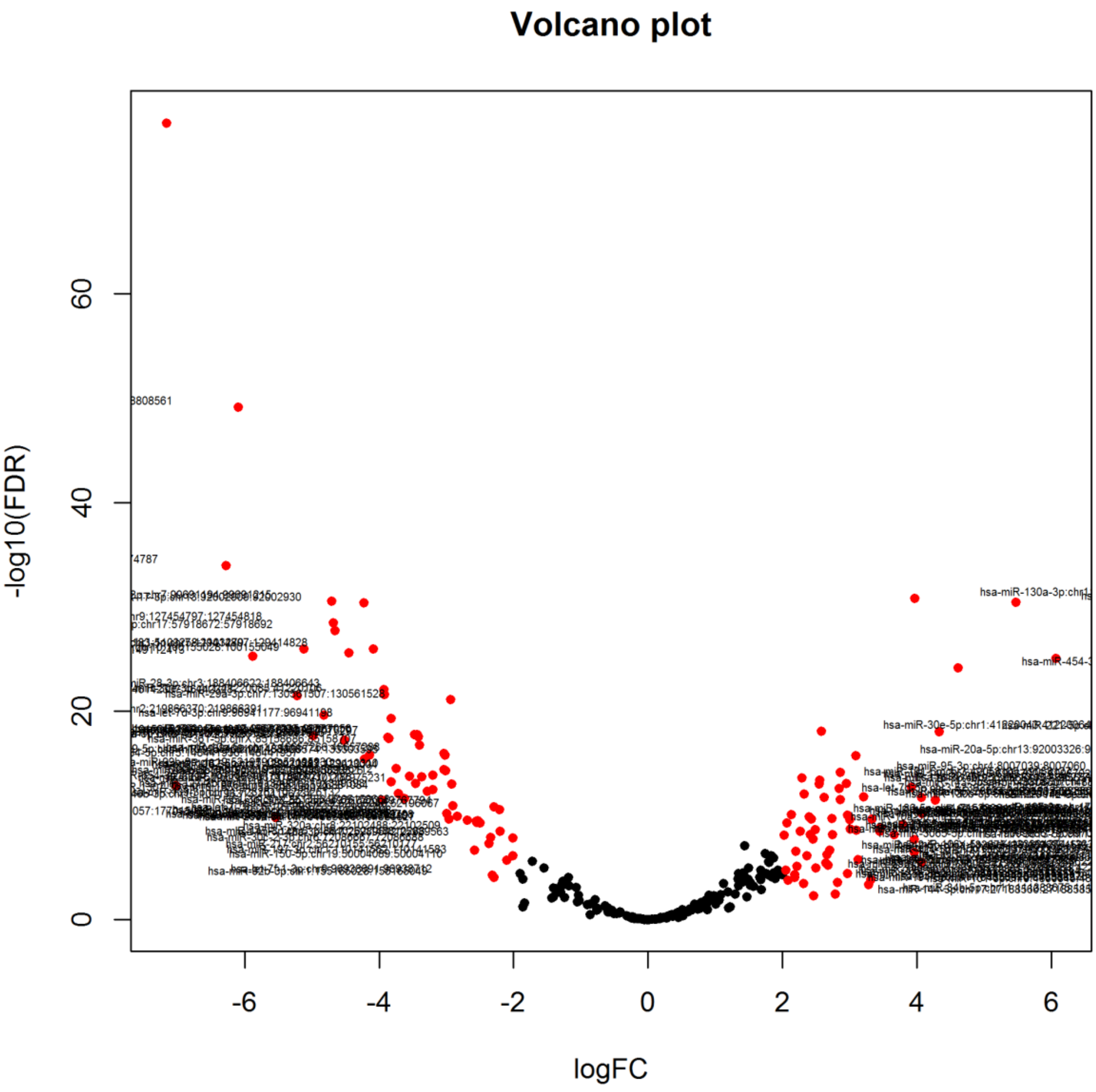

Figure 4. Volcano Plot. The red dots indicate the differentially expressed genes found.

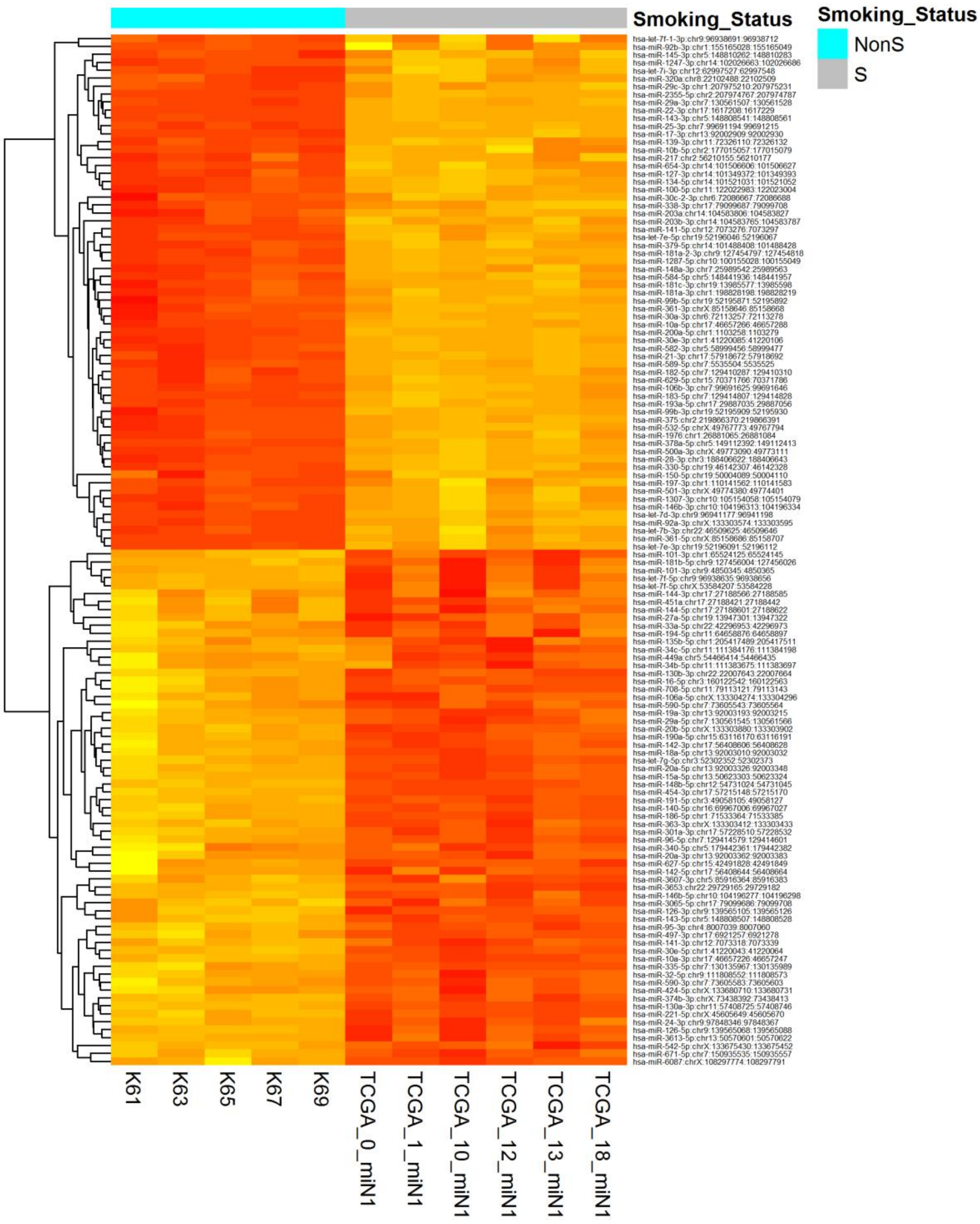

Figure 5. Heatmap. The samples starting with 'K' refer to non-smokers samples and the ones starting with 'T' to smokers samples. A total of 65 miRNAs were found up-regulated in non-smokers samples (yellow area on bottom left of the heatmap) and 65 up-regulated smokers samples (yellow area on top right).

Table 1. Differentially expressed miRNAs.

Gene	K61	K63	K65	K67	K69	TCGA_0_miN1	TCGA_1_miN1	TCGA_10_miN1	TCGA_12_miN1	TCGA_13_miN1	TCGA_18_miN1	logFC	logCPM	PValue	FDR
hsa-miR-22-3p:chr17:1617208:1617229	827.66	744.18	627.21	487.79	471.10	75623.01	104785.32	96635.92	91521.55	94680.66	78304.99	-7.16	15.60	1.52E-79	4.17E-77
hsa-miR-143-3p:chr5:148808541:148808561	14175.69	25833.62	16187.00	14535.57	16788.70	938523.02	2005511.90	1180589.55	1087913.64	976616.93	992197.58	-6.10	19.33	5.29E-52	7.28E-50
hsa-miR-2355-5p:chr2:207974767:207974787	0.49	0.60	0.49	0.51	0.35	17.41	56.50	51.82	36.60	33.57	41.27	-6.28	4.38	1.22E-36	1.12E-34
hsa-miR-130a-3p:chr11:57408725:57408746	1614.14	1407.78	1217.30	1210.84	953.57	67.21	109.52	56.90	87.57	68.80	100.98	3.97	9.29	2.10E-33	1.44E-31
hsa-miR-25-3p:chr7:99691194:99691215	840.12	337.93	574.98	341.62	407.43	10615.14	14205.01	15500.89	11144.52	16682.07	10105.14	-4.70	12.84	5.04E-33	2.77E-31
hsa-miR-148b-5p:chr12:54731024:54731045	99.05	131.84	58.72	74.09	81.66	1.74	0.70	1.02	1.31	2.07	4.10	5.47	5.42	8.03E-33	3.68E-31
hsa-miR-17-3p:chr13:92002909:92002930	31.12	28.52	24.81	31.65	23.16	517.82	463.18	406.45	462.37	799.87	478.84	-4.23	8.21	1.09E-32	4.30E-31
hsa-miR-181a-2-3p:chr9:127454797:127454818	33.02	44.88	40.83	97.05	60.62	1229.26	1999.89	1380.91	1447.88	1431.06	1054.86	-4.69	9.65	9.49E-31	3.26E-29
hsa-miR-21-3p:chr17:57918672:57918692	58.43	18.02	45.20	63.25	48.13	876.85	951.47	1301.65	1290.05	1425.68	1213.20	-4.66	9.37	5.93E-30	1.81E-28
hsa-miR-183-5p:chr7:129414807:129414828	186.98	158.60	201.83	169.55	233.44	3129.21	4771.28	3573.69	3231.34	3182.08	1464.92	-4.08	10.85	4.21E-28	1.08E-26
hsa-miR-200a-5p:chr1:1103258:1103279	3.17	3.77	6.37	5.75	9.85	320.37	180.67	166.64	143.45	246.59	151.03	-5.12	6.81	4.34E-28	1.08E-26
hsa-miR-1287-5p:chr10:100155028:100155049	1.34	1.81	2.00	2.83	2.58	37.61	65.57	48.77	55.55	25.28	48.00	-4.45	4.69	1.15E-27	2.63E-26
hsa-miR-378a-5p:chr5:149112392:149112413	1.55	1.36	3.46	1.70	1.82	79.05	101.84	225.58	78.10	157.90	63.51	-5.88	6.00	2.36E-27	4.99E-26
hsa-miR-3653:chr22:29729165:29729182	52.23	37.20	30.88	56.16	55.40	1.39	1.40	2.03	0.00	0.41	0.00	6.07	4.51	4.27E-27	8.39E-26
hsa-miR-454-3p:chr17:57215148:57215170	180.92	99.00	93.61	63.20	82.07	4.18	2.09	3.05	2.94	5.39	6.73	4.61	5.66	3.59E-26	6.58E-25
hsa-miR-28-3p:chr3:188406622:188406643	190.42	253.93	494.72	392.64	394.07	4126.20	6061.06	8326.08	4146.60	4726.28	4172.89	-3.93	11.56	4.58E-24	7.88E-23
hsa-miR-30e-3p:chr1:41220085:41220106	538.33	782.03	1049.02	1418.15	1549.65	15949.35	20865.27	17377.66	12748.26	19763.44	10437.93	-3.92	13.19	1.59E-23	2.58E-22
hsa-miR-330-5p:chr19:46142307:46142328	0.56	1.36	2.43	2.88	1.99	58.15	82.31	113.81	61.10	74.18	28.98	-5.22	5.24	2.00E-23	3.06E-22
hsa-miR-29a-3p:chr7:130561507:130561528	3822.36	3414.85	2942.55	2403.75	2889.73	21312.47	26076.00	23979.38	19061.28	24014.36	27838.69	-2.94	13.81	5.50E-23	7.96E-22
hsa-miR-375:chr2:219866370:219866391	130.23	264.17	476.16	548.21	574.39	6802.01	11613.59	17483.34	6399.29	16595.87	8879.94	-4.82	12.63	1.76E-21	2.42E-20
hsa-let-7d-3p:chr9:96941177:96941198	49.42	45.38	79.29	42.44	45.20	643.88	588.74	1291.49	381.33	718.23	835.05	-3.83	8.74	3.93E-21	5.14E-20
hsa-miR-30e-5p:chr1:41220043:41220064	11611.47	10875.71	11609.90	14549.03	13199.25	2046.21	2029.89	1473.37	1985.40	2701.74	2168.54	2.58	12.72	6.48E-20	8.10E-19
hsa-miR-221-5p:chrX:45605649:45605670	76.66	121.75	41.19	59.70	63.08	1.39	8.37	2.03	3.59	2.49	4.10	4.33	5.17	7.36E-20	8.80E-19
hsa-miR-193a-5p:chr17:29887035:29887056	23.94	17.47	30.51	20.65	24.33	204.76	408.07	230.66	301.93	201.83	167.13	-3.43	7.21	1.62E-19	1.80E-18
hsa-miR-500a-3p:chrX:49773090:49773111	17.04	16.32	23.72	27.18	25.62	158.45	242.75	358.69	207.82	330.72	175.32	-3.48	7.16	1.64E-19	1.80E-18
hsa-miR-134-5p:chr14:101521031:101521052	2.46	2.86	6.01	10.64	8.32	152.53	214.15	349.54	213.70	109.83	110.93	-4.98	6.73	2.03E-19	2.15E-18

hsa-miR-141-5p:chr12:7073276:7073297	20.34	23.80	23.42	27.69	30.13	320.72	349.48	131.08	273.50	331.14	198.44	*-3.41*	7.29	2.86E-19	2.91E-18
hsa-miR-582-3p:chr5:58999456:58999477	13.16	12.55	25.78	32.52	43.50	298.09	486.20	327.19	383.62	436.41	312.01	*-3.87*	7.75	3.31E-19	3.25E-18
hsa-miR-106b-3p:chr7:99691625:99691646	20.70	13.45	32.58	24.51	21.81	186.30	456.90	402.38	460.08	292.18	172.10	*-3.86*	7.56	4.53E-19	4.29E-18
hsa-miR-30a-3p:chr6:72113257:72113278	526.36	1605.73	2285.42	2882.08	3372.08	24693.10	78021.53	51200.18	40487.01	55135.55	43146.41	*-4.51*	14.75	6.11E-19	5.60E-18
hsa-miR-361-5p:chrX:85158686:85158707	54.98	57.68	66.00	60.78	55.22	589.21	470.85	1150.25	344.08	730.66	457.18	*-3.40*	8.52	1.94E-18	1.72E-17
hsa-miR-10a-5p:chr17:46657266:46657288	3661.22	6887.43	7666.15	9375.05	9912.11	50950.45	81291.67	79361.91	56431.32	49157.24	52636.90	*-3.04*	15.18	1.34E-17	1.15E-16
hsa-miR-379-5p:chr14:101488408:101488428	28.09	31.08	41.43	92.22	55.75	842.37	1367.91	737.70	1380.57	496.09	447.52	*-4.14*	8.97	1.94E-17	1.61E-16
hsa-miR-92a-3p:chrX:133303574:133303595	4.01	2.41	5.16	3.90	3.81	23.68	25.11	46.74	22.87	38.96	33.07	*-3.02*	4.20	2.07E-17	1.67E-16
hsa-miR-20a-5p:chr13:92003326:92003348	2339.23	1237.44	1298.53	1196.09	1180.50	149.74	136.72	109.74	125.80	295.08	200.79	*3.09*	9.56	2.23E-17	1.75E-16
hsa-miR-584-5p:chr5:148441936:148441957	2.82	2.91	7.70	11.61	6.27	103.08	125.56	87.39	169.59	154.17	63.22	*-4.22*	6.05	5.10E-17	3.90E-16
hsa-miR-99b-3p:chr19:52195909:52195930	1.62	4.52	7.83	7.86	7.86	88.45	74.64	116.85	73.19	80.82	47.12	*-3.75*	5.51	4.00E-16	2.97E-15
hsa-miR-182-5p:chr7:129410287:129410310	1041.32	609.53	1557.14	824.42	1461.78	8892.44	11548.72	8265.11	9108.14	11366.87	4891.74	*-3.04*	12.40	5.25E-16	3.80E-15
hsa-miR-100-5p:chr11:122022983:122023004	1529.24	1993.81	2264.56	3576.98	2974.03	20182.11	24175.17	31435.66	13559.93	17502.25	12958.29	*-3.02*	13.55	6.66E-16	4.70E-15
hsa-miR-95-3p:chr4:8007039:8007060	57.30	84.09	44.59	55.49	47.54	11.84	5.58	7.11	9.80	4.56	7.90	*2.86*	4.98	1.01E-15	6.95E-15
hsa-let-7e-3p:chr19:52196091:52196112	3.10	3.92	4.19	4.32	3.52	36.91	21.62	71.13	21.24	31.91	31.32	*-3.20*	4.35	1.94E-15	1.30E-14
hsa-miR-99b-5p:chr19:52195871:52195892	741.85	2508.36	3376.06	4340.16	3931.99	27266.18	47807.63	30133.00	45138.78	32961.72	26403.92	*-3.55*	14.32	2.55E-15	1.67E-14
hsa-miR-589-5p:chr7:5535504:5535525	4.86	2.41	7.77	10.07	7.86	56.41	73.94	65.03	43.13	97.39	72.29	*-3.36*	5.30	2.96E-15	1.90E-14
hsa-miR-191-5p:chr3:49058105:49058127	1955.28	1671.40	1404.69	1360.09	1540.10	254.21	366.91	338.37	235.59	402.01	353.86	*2.29*	9.81	4.03E-15	2.52E-14
hsa-miR-140-5p:chr16:69967006:69967027	700.03	729.87	371.27	445.30	386.80	95.42	76.03	104.66	68.95	102.78	90.73	*2.55*	8.18	6.74E-15	4.12E-14
hsa-miR-654-3p:chr14:101506606:101506627	1.97	4.57	2.37	3.85	2.76	61.99	38.37	90.43	33.66	21.97	21.37	*-3.82*	4.63	9.77E-15	5.84E-14
hsa-miR-629-5p:chr15:70371766:70371786	7.39	3.36	15.65	6.27	9.50	63.38	137.42	104.66	86.26	100.29	66.73	*-3.46*	5.75	1.46E-14	8.53E-14
hsa-miR-146b-5p:chr10:104196277:104196298	20131.23	12174.49	17037.04	21021.25	16681.54	1642.96	1997.80	2942.68	1095.63	4331.74	1505.02	*2.95*	13.16	1.51E-14	8.68E-14
hsa-miR-126-3p:chr9:139565105:139565126	13703.96	31319.65	23545.40	29726.12	25266.53	2242.26	4647.81	4575.58	3871.47	4879.21	5213.70	*2.54*	13.73	1.61E-14	9.01E-14
hsa-miR-29c-3p:chr1:207975210:207975231	708.48	543.46	404.69	297.74	400.52	2191.77	4755.93	2226.32	3584.57	3228.08	5390.78	*-2.92*	11.07	1.65E-14	9.07E-14
hsa-miR-1247-3p:chr14:102026663:102026686	0.35	1.00	2.00	3.29	2.05	65.12	498.75	464.37	73.52	33.57	236.49	*-7.02*	6.95	2.56E-14	1.38E-13
hsa-miR-301a-3p:chr17:57228510:57228532	88.98	52.06	29.67	29.39	26.38	4.18	0.70	4.06	0.98	5.39	2.63	*3.92*	4.54	3.91E-14	2.07E-13
hsa-miR-143-5p:chr5:148808507:148808528	44.56	99.71	123.51	129.68	78.38	12.19	9.07	17.27	12.42	11.60	17.27	*2.84*	5.69	4.87E-14	2.53E-13
hsa-miR-127-3p:chr14:101349372:101349393	55.90	93.18	72.07	173.56	86.12	1000.47	753.36	1441.87	982.24	725.69	420.30	*-3.20*	9.04	6.01E-14	3.06E-13
hsa-miR-1976:chr1:26881065:26881084	1.41	1.51	4.73	2.77	2.81	18.46	31.39	34.55	17.65	39.79	15.22	*-3.28*	3.88	9.08E-14	4.54E-13

hsa-miR-1307-3p:chr10:105154058:105154079	19.57	21.69	54.42	48.86	31.07	287.99	380.87	909.43	296.70	687.14	216.88	*-3.72*	8.06	1.61E-13	7.92E-13
hsa-let-7g-5p:chr3:52302352:52302373	14237.01	10041.16	8106.03	5809.12	7514.24	1923.63	1912.00	1348.39	1605.38	2274.87	1933.81	*2.32*	12.33	1.77E-13	8.54E-13
hsa-miR-20b-5p:chrX:133303880:133303902	137.13	64.86	108.35	49.84	66.13	4.88	5.58	4.06	10.78	12.85	15.51	*3.21*	5.48	3.57E-13	1.69E-12
hsa-miR-190a-5p:chr15:63116170:63116191	561.42	122.60	220.21	88.47	130.26	8.71	5.58	7.11	18.63	22.79	16.39	*4.06*	6.79	3.83E-13	1.79E-12
hsa-miR-15a-5p:chr13:50623303:50623324	1472.78	835.75	646.44	649.99	659.46	102.03	136.72	86.37	131.36	204.73	171.52	*2.62*	8.86	4.08E-13	1.87E-12
hsa-miR-139-3p:chr11:72326110:72326132	8.80	22.99	13.41	16.85	13.07	180.73	267.86	194.08	313.04	57.19	98.05	*-3.62*	6.74	5.04E-13	2.27E-12
hsa-miR-130b-3p:chr22:22007643:22007664	119.68	81.38	53.63	52.61	48.25	5.92	11.16	16.26	11.76	7.87	7.61	*2.86*	5.28	6.94E-13	3.08E-12
hsa-miR-146b-3p:chr10:104196313:104196334	2.39	0.95	4.00	6.27	3.17	32.39	39.76	106.69	33.66	72.94	29.85	*-3.95*	4.87	7.42E-13	3.24E-12
hsa-miR-142-3p:chr17:56408606:56408628	27958.42	4626.12	4709.16	5285.78	4159.22	380.27	234.38	307.88	437.21	1040.25	505.77	*4.27*	12.14	8.14E-13	3.50E-12
hsa-miR-361-3p:chrX:85158646:85158668	12.18	20.28	45.07	34.22	31.54	134.42	296.46	268.26	191.16	198.52	201.66	*-2.91*	7.02	2.72E-12	1.15E-11
hsa-miR-532-5p:chrX:49767773:49767794	120.73	152.17	231.80	224.52	211.34	663.38	927.75	1146.18	977.02	1090.39	720.31	*-2.29*	9.20	3.55E-12	1.48E-11
hsa-let-7e-5p:chr19:52196046:52196067	792.53	1122.87	1124.48	1000.65	990.38	5512.16	6540.28	2971.13	5262.81	4532.32	3144.38	*-2.21*	11.55	6.66E-12	2.73E-11
hsa-miR-497-3p:chr17:6921257:6921278	7.18	10.69	3.34	5.96	2.99	0.35	0.00	0.00	0.65	0.41	0.29	*4.07*	1.89	1.53E-11	6.18E-11
hsa-let-7i-3p:chr12:62997527:62997548	13.09	9.19	13.35	8.17	8.09	37.26	145.79	120.92	59.47	47.66	87.81	*-2.99*	5.62	1.61E-11	6.42E-11
hsa-miR-186-5p:chr1:71533364:71533385	1737.12	1507.79	885.16	928.77	900.81	239.58	295.76	260.13	206.84	354.35	278.64	*2.13*	9.43	2.10E-11	8.25E-11
hsa-miR-671-5p:chr7:150935535:150935557	12.67	7.08	15.71	8.63	8.68	1.39	0.70	0.00	1.31	0.83	2.63	*2.97*	2.65	2.44E-11	9.45E-11
hsa-miR-181c-3p:chr19:13985577:13985598	4.58	8.74	10.13	19.83	11.67	65.12	76.73	84.34	76.46	122.67	48.59	*-2.84*	5.57	3.12E-11	1.19E-10
hsa-miR-18a-5p:chr13:92003010:92003032	124.67	40.26	56.96	39.51	37.75	5.22	4.88	4.06	6.21	13.26	10.54	*2.98*	5.01	3.25E-11	1.23E-10
hsa-miR-141-3p:chr12:7073318:7073339	1592.25	2518.70	1923.98	2228.96	2410.77	678.36	310.41	186.97	274.15	536.29	445.48	*2.40*	10.22	3.54E-11	1.31E-10
hsa-miR-10b-5p:chr2:177015057:177015079	758.67	1364.80	850.82	1186.43	592.68	35044.98	27106.29	23817.82	167000.76	5823.72	7283.31	*-5.54*	14.59	3.95E-11	1.45E-10
hsa-miR-96-5p:chr7:129414579:129414601	106.44	51.96	40.95	34.48	36.82	11.14	6.98	10.16	4.25	10.36	6.44	*2.73*	4.91	5.09E-11	1.84E-10
hsa-let-7b-3p:chr22:46509625:46509646	10.35	11.70	20.87	11.46	12.60	106.21	76.03	224.56	49.67	102.78	71.71	*-2.96*	5.97	5.75E-11	2.05E-10
hsa-miR-32-5p:chr9:111808552:111808573	1088.76	793.28	325.59	373.63	364.58	41.79	80.92	11.18	61.10	60.51	95.12	*3.33*	8.23	5.96E-11	2.10E-10
hsa-miR-126-5p:chr9:139565068:139565088	3575.20	4038.68	4062.42	4655.52	3615.95	367.73	1106.32	322.11	1090.08	667.66	891.83	*2.43*	11.12	6.53E-11	2.27E-10
hsa-miR-363-3p:chrX:133303412:133303433	166.28	97.45	145.41	64.38	88.87	9.05	9.07	16.26	4.90	27.77	17.27	*3.00*	5.90	7.05E-11	2.42E-10
hsa-miR-203b-3p:chr14:104583765:104583787	4.72	5.07	10.98	6.47	11.31	77.31	34.18	49.79	36.92	69.63	31.03	*-2.69*	4.92	7.96E-11	2.70E-10
hsa-miR-338-3p:chr17:79099687:79099708	116.30	171.75	262.62	214.25	218.67	618.11	926.35	676.73	1271.75	1610.10	1727.46	*-2.53*	9.47	9.11E-11	3.06E-10
hsa-miR-181a-3p:chr1:198828198:198828219	32.38	43.18	52.96	98.18	72.40	195.01	502.94	350.56	315.00	397.86	350.94	*-2.56*	7.77	1.02E-10	3.37E-10
hsa-miR-501-3p:chrX:49774380:49774401	5.98	3.82	6.55	5.96	5.98	23.68	34.18	61.98	21.24	43.10	18.44	*-2.55*	4.34	1.35E-10	4.41E-10

hsa-miR-203a:chr14:104583806:104583827	455.61	528.30	1335.84	1004.30	1724.53	5261.43	6378.44	4462.79	6619.53	6079.02	5500.24	*-2.50*	11.80	1.52E-10	4.91E-10
hsa-miR-10a-3p:chr17:46657226:46657247	12.60	15.71	14.68	15.62	12.72	4.18	3.49	2.03	2.94	2.90	4.10	*2.07*	3.18	1.61E-10	5.16E-10
hsa-miR-6087:chrX:108297774:108297791	5.07	7.88	27.18	5.86	7.68	0.70	0.70	0.00	0.65	1.24	0.88	*3.80*	2.62	2.19E-10	6.93E-10
hsa-miR-19a-3p:chr13:92003193:92003215	244.70	82.79	66.49	54.82	48.42	10.10	6.28	4.06	3.27	9.95	19.61	*3.45*	5.68	4.44E-10	1.39E-09
hsa-miR-101-3p:chr1:65524125:65524145	12.18	12.70	18.99	21.58	25.74	1.04	6.28	1.02	2.29	0.41	2.93	*3.01*	3.38	6.66E-10	2.06E-09
hsa-miR-29a-5p:chr7:130561545:130561566	52.16	28.72	26.45	21.99	21.10	5.22	4.19	2.03	3.59	4.97	10.54	*2.49*	4.12	7.67E-10	2.34E-09
hsa-miR-590-3p:chr7:73605583:73605603	95.67	38.51	25.30	21.32	18.64	3.83	4.88	1.02	6.54	3.73	6.73	*3.10*	4.43	7.85E-10	2.37E-09
hsa-miR-16-5p:chr3:160122542:160122563	24.29	17.02	6.31	4.88	6.04	0.70	1.40	1.02	1.31	0.83	1.17	*3.45*	2.76	1.04E-09	3.10E-09
hsa-miR-335-5p:chr7:130135967:130135989	338.54	376.63	133.58	153.93	170.48	51.89	59.99	36.58	48.03	44.76	52.10	*2.26*	7.07	1.05E-09	3.11E-09
hsa-miR-320a:chr8:22102488:22102509	298.13	367.34	198.56	168.62	164.15	1021.36	1265.36	1723.34	750.57	921.72	938.37	*-2.20*	9.47	1.15E-09	3.36E-09
hsa-miR-627-5p:chr15:42491828:42491849	50.76	7.63	8.43	12.28	9.97	1.04	1.40	2.03	1.63	2.07	0.59	*3.66*	3.29	2.25E-09	6.53E-09
hsa-miR-24-3p:chr9:97848346:97848367	35.48	42.67	18.87	20.91	23.21	1.74	4.88	1.02	4.25	2.49	9.66	*2.74*	4.00	2.32E-09	6.65E-09
hsa-miR-708-5p:chr11:79113121:79113143	87.93	51.91	28.51	25.07	30.66	7.31	9.07	12.19	7.52	7.87	7.61	*2.41*	4.70	2.41E-09	6.84E-09
hsa-miR-374b-3p:chrX:73438392:73438413	16.19	17.07	11.95	15.36	14.13	3.13	4.88	2.03	4.90	2.07	4.39	*2.02*	3.25	2.62E-09	7.34E-09
hsa-miR-145-3p:chr5:148810262:148810283	24.99	38.36	32.33	35.61	19.70	82.53	229.50	136.16	185.27	85.79	200.49	*-2.34*	6.60	4.48E-09	1.24E-08
hsa-miR-148a-3p:chr7:25989542:25989563	5479.94	6465.26	8097.60	12063.54	10142.44	30858.89	42555.74	35150.59	22512.21	48947.95	24289.52	*-2.01*	14.45	5.15E-09	1.42E-08
hsa-miR-3065-5p:chr17:79099686:79099708	22.10	40.87	51.93	35.81	62.38	8.01	2.79	13.21	5.23	12.43	5.85	*2.45*	4.62	6.82E-09	1.86E-08
hsa-miR-3613-5p:chr13:50570601:50570622	68.21	78.82	48.35	47.42	42.56	0.00	8.37	0.00	5.88	1.24	6.15	*3.95*	4.86	7.85E-09	2.12E-08
hsa-miR-30c-2-3p:chr6:72086667:72086688	28.72	111.86	87.72	86.42	101.42	195.01	620.13	495.87	364.99	455.47	453.96	*-2.37*	8.09	1.80E-08	4.80E-08
hsa-miR-217:chr2:56210155:56210177	1.69	3.36	2.79	10.22	3.52	32.04	24.41	26.42	23.20	11.60	38.05	*-2.58*	3.98	8.14E-08	2.13E-07
hsa-miR-106a-5p:chrX:133304274:133304296	59.91	21.54	25.48	13.77	14.95	1.39	1.40	6.10	4.25	5.80	6.15	*2.70*	3.95	8.30E-08	2.15E-07
hsa-miR-542-5p:chrX:133675430:133675452	9.79	7.68	8.86	7.91	6.21	2.44	1.40	2.03	2.94	0.41	1.17	*2.20*	2.41	1.07E-07	2.75E-07
hsa-miR-3607-3p:chr5:85916364:85916383	107.85	16.42	46.53	71.98	43.67	2.09	0.70	18.29	0.98	2.07	0.88	*3.95*	4.87	1.32E-07	3.36E-07
hsa-miR-181b-5p:chr9:127456004:127456026	15.21	16.67	14.13	25.64	13.66	1.74	3.49	0.00	2.94	0.41	6.44	*2.66*	3.33	2.18E-07	5.46E-07
hsa-miR-424-5p:chrX:133680710:133680731	595.77	452.79	168.95	179.00	189.65	62.68	81.61	20.32	80.06	48.49	76.68	*2.36*	7.48	2.83E-07	6.94E-07
hsa-miR-197-3p:chr1:110141562:110141583	80.68	70.54	97.79	71.11	69.41	185.26	290.18	608.66	158.48	290.11	358.25	*-2.01*	7.69	2.85E-07	6.94E-07
hsa-miR-451a:chr17:27188421:27188442	97327.63	18042.87	52192.76	8278.12	35868.17	1494.26	3104.12	1474.39	5697.41	7798.95	9575.37	*3.12*	14.42	7.40E-07	1.77E-06
hsa-miR-150-5p:chr19:50004089:50004110	354.38	83.49	257.22	195.24	162.50	427.98	1359.53	1053.71	1126.02	787.85	672.90	*-2.10*	9.20	8.22E-07	1.95E-06
hsa-miR-34c-5p:chr11:111384176:111384198	12053.14	4507.64	2958.38	5255.21	5799.85	1534.66	72.55	142.26	18.63	162.05	255.81	*4.07*	11.54	1.30E-06	3.01E-06

hsa-miR-142-5p:chr17:56408644:56408664	1463.56	277.78	286.22	300.26	272.72	35.17	166.72	42.68	106.20	77.91	70.54	*2.64*	8.14	1.60E-06	3.64E-06
hsa-let-7f-5p:chr9:96938635:96938656	192.26	223.46	198.37	162.92	208.17	7.66	53.71	5.08	48.69	11.19	57.95	*2.67*	6.75	2.52E-06	5.68E-06
hsa-miR-27a-5p:chr19:13947301:13947322	319.96	124.96	212.57	132.15	200.84	13.23	19.53	37.60	70.91	33.98	79.32	*2.22*	6.83	3.10E-06	6.93E-06
hsa-miR-144-5p:chr17:27188601:27188622	12542.97	3305.20	7895.70	2072.72	7501.69	456.18	1442.54	293.66	1101.84	2276.11	1543.65	*2.49*	11.84	4.81E-06	1.05E-05
hsa-miR-590-5p:chr7:73605543:73605564	41.60	20.99	13.41	10.02	9.03	3.48	2.79	6.10	3.59	7.05	4.68	*2.05*	3.57	8.24E-06	1.77E-05
hsa-miR-33a-5p:chr22:42296953:42296973	31.82	17.82	6.61	7.14	4.87	0.00	1.40	0.00	2.94	0.83	4.39	*2.96*	3.00	1.75E-05	3.56E-05
hsa-miR-340-5p:chr5:179442361:179442382	868.21	684.54	128.00	162.66	200.79	91.24	134.63	49.79	56.86	118.94	88.10	*2.18*	7.88	2.46E-05	4.87E-05
hsa-let-7f-1-3p:chr9:96938691:96938712	5.35	2.36	4.73	3.60	2.87	26.81	6.98	30.48	5.55	37.71	6.44	*-2.32*	3.53	2.74E-05	5.34E-05
hsa-miR-20a-3p:chr13:92003362:92003383	13.38	6.73	3.52	3.24	3.75	1.39	1.40	1.02	0.33	2.07	1.76	*2.18*	2.05	4.20E-05	8.03E-05
hsa-miR-92b-3p:chr1:155165028:155165049	34.71	33.74	41.86	38.48	35.23	549.86	91.38	206.27	53.59	92.42	89.86	*-2.29*	6.84	4.56E-05	8.54E-05
hsa-miR-449a:chr5:54466414:54466435	160.51	17.62	16.20	13.05	24.50	16.37	0.70	1.02	0.33	4.56	4.10	*3.32*	4.62	7.70E-05	1.39E-04
hsa-miR-135b-5p:chr1:205417489:205417511	140.16	81.73	32.46	60.32	44.26	30.64	11.16	6.10	4.25	31.08	17.27	*2.08*	5.42	9.02E-05	1.61E-04
hsa-let-7f-5p:chrX:53584207:53584228	1192.53	1447.79	941.70	852.94	1062.61	17.06	306.92	12.19	299.64	23.62	277.76	*2.81*	9.19	1.39E-04	2.43E-04
hsa-miR-194-5p:chr11:64658876:64658897	23.16	9.04	10.80	6.22	5.92	0.35	2.79	1.02	2.94	0.00	5.56	*2.32*	2.79	1.90E-04	3.27E-04
hsa-miR-101-3p:chr9:4850345:4850365	1209.92	1397.69	1722.15	1990.56	2527.84	13.23	387.14	4.06	190.83	13.68	486.74	*3.28*	9.82	2.35E-04	4.01E-04
hsa-miR-34b-5p:chr11:111383675:111383697	270.40	29.77	22.26	32.57	30.78	46.66	2.09	4.06	0.33	4.97	8.20	*2.79*	5.40	2.12E-03	3.35E-03
hsa-miR-144-3p:chr17:27188566:27188585	298.55	27.21	162.82	31.34	75.68	2.09	36.97	0.00	42.48	10.78	36.59	*2.46*	6.06	3.11E-03	4.79E-03
