# Supplementary material for: snoRNA and piRNA expression levels modified by tobacco use in women with lung adenocarcinoma
Source: PLoS One. 2017 Aug 17;12(8):e0183410. doi: 10.1371/journal.pone.0183410 (PMC5560661; doi:10.1371/journal.pone.0183410)
Supplement: S7 File — (PDF) [file pone.0183410.s007.pdf]

Supplemental File 7

miRNA analysis

Tumor Non-Smoker x Tumor Smoker

for the manuscript: “snoRNA and piRNA expression levels modified by tobacco use in women with lung adenocarcinoma” by

Natasha Andressa Nogueira Jorge, Gabriel Wajnberg, Carlos Gil Ferreira, Benilton de Sa Carvalho, Fabio Passetti

We also performed the miRNA differential expression analysis between non-smokers tumor and smokers tumor samples. The CPM counts were calculated using the EdgeR Bioconductor package and normalized using the TMM methodology. Figure 1 shows the total raw and normalized counts.

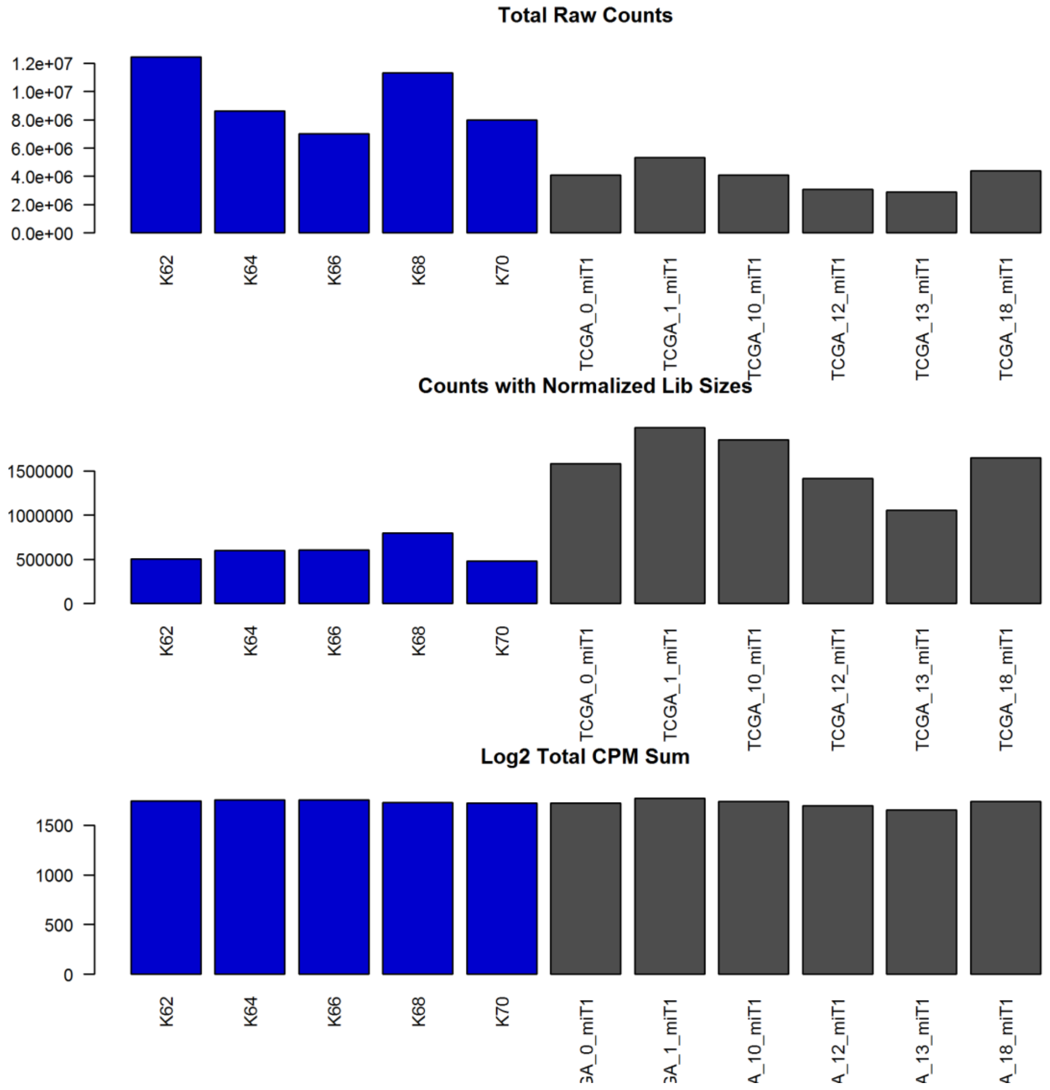

Figure 1. Raw, Normalized and log2 Normalized Total Counts. Dark blue indicates non-smokers and darrk gray bars indicate smokers samples.

Hierarchical clustering was performed on the normalized CPM counts (Figure 2). This is the only clusteriztion that did not completely separated non-smokers and smokers samples. Two smokers samples, TCGA_18 and TCGA_13, were grouped with all non-smokers samples.

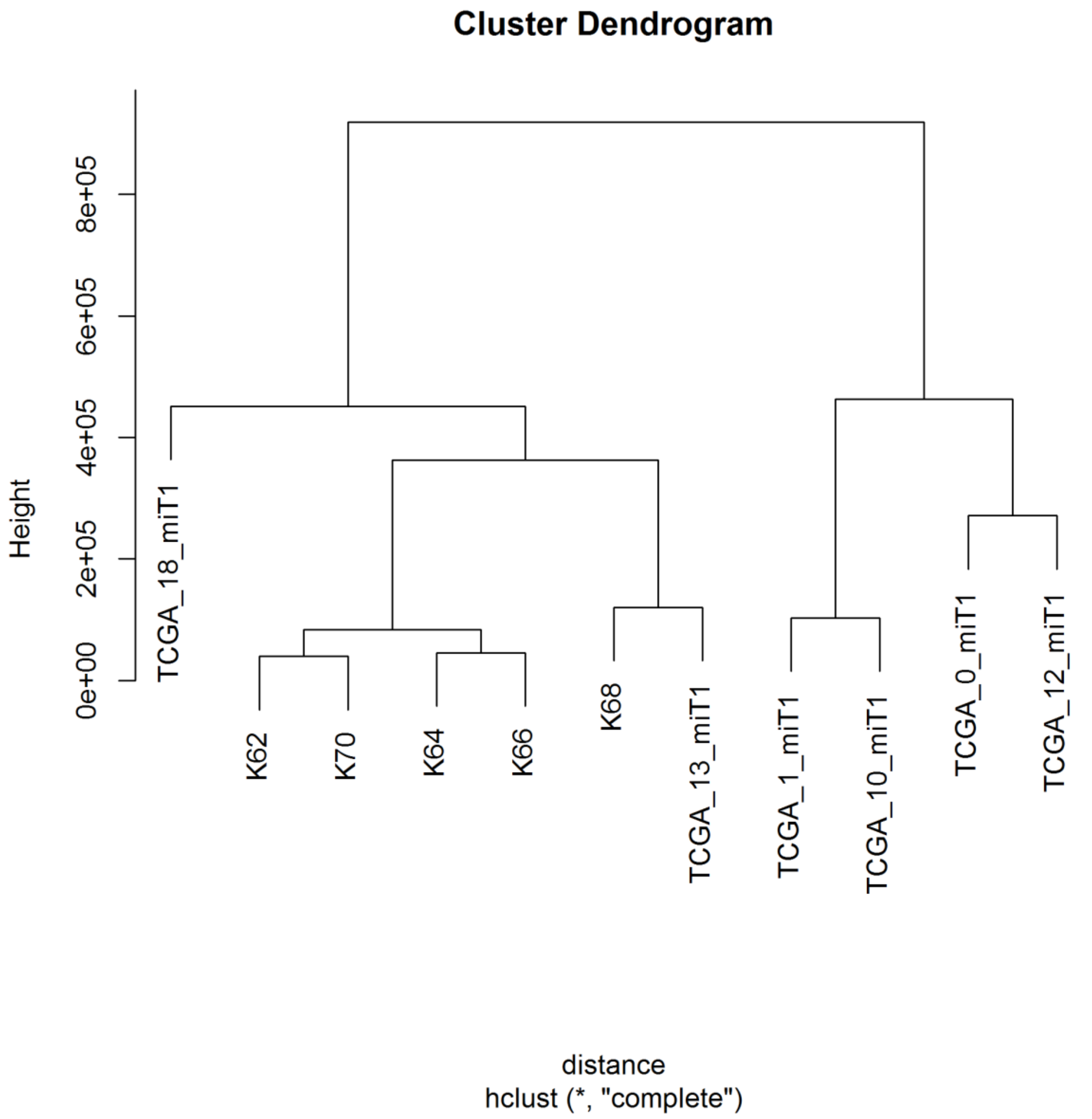

Figure 2. Hierarchical cluster for normalized counts. Samples starting with 'K' correspond to the non-smokers and the ones starting with 'T" belong to smokers samples.

To further investigate the distribution of our samples, we used the normalized counts to perform principal component analysis. Differently from the hierarchical clustering, this analysis was able to clearly distinct the two groups that correspond to the non-smokers and smokers samples (Figure 3).

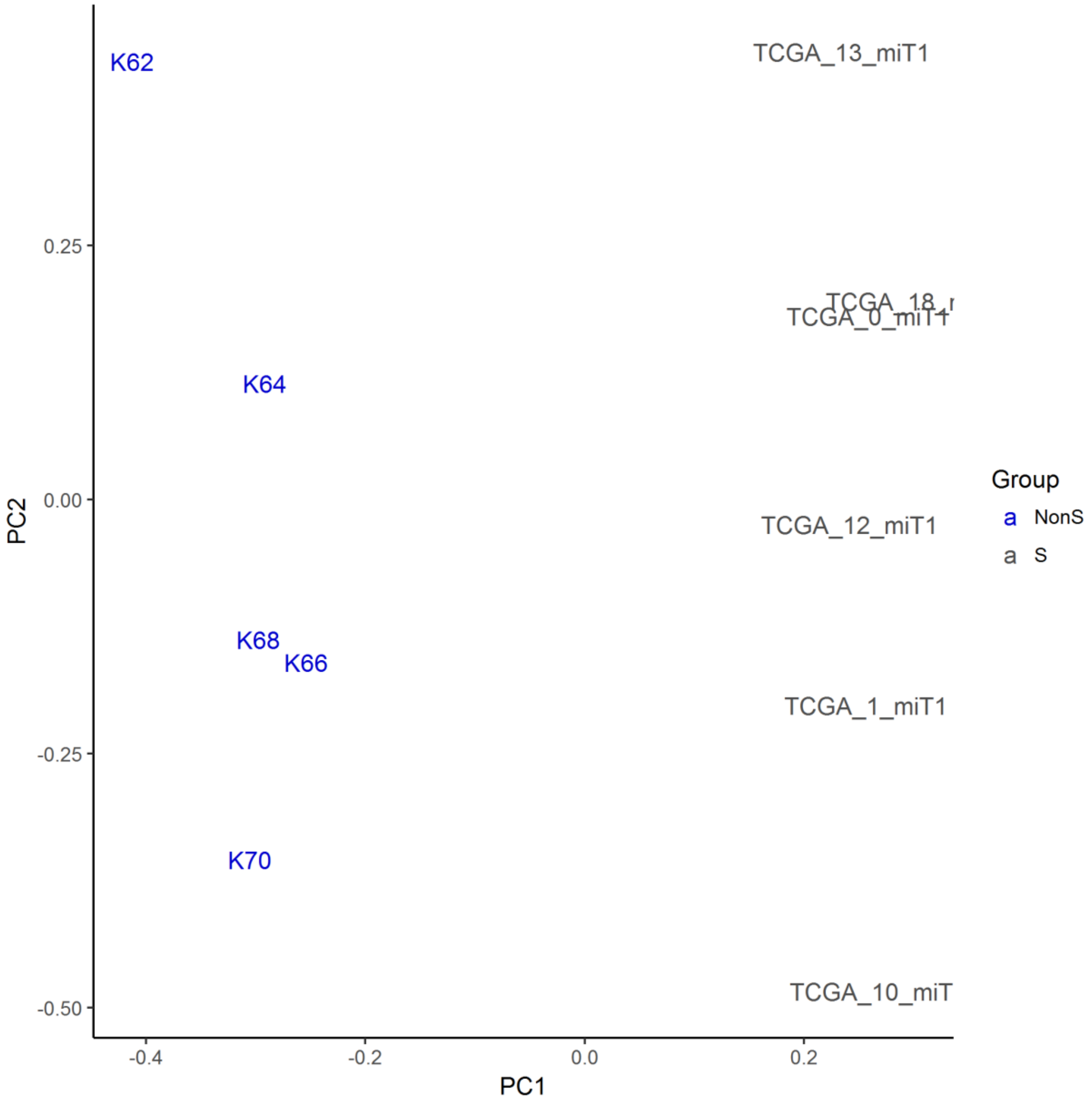

Figure 3. PCA analysis.

After applying our differential expression filters, FDR < 0.01 and logFC > 2 or logFC < -2, we found 135 differentially expressed miRNA (Figure 4). Most miRNAs, 69 miRNAs, are up-regulated in non-smokers samples, while 66 miRNAs are down-regulated (Figure 5). Table 1 shows cpm, logFC, and FDR for each miRNA.

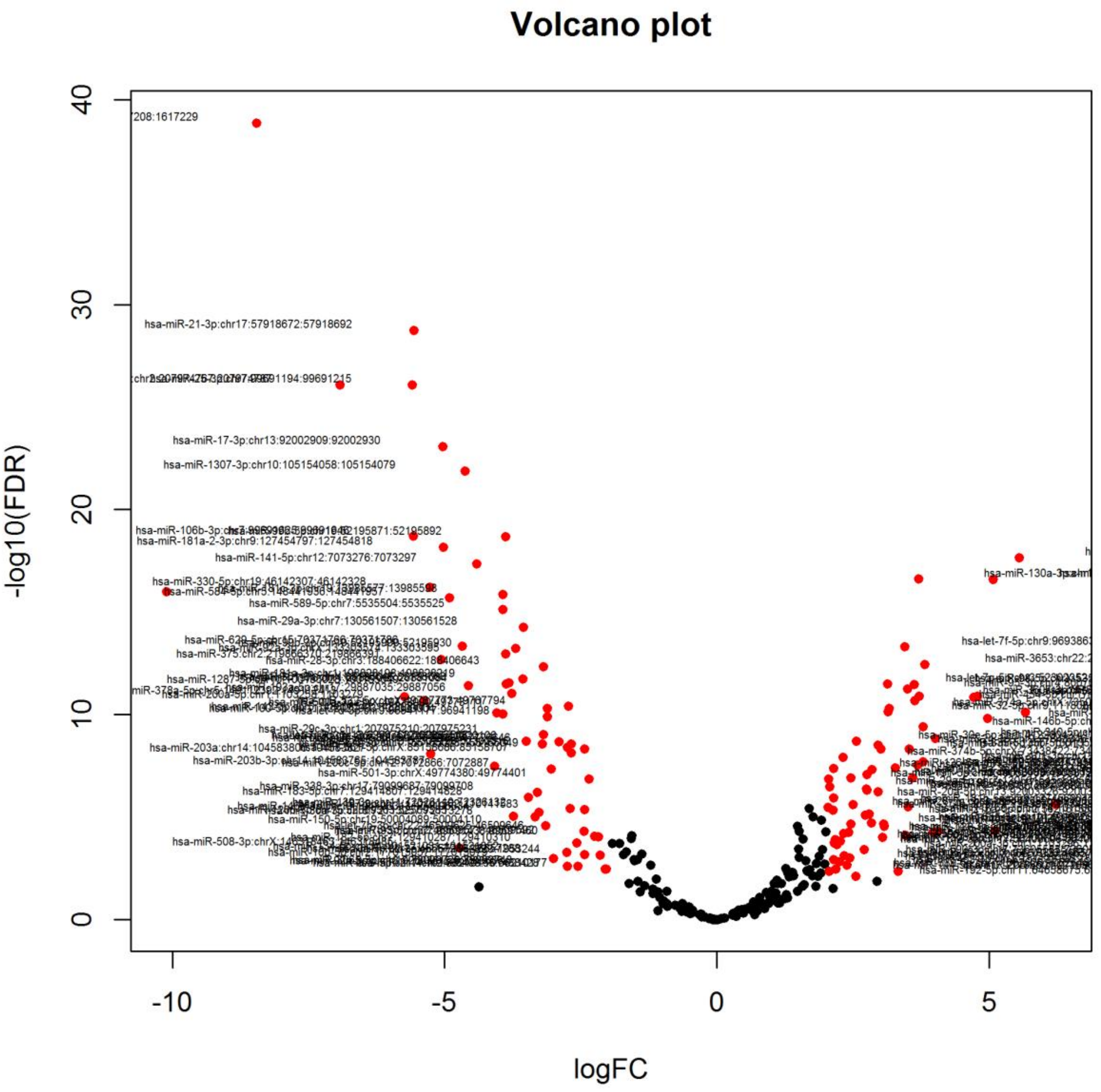

Figure 4. Volcano Plot. The red dots indicate the differentially expressed genes found.

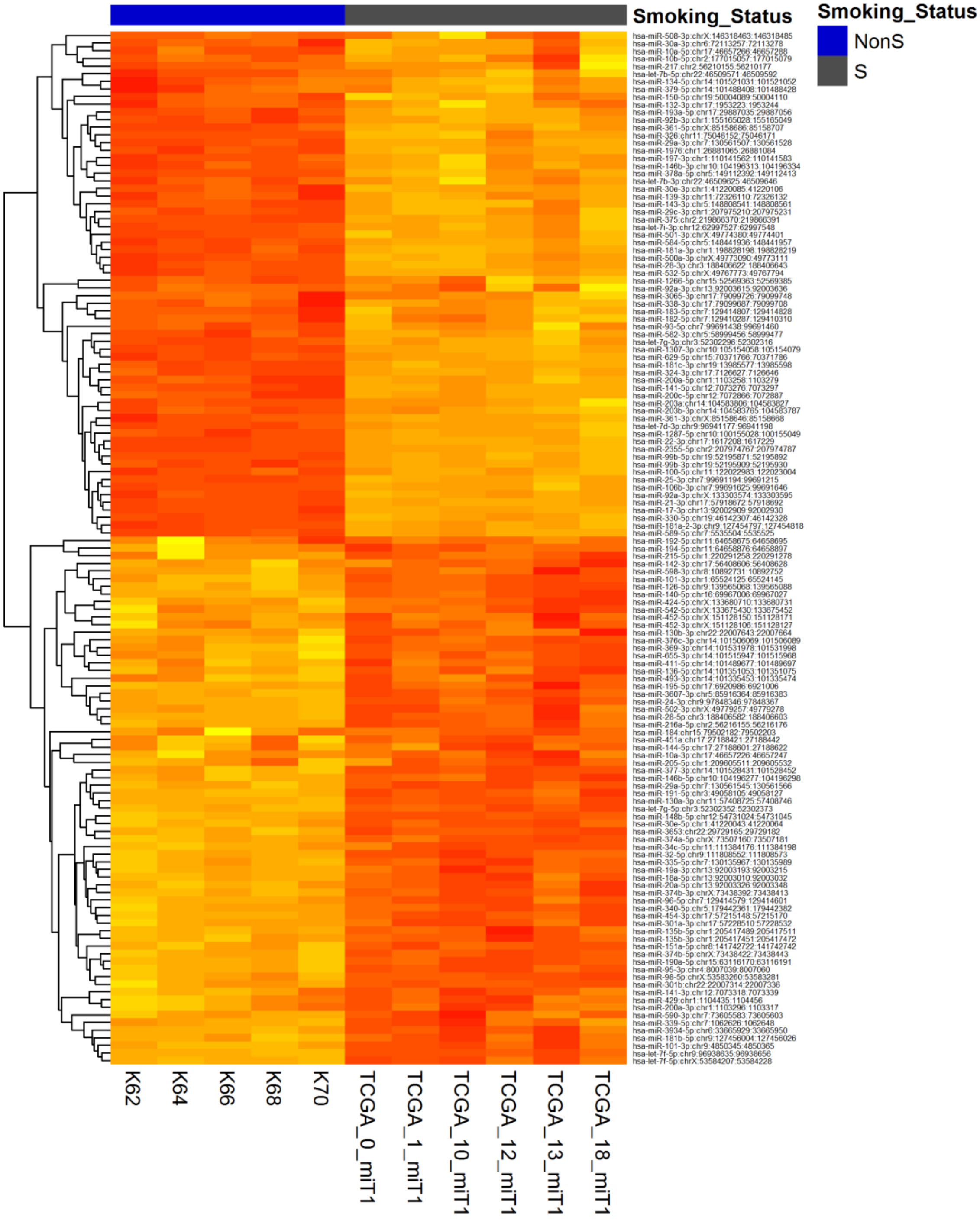

Figure 5. Heatmap. The samples starting with ‘K’ are non-smokers and the ones starting with ‘T’ belong to mokers. A total of 69 miRNAs were found up-regulated in non-smokers samples (yellow area on bottom left of the heatmap) and 66 up-regulated smokers samples (yellow area on top right).

Table 1. Differentially expressed miRNAs

Gene	K62	K64	K66	K68	K70	TCGA_0_miT1	TCGA_1_miT1	TCGA_10_miT1	TCGA_12_miT1	TCGA_13_miT1	TCGA_18_miT1	logFC	logCPM	PValue	FDR
hsa-miR-22-3p:chr17:1617208:1617229	391.81	340.69	392.81	413.03	767.97	98518.54	170214.47	173196.17	156601.48	70233.03	300848.55	-8.45	16.43	5.03E-42	1.40E-39
hsa-miR-21-3p:chr17:57918672:57918692	73.49	140.71	120.74	162.41	77.37	7755.26	4193.09	4993.08	5276.31	6293.81	4046.91	-5.56	11.56	1.28E-31	1.78E-29
hsa-miR-25-3p:chr7:99691194:99691215	390.76	339.50	278.62	263.20	368.03	23130.31	13420.72	11564.99	8425.43	26703.39	12009.44	-5.60	13.10	9.29E-29	8.06E-27
hsa-miR-2355-5p:chr2:207974767:207974787	0.28	0.35	0.26	0.49	0.90	39.48	40.23	43.89	74.02	57.58	89.16	-6.93	4.97	1.16E-28	8.06E-27
hsa-miR-17-3p:chr13:92002909:92002930	20.31	33.71	25.60	30.96	11.88	927.36	999.76	483.29	654.62	979.30	764.46	-5.03	8.81	1.47E-25	8.17E-24
hsa-miR-1307-3p:chr10:105154058:105154079	44.96	63.04	45.50	69.12	36.53	806.99	1001.25	1270.22	1543.34	1975.72	1066.56	-4.62	9.49	2.74E-24	1.27E-22
hsa-miR-106b-3p:chr7:99691625:99691646	14.67	28.00	25.42	24.95	24.71	1307.83	669.73	765.21	750.39	2699.90	533.84	-5.57	9.28	5.02E-21	2.00E-19
hsa-miR-99b-5p:chr19:52195871:52195892	2208.82	2521.21	2957.71	2272.38	1714.80	29141.93	31620.83	27942.51	46566.57	24344.99	46807.18	-3.88	14.28	6.10E-21	2.12E-19
hsa-miR-181a-2-3p:chr9:127454797:127454818	29.85	47.85	75.06	78.87	51.04	1785.83	1505.97	891.01	1768.64	1316.05	3766.26	-5.02	10.01	2.18E-20	6.75E-19
hsa-miR-148b-5p:chr12:54731024:54731045	229.20	124.27	69.38	79.44	38.93	1.94	1.49	1.81	4.16	1.46	3.01	5.55	5.68	7.90E-20	2.20E-18
hsa-miR-141-5p:chr12:7073276:7073297	62.37	44.93	44.99	25.58	21.77	868.92	884.66	390.52	1000.67	987.31	955.20	-4.41	8.91	1.69E-19	4.28E-18
hsa-miR-130a-3p:chr11:57408725:57408746	700.01	730.35	697.37	675.45	1138.52	61.54	75.99	54.30	47.19	94.03	30.47	3.70	8.61	1.04E-18	2.41E-17
hsa-miR-190a-5p:chr15:63116170:63116191	104.27	65.34	48.52	35.69	120.68	2.32	4.47	0.91	0.93	2.19	2.26	5.07	5.17	1.15E-18	2.47E-17
hsa-miR-330-5p:chr19:46142307:46142328	3.95	3.48	5.60	4.95	2.34	308.86	137.82	91.41	56.44	122.46	223.85	-5.27	6.45	3.12E-18	6.19E-17
hsa-miR-326:chr11:75046152:75046171	0.16	0.28	0.34	0.21	0.66	498.52	498.76	964.32	17.58	174.21	116.25	-10.10	7.69	5.46E-18	1.01E-16
hsa-miR-181c-3p:chr19:13985577:13985598	11.44	5.78	9.74	5.02	6.54	100.25	155.33	93.22	89.75	124.64	142.58	-3.93	6.08	7.62E-18	1.32E-16
hsa-miR-584-5p:chr5:148441936:148441957	0.24	1.81	1.12	2.40	1.38	34.83	51.40	42.08	43.02	23.33	56.43	-4.91	4.54	1.21E-17	1.98E-16
hsa-miR-589-5p:chr7:5535504:5535525	9.99	8.85	11.72	19.79	7.56	208.23	155.33	101.36	179.04	195.35	220.46	-3.93	6.67	4.64E-17	7.17E-16
hsa-miR-29a-3p:chr7:130561507:130561528	1789.17	1771.57	2659.27	3003.95	1875.06	30041.81	28657.32	31231.87	11034.67	25928.56	29785.09	-3.56	13.90	3.58E-16	5.24E-15
hsa-miR-629-5p:chr15:70371766:70371786	3.63	8.36	5.08	14.77	9.18	109.15	119.94	135.30	259.54	395.44	238.89	-4.67	6.88	3.27E-15	4.55E-14
hsa-let-7f-5p:chr9:96938635:96938656	103.26	102.88	96.18	123.33	141.61	8.52	8.57	7.24	12.95	6.20	18.81	3.45	5.85	3.73E-15	4.93E-14
hsa-miR-99b-3p:chr19:52195909:52195930	9.79	6.13	8.62	4.17	6.36	71.22	97.59	65.62	112.88	59.77	138.82	-3.69	5.72	4.69E-15	5.93E-14
hsa-miR-92a-3p:chrX:133303574:133303595	1.01	3.55	3.27	2.54	1.62	53.80	37.62	30.32	21.28	37.54	30.10	-3.88	4.33	9.07E-15	1.10E-13
hsa-miR-375:chr2:219866370:219866391	738.97	1118.19	908.69	698.71	678.54	22636.83	13270.98	27791.83	17218.66	7017.62	78700.19	-5.07	13.92	1.78E-14	2.06E-13
hsa-miR-3653:chr22:29729165:29729182	45.08	46.46	24.82	71.60	34.31	1.94	4.84	4.07	4.16	2.92	1.13	3.81	4.50	3.09E-14	3.44E-13
hsa-miR-28-3p:chr3:188406622:188406643	274.97	519.29	668.67	445.54	490.09	4482.00	5525.11	5719.82	3939.76	2561.04	3928.78	-3.18	11.34	4.18E-14	4.46E-13

hsa-miR-181a-3p:chr1:198828198:198828219	28.20	40.82	28.18	77.60	33.53	477.23	660.05	643.48	467.26	221.23	491.71	-3.57	8.17	1.79E-13	1.84E-12
hsa-miR-361-3p:chrX:85158646:85158668	10.76	32.46	26.03	38.17	47.26	356.86	499.88	303.19	371.03	284.64	809.98	-3.82	7.98	2.81E-13	2.79E-12
hsa-miR-1976:chr1:26881065:26881084	2.22	0.84	4.05	2.33	1.86	29.80	53.64	37.56	16.19	26.24	34.99	-3.87	4.25	3.23E-13	3.07E-12
hsa-let-7g-5p:chr3:52302352:52302373	7389.91	5228.26	4484.92	6555.08	7258.84	685.46	1220.64	814.98	361.31	760.62	382.23	3.13	11.64	3.31E-13	3.07E-12
hsa-miR-98-5p:chrX:53583260:53583281	2384.35	1230.83	825.26	412.75	1661.90	133.14	141.92	124.44	84.66	91.84	60.19	3.62	9.35	3.64E-13	3.26E-12
hsa-miR-1287-5p:chr10:100155028:100155049	4.75	4.04	1.03	3.04	2.94	55.73	73.75	43.89	48.58	43.37	186.22	-4.56	5.41	4.24E-13	3.68E-12
hsa-miR-95-3p:chr4:8007039:8007060	73.04	37.96	36.28	27.00	68.26	3.48	5.96	2.72	2.31	6.92	4.14	3.49	4.64	6.45E-13	5.43E-12
hsa-miR-193a-5p:chr17:29887035:29887056	13.86	24.94	34.13	13.36	34.25	256.61	502.86	502.75	292.85	295.94	127.16	-3.77	7.57	1.12E-12	9.14E-12
hsa-miR-3607-3p:chr5:85916364:85916383	45.93	62.20	43.52	93.08	74.25	1.94	3.35	9.05	3.70	2.55	8.65	3.71	5.01	1.54E-12	1.22E-11
hsa-miR-377-3p:chr14:101528431:101528452	5.68	5.22	16.29	9.89	19.97	0.39	0.37	0.91	0.00	0.36	0.38	4.79	2.58	1.59E-12	1.22E-11
hsa-miR-378a-5p:chr5:149112392:149112413	0.68	3.34	3.45	2.26	3.12	129.66	212.69	317.67	45.34	20.77	98.94	-5.74	6.25	1.77E-12	1.33E-11
hsa-let-7f-5p:chrX:53584207:53584228	618.63	886.59	541.13	795.04	985.45	10.45	13.41	9.05	65.69	10.20	66.59	4.71	8.51	1.87E-12	1.37E-11
hsa-miR-454-3p:chr17:57215148:57215170	144.88	73.35	75.24	72.58	43.06	10.45	5.96	3.17	6.94	10.20	2.63	3.63	5.37	2.84E-12	2.02E-11
hsa-miR-200a-5p:chr1:1103258:1103279	24.58	53.43	30.77	7.85	7.32	811.25	766.95	290.97	611.60	2632.11	1112.84	-5.39	9.17	2.92E-12	2.03E-11
hsa-miR-532-5p:chrX:49767773:49767794	92.99	148.99	222.95	196.41	191.03	1086.83	1305.20	1260.26	1070.99	806.54	1249.40	-2.73	9.44	5.78E-12	3.92E-11
hsa-miR-500a-3p:chrX:49773090:49773111	14.58	17.97	25.51	28.27	27.95	277.13	189.60	276.04	173.02	95.12	174.56	-3.11	6.88	7.35E-12	4.87E-11
hsa-miR-374a-5p:chrX:73507160:73507181	497.32	329.96	207.61	317.34	222.88	34.45	69.66	30.32	32.38	17.86	26.33	3.16	7.35	7.55E-12	4.88E-11
hsa-miR-32-5p:chr9:111808552:111808573	668.63	419.61	273.62	400.17	328.09	35.61	42.09	21.27	34.70	77.63	72.61	3.14	7.76	1.10E-11	6.95E-11
hsa-miR-101-3p:chr9:4850345:4850365	438.82	1226.79	1077.34	1284.19	461.12	6.58	7.45	4.53	35.16	1.82	50.79	5.66	8.71	1.25E-11	7.75E-11
hsa-miR-143-3p:chr5:148808541:148808561	8355.75	17977.32	13391.68	8263.12	6734.74	114233.04	265799.22	231214.36	241451.69	29136.50	199440.20	-4.04	16.66	1.31E-11	7.94E-11
hsa-miR-100-5p:chr11:122022983:122023004	583.94	1547.69	1425.17	2930.59	886.79	12765.97	19960.11	21794.16	9104.58	28926.94	43041.30	-3.94	13.67	1.58E-11	9.34E-11
hsa-let-7d-3p:chr9:96941177:96941198	38.23	51.89	66.19	36.33	83.31	297.64	369.88	409.98	402.03	473.07	914.57	-3.11	8.16	2.10E-11	1.21E-10
hsa-miR-301b:chr22:22007314:22007336	56.97	11.91	6.89	7.28	14.16	0.77	0.37	0.91	0.93	0.36	0.38	4.97	3.30	2.70E-11	1.53E-10
hsa-miR-146b-5p:chr10:104196277:104196298	19357.56	10996.78	40929.28	25561.42	24035.56	1601.99	2372.00	3978.08	755.94	1391.86	469.51	3.78	13.54	7.04E-11	3.91E-10
hsa-miR-29c-3p:chr1:207975210:207975231	421.66	283.36	415.65	297.48	234.52	2445.36	3841.84	3904.32	1763.09	1006.27	5080.74	-3.19	10.81	1.74E-10	9.47E-10
hsa-miR-340-5p:chr5:179442361:179442382	1290.96	311.57	242.86	254.79	190.07	44.12	41.35	24.44	13.42	36.45	10.91	4.01	7.81	2.59E-10	1.38E-09
hsa-miR-30e-5p:chr1:41220043:41220064	22972.89	13091.77	17389.24	14390.96	7041.65	2317.64	3449.98	2220.05	1994.86	1885.34	3397.95	2.56	13.00	3.74E-10	1.94E-09
hsa-let-7i-3p:chr12:62997527:62997548	10.39	14.49	18.53	17.46	23.33	115.34	148.99	262.46	121.67	71.80	422.86	-3.50	6.80	3.76E-10	1.94E-09
hsa-miR-30e-3p:chr1:41220085:41220106	1008.14	1135.47	1925.53	1313.02	505.86	12062.71	10613.66	7895.08	7309.57	4509.06	10342.82	-2.90	12.38	4.15E-10	2.10E-09

hsa-miR-582-3p:chr5:58999456:58999477	22.92	23.89	11.12	38.24	13.80	223.71	186.24	121.73	149.43	395.07	142.21	-3.21	6.92	5.41E-10	2.68E-09
hsa-miR-324-3p:chr17:7126627:7126646	10.31	4.11	5.43	4.74	5.10	31.74	38.37	40.73	31.00	56.13	31.60	-2.68	4.56	5.49E-10	2.68E-09
hsa-miR-24-3p:chr9:97848346:97848367	14.30	18.11	16.20	18.38	22.97	1.55	1.49	4.53	2.78	0.73	3.01	2.96	3.32	6.32E-10	3.03E-09
hsa-let-7g-3p:chr3:52302296:52302316	4.27	2.93	3.27	4.38	5.28	26.71	14.90	28.51	26.83	47.38	19.19	-2.75	4.06	8.07E-10	3.80E-09
hsa-miR-335-5p:chr7:130135967:130135989	190.69	98.91	57.14	83.89	115.64	17.03	17.51	7.69	5.09	18.95	14.67	3.01	5.85	9.87E-10	4.57E-09
hsa-miR-92b-3p:chr1:155165028:155165049	69.42	55.79	82.99	34.28	63.28	295.32	299.85	432.61	353.45	358.63	242.66	-2.43	7.70	1.04E-09	4.72E-09
hsa-miR-135b-5p:chr1:205417489:205417511	710.89	425.67	666.52	330.84	468.62	42.58	68.17	37.11	5.55	29.52	87.28	3.53	8.03	1.05E-09	4.72E-09
hsa-miR-361-5p:chrX:85158686:85158707	48.27	54.33	51.45	63.68	121.04	443.17	557.61	519.49	285.91	543.41	255.45	-2.68	8.06	1.68E-09	7.40E-09
hsa-miR-203a:chr14:104583806:104583827	133.96	402.06	287.33	218.32	245.31	2552.96	2747.84	3226.90	4427.38	5770.81	40490.20	-5.26	12.43	1.86E-09	8.08E-09
hsa-miR-374b-5p:chrX:73438422:73438443	416.62	243.10	225.02	206.80	383.09	51.48	75.61	64.71	39.32	56.13	68.47	2.31	7.38	2.74E-09	1.17E-08
hsa-miR-101-3p:chr1:65524125:65524145	3.83	11.84	10.94	14.21	6.12	0.39	1.49	1.36	0.46	0.00	0.38	3.78	2.37	4.64E-09	1.96E-08
hsa-miR-96-5p:chr7:129414579:129414601	691.07	353.78	349.98	295.64	127.58	39.09	14.15	10.41	11.57	76.17	18.81	3.68	7.50	7.00E-09	2.90E-08
hsa-miR-203b-3p:chr14:104583765:104583787	3.38	10.59	6.20	2.26	4.08	33.29	26.45	55.21	70.32	133.76	221.21	-4.08	5.68	8.12E-09	3.32E-08
hsa-miR-369-3p:chr14:101531978:101531998	13.58	12.12	45.07	34.56	70.66	2.32	5.21	5.43	4.16	2.92	1.88	3.27	4.21	9.72E-09	3.92E-08
hsa-miR-126-5p:chr9:139565068:139565088	966.32	1090.82	781.66	1340.94	722.69	229.91	297.99	284.63	202.63	169.11	147.10	2.14	9.15	1.06E-08	4.22E-08
hsa-miR-200c-5p:chr12:7072866:7072887	6.33	5.43	4.31	1.55	1.20	35.22	28.31	16.29	37.47	39.73	30.47	-3.05	4.22	1.14E-08	4.46E-08
hsa-miR-151a-5p:chr8:141742722:141742742	11.16	14.91	6.89	6.57	16.55	1.94	0.74	2.26	1.85	1.09	1.50	2.85	2.70	1.25E-08	4.81E-08
hsa-miR-19a-3p:chr13:92003193:92003215	102.94	62.06	49.55	69.12	61.48	17.03	10.06	2.72	6.48	15.67	9.03	2.76	5.23	2.17E-08	8.25E-08
hsa-miR-140-5p:chr16:69967006:69967027	246.77	256.68	235.45	358.75	237.46	49.93	67.05	88.69	34.23	28.79	23.33	2.46	7.21	3.14E-08	1.18E-07
hsa-miR-301a-3p:chr17:57228510:57228532	321.14	84.84	59.55	63.04	70.36	19.35	5.21	4.98	7.40	18.59	4.14	3.59	5.92	3.22E-08	1.19E-07
hsa-miR-501-3p:chrX:49774380:49774401	7.29	6.06	8.62	7.85	10.80	65.02	34.27	39.37	37.94	22.23	50.04	-2.35	4.72	3.65E-08	1.34E-07
hsa-miR-191-5p:chr3:49058105:49058127	1400.55	1366.03	1621.74	1579.90	1324.04	273.64	399.31	333.05	346.51	562.72	203.53	2.05	9.74	3.74E-08	1.35E-07
hsa-miR-29a-5p:chr7:130561545:130561566	29.25	17.90	20.34	32.30	25.55	6.97	4.47	5.88	4.63	8.02	5.64	2.07	3.92	9.07E-08	3.23E-07
hsa-miR-195-5p:chr17:6920986:6921006	354.86	272.36	211.14	157.82	269.91	20.90	54.01	62.90	43.49	7.65	38.37	2.74	7.09	1.20E-07	4.23E-07
hsa-miR-18a-5p:chr13:92003010:92003032	66.44	67.91	32.06	49.12	46.42	10.84	7.08	3.17	2.78	17.13	5.27	2.75	4.84	1.34E-07	4.67E-07
hsa-miR-135b-3p:chr1:205417451:205417472	22.44	13.23	24.82	9.33	12.30	3.48	2.23	2.26	0.00	1.46	3.01	2.96	3.19	1.69E-07	5.81E-07
hsa-miR-338-3p:chr17:79099687:79099708	49.15	34.41	97.47	51.59	9.30	444.33	325.93	199.56	264.62	899.48	717.44	-3.30	8.14	1.79E-07	6.07E-07
hsa-miR-183-5p:chr7:129414807:129414828	2316.79	2353.06	2057.30	1676.58	428.73	39218.31	5786.22	5229.29	10729.34	33965.19	22125.03	-3.47	13.48	3.15E-07	1.06E-06
hsa-miR-20a-5p:chr13:92003326:92003348	761.17	1119.59	697.46	1003.53	477.01	251.58	176.56	133.49	133.24	302.86	105.72	2.14	8.88	3.36E-07	1.11E-06

hsa-miR-34c-5p:chr11:111384176:111384198	4775.67	11688.05	3427.66	2498.55	429.15	322.02	8.19	3.17	6.94	12.03	12.79	6.23	11.04	7.52E-07	2.43E-06
hsa-miR-181b-5p:chr9:127456004:127456026	13.82	17.34	9.57	23.11	17.63	3.48	2.23	0.91	4.63	1.09	4.89	2.50	3.25	7.52E-07	2.43E-06
hsa-miR-424-5p:chrX:133680710:133680731	1481.61	237.39	453.31	266.24	1145.12	98.70	131.12	71.50	51.81	12.03	13.54	3.51	8.50	9.53E-07	3.05E-06
hsa-miR-28-5p:chr3:188406582:188406603	293.90	291.23	300.51	266.52	360.59	44.12	71.52	81.45	102.70	30.98	110.23	2.04	7.48	1.03E-06	3.25E-06
hsa-miR-139-3p:chr11:72326110:72326132	2.30	6.27	8.88	6.50	1.26	30.96	48.05	36.20	17.58	23.33	39.88	-2.70	4.33	1.19E-06	3.71E-06
hsa-miR-197-3p:chr1:110141562:110141583	36.82	60.25	64.38	69.90	125.60	313.51	409.74	837.61	210.50	313.07	234.76	-2.44	7.93	1.41E-06	4.32E-06
hsa-miR-374b-3p:chrX:73438392:73438413	11.08	10.45	7.07	10.95	8.34	2.32	2.98	1.36	1.85	3.28	1.13	2.13	2.59	1.45E-06	4.39E-06
hsa-miR-146b-3p:chr10:104196313:104196334	2.42	4.11	14.91	7.56	9.96	53.80	71.89	200.92	22.67	68.88	32.35	-3.27	5.47	1.95E-06	5.82E-06
hsa-miR-376c-3p:chr14:101506069:101506089	15.43	10.45	25.25	16.18	63.34	1.94	4.47	7.69	4.16	1.46	3.01	2.79	3.86	2.35E-06	6.95E-06
hsa-miR-216a-5p:chr2:56216155:56216176	8.70	5.22	7.07	7.21	7.50	0.39	0.74	0.91	1.85	0.00	2.63	2.72	2.12	2.64E-06	7.73E-06
hsa-miR-1266-5p:chr15:52569363:52569385	2.90	6.55	3.71	1.98	3.42	13.93	21.23	4.07	119.82	42.64	96.69	-3.74	4.84	3.07E-06	8.88E-06
hsa-miR-30a-3p:chr6:72113257:72113278	327.34	765.66	614.64	719.48	111.32	8446.14	5709.49	3675.80	3134.32	485.46	9354.51	-3.34	11.57	3.24E-06	9.18E-06
hsa-miR-655-3p:chr14:101515947:101515968	3.26	4.39	8.70	7.63	19.49	0.77	2.23	0.91	2.31	0.73	0.38	2.83	2.36	6.80E-06	1.89E-05
hsa-miR-411-5p:chr14:101489677:101489697	29.29	9.96	51.02	33.71	34.85	1.94	11.92	7.24	6.94	3.64	3.01	2.46	4.18	7.73E-06	2.13E-05
hsa-miR-451a:chr17:27188421:27188442	1489.34	8304.70	5176.86	562.30	11625.31	609.60	1131.24	329.89	244.27	690.28	898.02	3.06	11.46	7.95E-06	2.17E-05
hsa-miR-150-5p:chr19:50004089:50004110	70.67	194.13	243.55	329.07	103.04	4013.29	2669.25	1085.14	1381.88	237.99	588.02	-3.14	9.95	9.51E-06	2.57E-05
hsa-miR-3934-5p:chr6:33665929:33665950	11.52	9.33	10.77	13.85	7.14	0.77	1.12	0.00	0.93	0.00	4.51	3.07	2.58	1.04E-05	2.75E-05
hsa-miR-184:chr15:79502182:79502203	38.88	6.90	695.99	38.38	4.62	8.90	9.31	2.72	2.31	0.36	3.76	5.10	6.22	1.71E-05	4.48E-05
hsa-miR-194-5p:chr11:64658876:64658897	13.78	108.59	8.62	6.86	3.54	0.00	1.12	0.91	3.70	1.82	2.63	4.05	3.85	1.87E-05	4.82E-05
hsa-let-7b-3p:chr22:46509625:46509646	5.68	11.91	15.43	7.00	15.83	36.00	67.42	150.69	37.01	24.05	50.04	-2.44	5.26	1.92E-05	4.90E-05
hsa-miR-141-3p:chr12:7073318:7073339	5396.72	3180.30	3923.11	2318.11	630.80	329.76	740.13	222.64	399.25	546.32	1236.61	2.42	10.75	2.15E-05	5.39E-05
hsa-miR-215-5p:chr1:220291258:220291278	6.04	158.26	13.01	12.37	18.05	1.55	2.61	4.53	5.09	2.19	0.00	3.97	4.39	2.19E-05	5.44E-05
hsa-miR-136-5p:chr14:101351053:101351075	48.71	17.97	47.66	30.96	80.07	6.19	14.53	5.88	18.97	4.74	3.76	2.33	4.70	2.46E-05	6.06E-05
hsa-miR-542-5p:chrX:133675430:133675452	55.15	4.11	7.24	11.45	22.61	3.48	2.61	3.62	0.93	0.00	0.38	3.45	3.43	3.12E-05	7.60E-05
hsa-let-7b-5p:chr22:46509571:46509592	1939.73	3949.65	4022.04	4093.50	7393.43	8273.52	12898.50	15650.78	35439.38	10111.86	39969.15	-2.25	13.67	3.51E-05	8.42E-05
hsa-miR-93-5p:chr7:99691438:99691460	1086.98	1383.44	731.24	1140.64	754.42	5907.11	2915.09	2568.49	2630.52	11581.71	2085.34	-2.18	11.54	3.71E-05	8.82E-05
hsa-miR-429:chr1:1104435:1104456	3167.72	1752.34	834.23	336.77	200.39	73.54	170.60	54.75	44.41	330.93	244.91	3.04	9.36	4.02E-05	9.48E-05
hsa-miR-598-3p:chr8:10892731:10892752	63.41	67.71	68.43	139.16	51.82	17.03	22.35	33.49	18.04	3.28	10.16	2.17	5.51	5.46E-05	1.25E-04
hsa-miR-502-3p:chrX:49779257:49779278	12.85	9.89	15.17	15.83	18.95	1.55	1.49	3.62	3.70	0.36	7.52	2.26	3.13	6.39E-05	1.46E-04

hsa-miR-182-5p:chr7:129410287:129410310	6218.28	7988.81	8526.53	6177.46	1683.37	55864.03	11689.03	7092.31	22638.84	43731.55	78495.53	-2.58	14.47	8.04E-05	1.82E-04
hsa-miR-130b-3p:chr22:22007643:22007664	203.82	156.31	174.52	124.25	393.40	28.64	30.17	47.06	87.90	79.82	10.91	2.15	6.93	8.13E-05	1.82E-04
hsa-miR-142-3p:chr17:56408606:56408628	5288.55	3653.47	4956.07	13301.06	2872.45	3015.48	1327.55	1100.07	1353.20	727.46	337.84	2.20	11.75	1.00E-04	2.20E-04
hsa-miR-508-3p:chrX:146318463:146318485	2.42	1.67	5.43	2.69	1.86	3.87	32.41	303.64	4.16	0.73	104.96	-4.73	5.39	1.42E-04	3.07E-04
hsa-miR-200a-3p:chr1:1103296:1103317	4736.59	3326.51	2012.75	595.80	373.61	179.59	401.91	79.19	113.34	546.69	719.69	2.70	10.22	1.76E-04	3.75E-04
hsa-miR-134-5p:chr14:101521031:101521052	2.46	10.31	21.63	24.81	59.02	54.19	200.77	274.68	250.28	45.92	133.56	-2.76	6.61	2.42E-04	5.06E-04
hsa-miR-10a-5p:chr17:46657266:46657288	5560.49	25478.96	7328.79	7489.92	5058.63	71176.77	48054.23	47511.19	38152.71	5886.34	119629.81	-2.44	15.08	3.23E-04	6.61E-04
hsa-miR-132-3p:chr17:1953223:1953244	27.36	73.49	104.62	96.90	55.54	186.56	308.79	848.02	300.25	161.82	92.92	-2.14	7.68	3.49E-04	7.09E-04
hsa-miR-590-3p:chr7:73605583:73605603	57.85	23.33	18.27	31.95	29.09	4.64	1.86	0.45	8.79	17.13	5.27	2.33	4.22	3.66E-04	7.37E-04
hsa-miR-493-3p:chr14:101335453:101335474	3.22	3.76	16.55	11.17	15.77	0.39	2.98	5.43	0.93	0.36	1.13	2.44	2.61	4.94E-04	9.74E-04
hsa-miR-10b-5p:chr2:177015057:177015079	861.73	2667.84	2197.09	868.12	1839.62	11998.46	7068.32	14739.86	2481.55	447.19	44309.89	-3.00	12.99	5.06E-04	9.90E-04
hsa-miR-10a-3p:chr17:46657226:46657247	20.99	49.80	12.67	9.26	10.68	6.19	3.35	7.69	2.31	0.73	7.90	2.14	3.64	5.98E-04	1.16E-03
hsa-miR-452-5p:chrX:151128150:151128171	1545.39	425.53	360.58	827.20	338.94	45.28	280.86	205.44	217.90	14.58	122.27	2.24	8.64	8.05E-04	1.51E-03
hsa-miR-452-3p:chrX:151128106:151128127	39.04	6.48	4.91	14.28	12.06	1.55	4.84	2.26	6.48	0.00	2.63	2.38	3.19	1.16E-03	2.12E-03
hsa-miR-3065-3p:chr17:79099726:79099748	63.41	56.35	105.31	67.71	6.24	95.21	86.42	208.16	119.82	595.16	999.60	-2.55	7.77	1.33E-03	2.41E-03
hsa-miR-92a-3p:chr13:92003615:92003636	17.61	47.44	37.06	37.25	28.91	107.21	49.17	22.17	341.88	33.53	799.83	-2.74	7.11	1.38E-03	2.49E-03
hsa-miR-217:chr2:56210155:56210177	4.92	9.26	9.31	10.46	9.78	20.13	23.47	22.17	30.07	5.47	117.38	-2.06	4.58	1.82E-03	3.23E-03
hsa-miR-379-5p:chr14:101488408:101488428	24.13	48.97	170.98	184.68	285.86	229.91	715.92	755.70	1111.24	246.01	445.44	-2.03	8.58	1.87E-03	3.31E-03
hsa-miR-339-5p:chr7:1062626:1062648	6.04	9.33	11.46	11.10	14.45	0.77	0.37	0.00	3.24	1.82	7.52	2.18	2.70	1.89E-03	3.32E-03
hsa-miR-205-5p:chr1:209605511:209605532	1046.33	475.96	172.10	18.09	1715.22	318.93	12.29	33.03	9.25	4.01	33.86	3.32	8.45	2.47E-03	4.26E-03
hsa-miR-144-5p:chr17:27188601:27188622	342.01	1847.84	968.76	157.61	1209.96	259.71	558.36	73.31	55.98	231.43	124.15	2.06	9.05	2.53E-03	4.31E-03
hsa-miR-192-5p:chr11:64658675:64658695	455.42	8501.34	470.98	378.19	80.91	212.10	416.44	240.74	264.16	629.78	270.50	2.54	10.08	4.47E-03	7.36E-03
