# Supplementary material for: snoRNA and piRNA expression levels modified by tobacco use in women with lung adenocarcinoma
Source: PLoS One. 2017 Aug 17;12(8):e0183410. doi: 10.1371/journal.pone.0183410 (PMC5560661; doi:10.1371/journal.pone.0183410)
Supplement: S8 File — (PDF) [file pone.0183410.s008.pdf]

Supplemental File 8

<u>snoRNA and piRNA analysis</u>

<u>Normal Non-Smoker x Normal Smoker</u>

for the manuscript: “snoRNA and piRNA expression levels modified by tobacco use in women with lung adenocarcinoma” by

Natasha Andressa Nogueira Jorge, Gabriel Wajnberg, Carlos Gil Ferreira, Benilton de Sa Carvalho, Fabio Passetti

The CPM counts were calculated using the EdgeR Bioconductor package and normalized using the TMM methodology. Figure 1 shows the total raw and normalized counts.

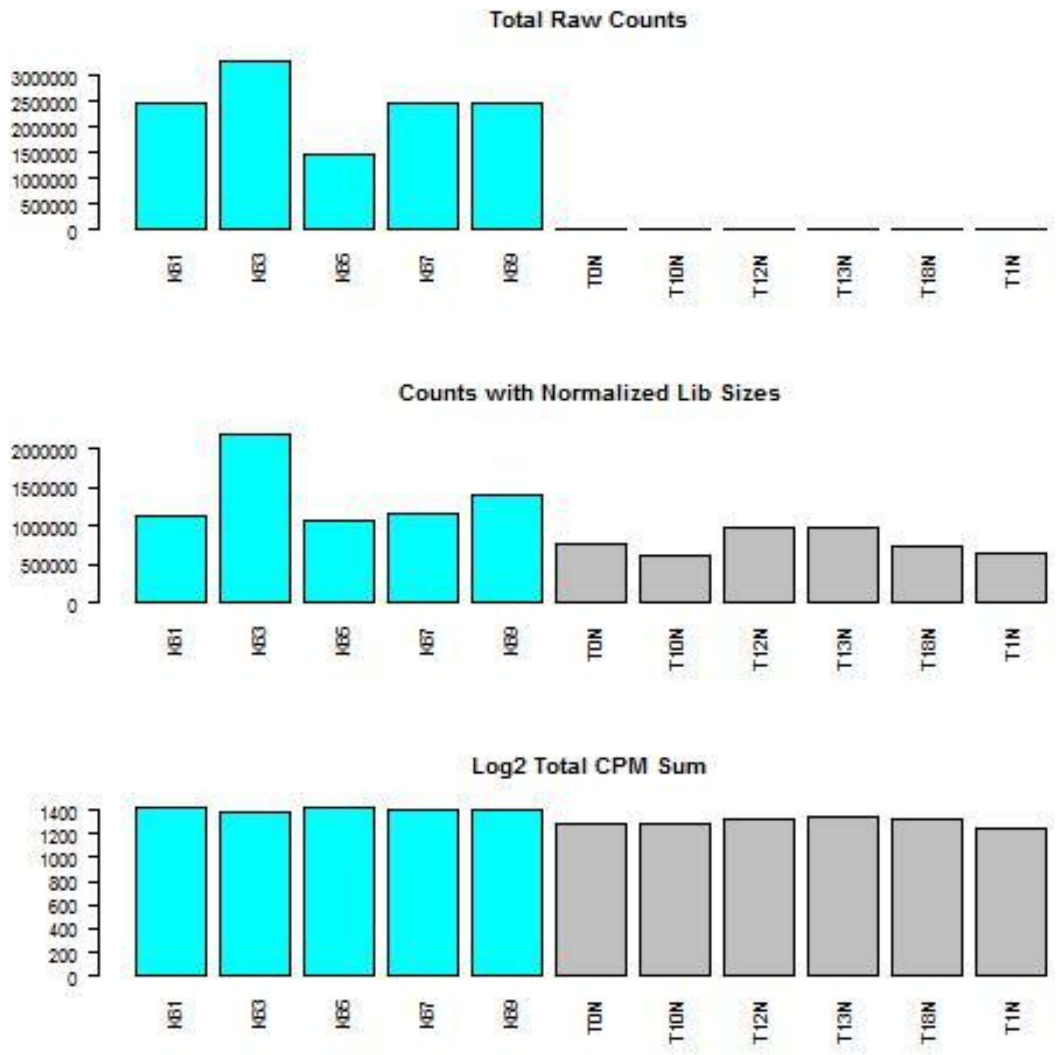

Figure 1. Raw, Normalized and log2 Normalized Total Counts. Blue bars indicate Non-smokers samples and gray bars indicate smokers samples.

Hierarchical clustering was performed on the normalized CPM counts (Figure 2). The non-smokers and smokers were completely separated. Only one non-smoker sample, K63, was clusterized separated from all other samples.

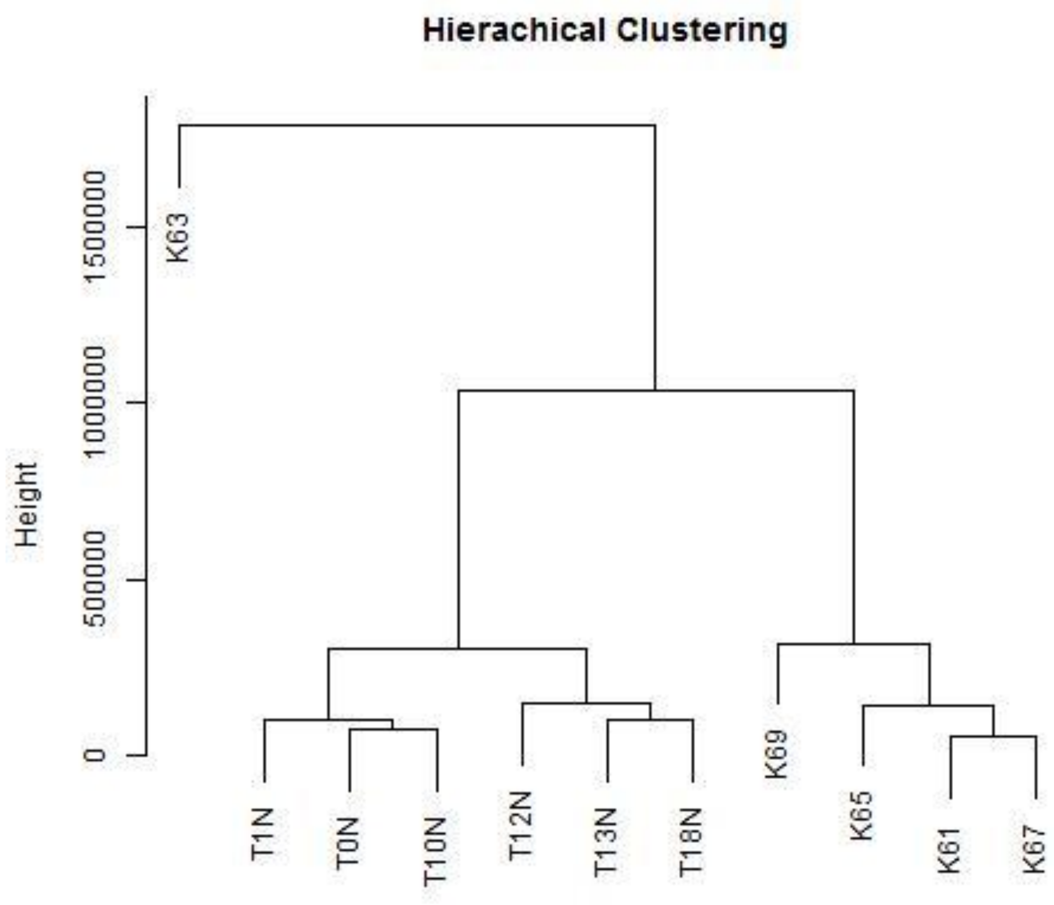

Figure 2. Hierarchical cluster for normalized counts. Samples starting with ‘K’ are from Non-smokers and samples starting with ‘T’ are from smokers.

In order to further investigate the distribution of our samples, we used the normalized counts to perform principal component analysis. This analysis revealed two clearly distinct groups that correspond to non-smokers and smokers (Figure 3). The non-smokers samples present the same behavior pattern, while the smokers samples are more dispersed.

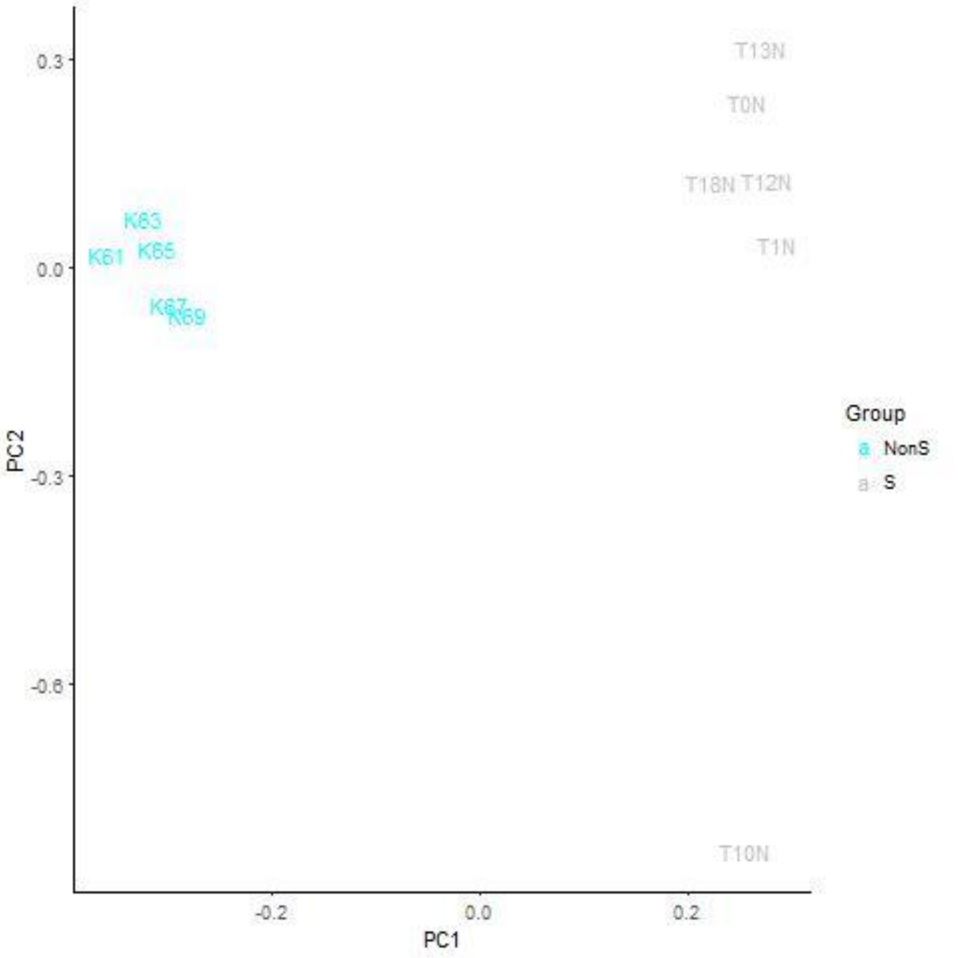

Figure 3. PCA analysis.

After applying our differential expression filters, FDR < 0.01 and logFC > 2 or logFC < -2, we found 49 differentially expressed snoRNA (Figure 4). The majority of snoRNAs is, 29 snoRNAs, are up-regulated in non-smokers, while 20 snoRNAs are down-regulated (Figure 5). Table 1 shows the normalized CPM counts in each sample evaluated, the logFC, logCPM, p-value and FDR.

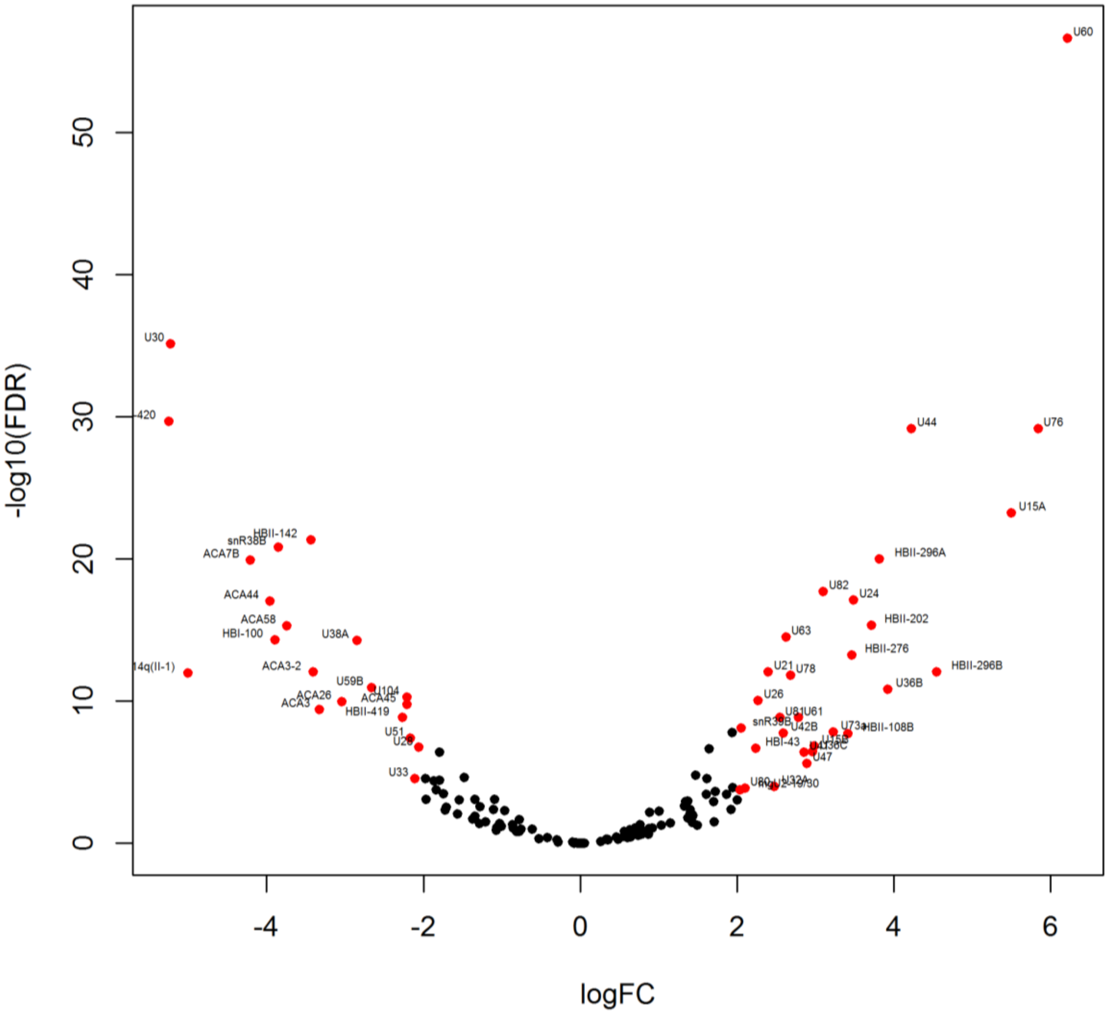

Figure 4. Volcano Plot. The red dots indicate the differentially expressed genes found. The genes on the left side of the plot are up-regulated in non-smokers and the ones one the right side are down-regulated.

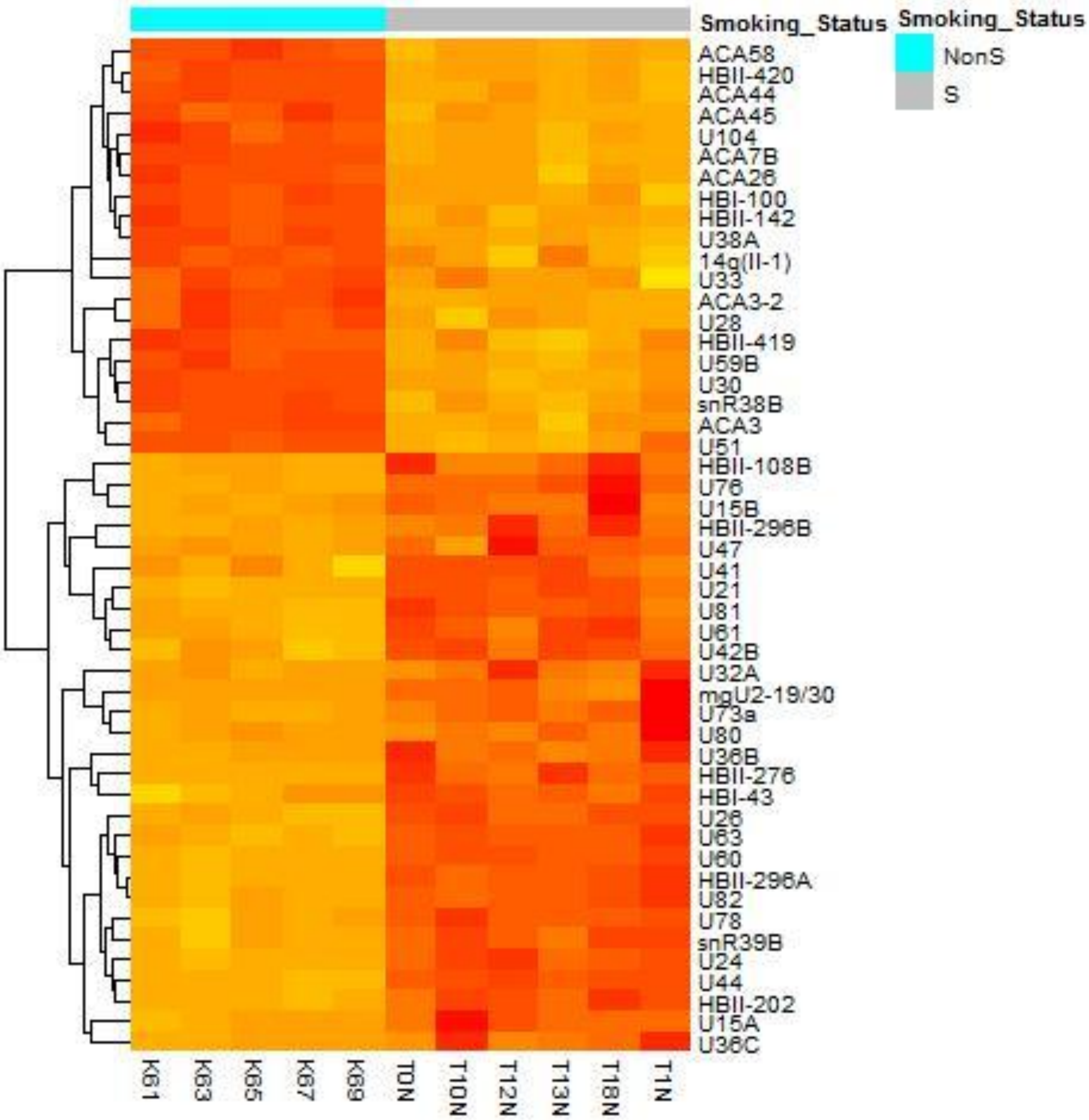

Figure 5. Heatmap. The samples starting with 'K' are from Non-smokers and the ones starting with 'T' are from smokers. A total of 29 snoRNAs were found up-regulated in non-smokers (yellow area on bottom left of the heatmap) and 20 up-regulated in smokers (yellow area on top right).

Table 1. Differentially expressed snoRNAs

Gene	K61	K63	K65	K67	K69	T0N	T10N	T12N	T13N	T18N	T1N	logFC	logCPM	PValue	FDR
U60	782588.47	1751491.46	643885.83	740903.64	961957.52	14115.21	10499.46	13575.76	15010.69	18041.40	7180.28	6.22	18.78	1.57E-59	2.31E-57
U30	4563.00	5173.93	6496.29	6356.90	6104.03	145027.97	142326.08	387509.14	268303.58	253362.17	97019.28	-5.23	16.87	9.73E-38	7.16E-36
HBII-420	1282.35	449.67	647.65	661.49	648.21	27825.47	22165.54	17438.47	27088.82	15433.01	58468.01	-5.25	13.92	4.11E-32	2.01E-30
U44	7641.76	8126.84	9757.99	11200.34	10093.17	694.19	453.68	337.52	695.86	347.79	512.88	4.23	12.19	2.20E-31	6.63E-30
U76	9814.37	11249.88	4639.06	6240.55	6966.23	231.40	194.43	150.01	49.70	0.00	256.44	5.84	11.88	2.25E-31	6.63E-30
U15A	8490.90	8062.13	3389.91	2983.28	3326.17	231.40	0.00	37.50	149.11	130.42	170.96	5.50	11.34	2.27E-25	5.55E-24
HBII-142	3346.54	6401.53	8422.39	6365.81	6968.50	66121.65	43294.09	90417.55	62627.36	59645.31	87616.53	-3.44	15.29	2.16E-23	4.53E-22
snR38B	4026.51	5385.43	5584.89	4229.80	6270.34	99327.09	43164.47	85429.77	126248.81	58341.12	29148.52	-3.85	15.37	8.26E-23	1.52E-21
HBII-296A	3879.76	4953.10	3155.47	4025.72	3466.94	231.40	388.87	337.52	347.93	217.37	85.48	3.82	11.00	6.09E-22	9.94E-21
ACA7B	58.42	68.05	115.02	109.78	98.20	1851.17	1166.61	1050.06	2634.33	1521.56	1795.07	-4.21	9.70	8.20E-22	1.21E-20
U82	10857.92	13595.00	8100.03	9883.92	10086.36	1272.68	1685.10	1275.07	1540.83	999.89	512.88	3.10	12.44	1.40E-19	1.87E-18
U24	16056.95	23461.16	12191.81	13608.45	14059.60	2603.21	777.74	487.53	2037.87	1565.04	1025.75	3.49	12.99	6.12E-19	7.50E-18
ACA44	74.31	38.70	70.33	55.36	66.41	983.44	972.17	525.03	1192.90	782.52	1367.67	-3.96	8.77	8.07E-19	9.12E-18
HBII-202	2901.18	3608.74	2728.34	3988.19	3837.58	636.34	129.62	150.01	397.63	86.95	170.96	3.72	10.82	4.31E-17	4.52E-16
ACA58	87.86	86.07	40.30	102.74	144.74	1851.17	972.17	1012.56	1192.90	956.41	1538.63	-3.75	9.25	4.95E-17	4.85E-16
U63	15353.62	22542.46	26543.43	22125.76	27738.33	4338.69	3305.39	4200.23	4622.50	3825.65	1795.07	2.63	13.61	3.44E-16	3.16E-15
HBI-100	137.39	207.49	411.01	157.16	283.24	2313.97	2333.21	2587.64	3330.19	1999.77	8975.35	-3.89	10.90	5.68E-16	4.92E-15
U38A	532.29	522.40	825.68	539.52	651.04	3702.35	3435.01	4650.26	4026.04	4260.38	6581.92	-2.85	11.34	6.38E-16	5.21E-15
HBII-276	2953.52	2728.07	2805.27	3170.00	2878.90	57.85	259.25	600.03	49.70	347.79	170.96	3.47	10.62	7.47E-15	5.78E-14
U21	18703.44	29080.10	19602.43	19770.18	21695.60	3355.25	3888.69	4500.25	2584.62	3521.33	7094.80	2.40	13.58	1.15E-13	8.37E-13
HBII-296B	1540.78	1272.97	833.01	981.45	667.50	115.70	64.81	0.00	49.70	0.00	85.48	4.55	9.25	1.20E-13	8.37E-13
ACA3-2	141.13	38.70	63.01	79.29	36.33	809.89	842.55	675.04	646.16	782.52	854.80	-3.41	8.45	1.35E-13	9.00E-13
14q(II-1)	51.41	214.16	91.58	153.41	112.95	694.19	1685.10	8437.97	497.04	2999.66	9830.15	-5.01	11.04	1.57E-13	1.01E-12
U78	6042.56	7989.40	3205.29	5349.65	4024.33	925.59	388.87	1012.56	994.09	825.99	769.32	2.68	11.54	2.43E-13	1.49E-12
U59B	427.14	248.19	475.48	456.48	397.89	2545.37	2009.16	2662.65	3976.34	2304.08	1624.11	-2.66	10.53	1.84E-12	1.08E-11
U36B	761.28	1185.57	654.24	723.42	638.56	0.00	64.81	37.50	99.41	86.95	0.00	3.93	8.90	2.57E-12	1.45E-11

U104	13998.84	23766.06	40352.90	28543.64	33484.78	138780.25	106809.37	108381.05	170982.65	104553.19	148221.53	-2.21	16.35	9.56E-12	5.20E-11
U26	5026.12	4269.91	5829.59	6826.51	6799.35	983.44	907.36	1500.08	1789.35	956.41	940.27	2.27	11.71	1.75E-11	9.19E-11
ACA26	41.12	67.38	68.14	59.11	85.14	404.94	388.87	412.52	894.68	478.21	598.36	-3.04	7.84	2.06E-11	1.04E-10
ACA45	1441.71	2683.37	2177.40	1276.54	1660.25	10644.25	5573.79	7162.90	9791.74	9303.28	8975.35	-2.21	12.41	3.52E-11	1.72E-10
ACA3	70.10	40.03	41.03	29.09	28.95	404.94	453.68	300.02	894.68	217.37	256.44	-3.33	7.33	8.14E-11	3.86E-10
U61	6554.29	6616.36	8235.57	12710.98	13425.58	578.49	1101.80	2850.16	646.16	434.73	2735.35	2.78	12.34	3.21E-10	1.43E-09
U81	2994.18	4290.59	3448.52	4655.78	4971.09	289.25	453.68	750.04	646.16	478.21	1709.59	2.55	11.19	3.22E-10	1.43E-09
HBII-419	2586.67	3337.87	4499.12	4899.27	4836.00	22040.55	9462.48	23963.84	31661.61	18215.30	11026.86	-2.27	13.59	3.31E-10	1.43E-09
snR39B	17798.22	26530.16	13624.11	17471.38	18903.55	5727.07	2722.08	4950.28	7952.68	3130.07	2649.87	2.05	13.44	1.85E-09	7.76E-09
U73a	879.05	501.05	808.10	699.02	424.57	173.55	64.81	37.50	99.41	43.47	0.00	3.23	8.71	3.59E-09	1.46E-08
U42B	1485.64	823.29	1002.25	1988.23	1469.53	173.55	129.62	375.02	149.11	173.89	341.92	2.59	9.69	4.50E-09	1.74E-08
HBII-108B	956.15	428.99	475.48	1048.54	792.95	0.00	129.62	150.01	49.70	0.00	85.48	3.41	8.84	5.18E-09	1.95E-08
U51	629.96	594.45	698.20	632.87	573.28	2776.76	3499.82	3037.67	4026.04	2477.98	940.27	-2.17	10.76	1.11E-08	4.06E-08
U15B	1290.30	665.84	971.48	758.14	532.98	57.85	64.81	150.01	149.11	0.00	256.44	2.98	9.04	3.93E-08	1.41E-07
U28	1154.30	443.00	663.77	937.35	597.12	2718.91	5314.54	2062.62	2684.03	3130.07	3162.74	-2.06	10.96	5.07E-08	1.78E-07
HBI-43	5935.08	3497.99	2773.76	1704.40	1711.33	404.94	518.49	825.05	596.45	1173.78	341.92	2.24	10.87	6.04E-08	2.06E-07
U36C	681.37	964.07	474.02	438.65	465.44	231.40	0.00	112.51	49.70	43.47	0.00	2.96	8.61	1.10E-07	3.58E-07
U41	799.13	1246.28	567.06	1532.22	4146.36	173.55	129.62	150.01	99.41	304.31	598.36	2.85	9.93	1.22E-07	3.90E-07
U47	908.49	513.06	896.01	1042.44	841.76	57.85	518.49	0.00	49.70	43.47	85.48	2.89	9.04	7.77E-07	2.38E-06
U33	205.16	100.74	174.37	112.59	101.60	462.79	259.25	525.03	497.04	391.26	1624.11	-2.11	8.30	9.90E-06	2.80E-05
U32A	419.19	160.79	695.27	481.81	461.46	231.40	64.81	0.00	49.70	130.42	0.00	2.47	8.26	3.69E-05	9.69E-05
U80	758.94	518.39	291.59	463.04	380.30	231.40	129.62	150.01	49.70	86.95	0.00	2.10	8.40	5.15E-05	1.31E-04
mgU2-19/30	463.59	500.38	358.26	420.35	376.32	57.85	64.81	37.50	149.11	260.84	0.00	2.04	8.24	7.16E-05	1.75E-04
