# Supplementary material for: snoRNA and piRNA expression levels modified by tobacco use in women with lung adenocarcinoma
Source: PLoS One. 2017 Aug 17;12(8):e0183410. doi: 10.1371/journal.pone.0183410 (PMC5560661; doi:10.1371/journal.pone.0183410)
Supplement: S9 File — (PDF) [file pone.0183410.s009.pdf]

# Supplemental File 9

## snoRNA and piRNA analysis

## Tumor Non-Smoker x Tumor Smoker

## for the manuscript: "snoRNA and piRNA expression levels modified by tobacco use in women with lung adenocarcinoma" by

Natasha Andressa Nogueira Jorge, Gabriel Wajnberg, Carlos Gil Ferreira, Benilton de Sa Carvalho, Fabio Passetti

The CPM counts were calculated using the EdgeR Bioconductor package and normalized using the TMM methodology. Figure 1 shows the total raw and normalized counts.

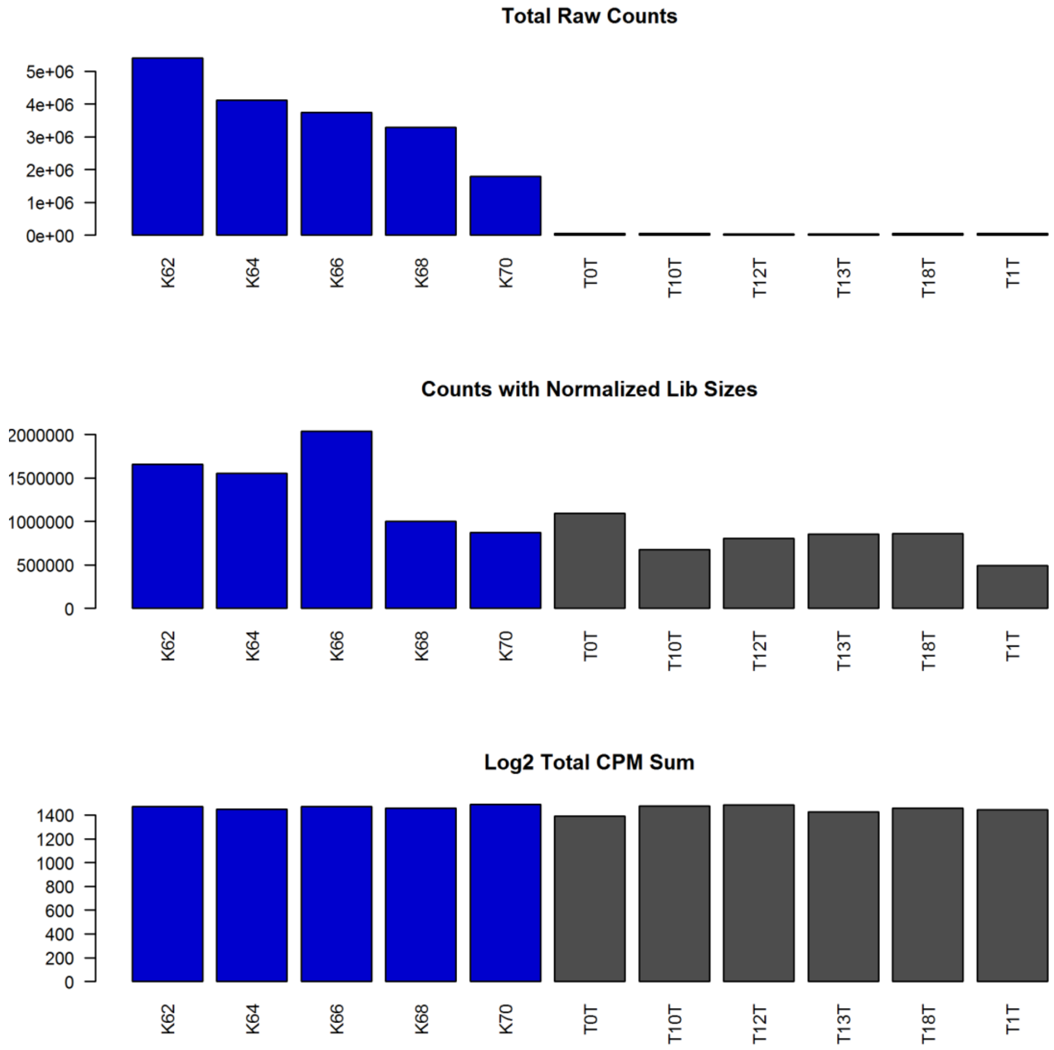


Figure 1. Raw, Normalized and log2 Normalized Total Counts. Blue bars indicate Non-smokers samples and gray bars indicate smokers samples.

Hierarchical clustering was performed on the normalized CPM counts (Figure 2). The samples show some variability because two samples of each group, non-smokers and smokers, were separated from the rest of the same group. However, there is still a distinction between non-smokers and smokers.

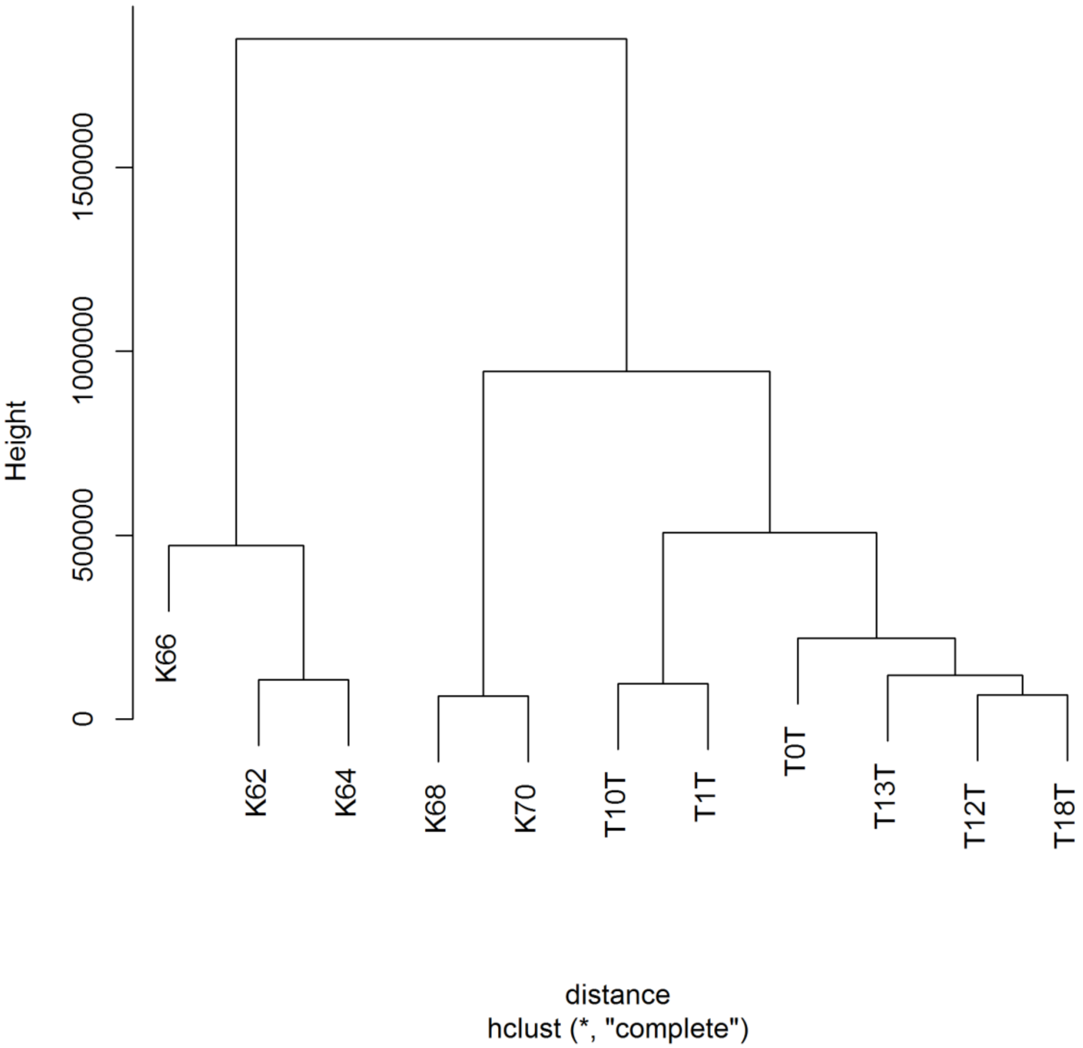


Figure 2. Hierarchical cluster for normalized counts. Samples starting with ‘K’ are from Non-smokers and samples starting with ‘T’ are from smokers.

In order to further investigate the distribution of our samples, we used the normalized counts to perform principal component analysis. This analysis revealed two clearly distinct groups that correspond to non-smokers and smokers (Figure 3). The non-smokers samples present the same behavior pattern, while the smokers samples are more dispersed.

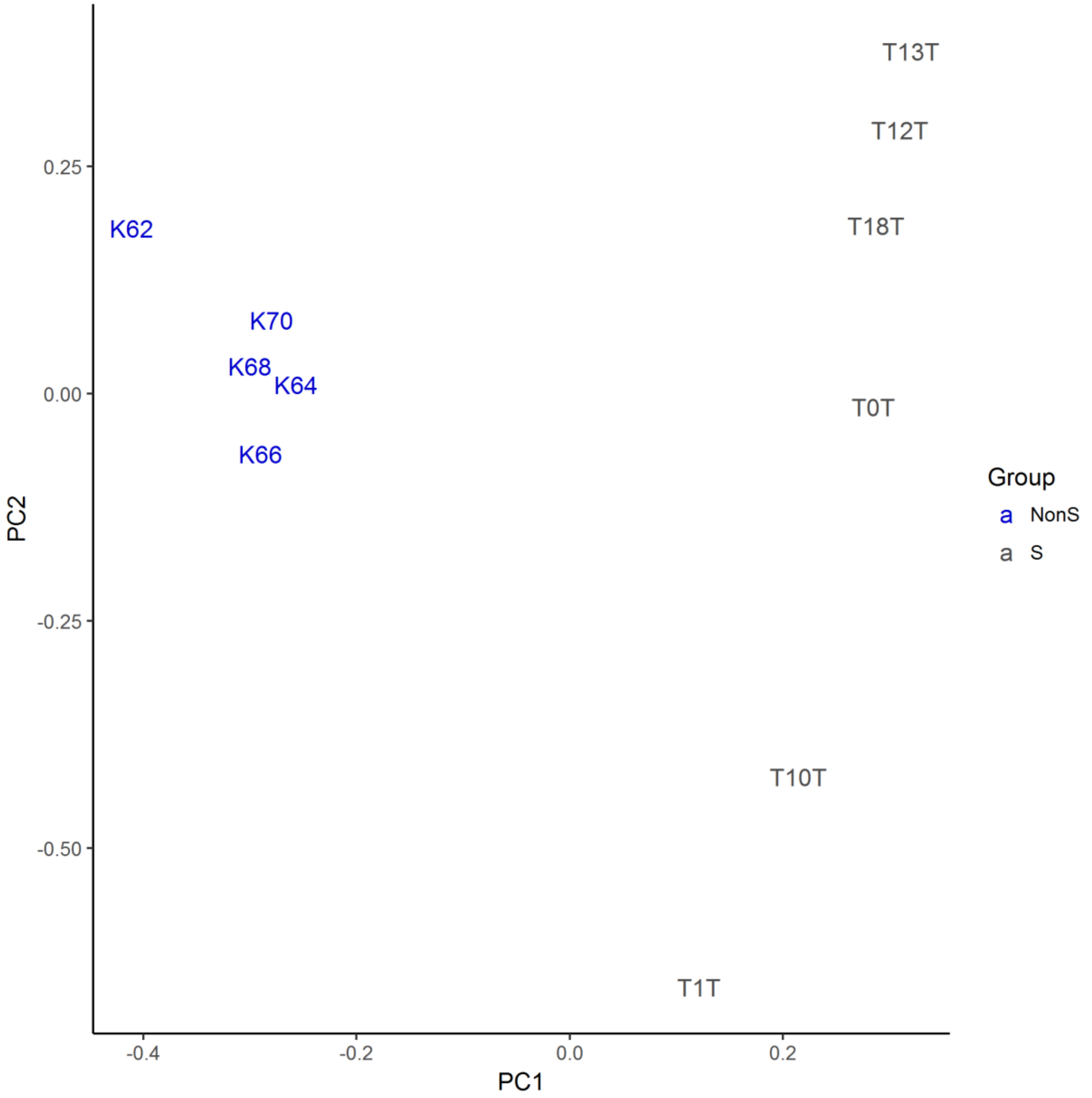


Figure 3. PCA analysis.

After applying our differential expression filters, FDR < 0.01 and logFC > 2 or logFC < -2, we found 55 differentially expressed sncRNA (Figure 4). The majority of sncRNAs, 34 sncRNAs, are up-regulated in non-smokers, while 21 snoRNAs are down-regulated (Figure 5). Table 1 shows the normalized CPM counts in each sample evaluated, the logFC, logCPM, p-value and FDR.

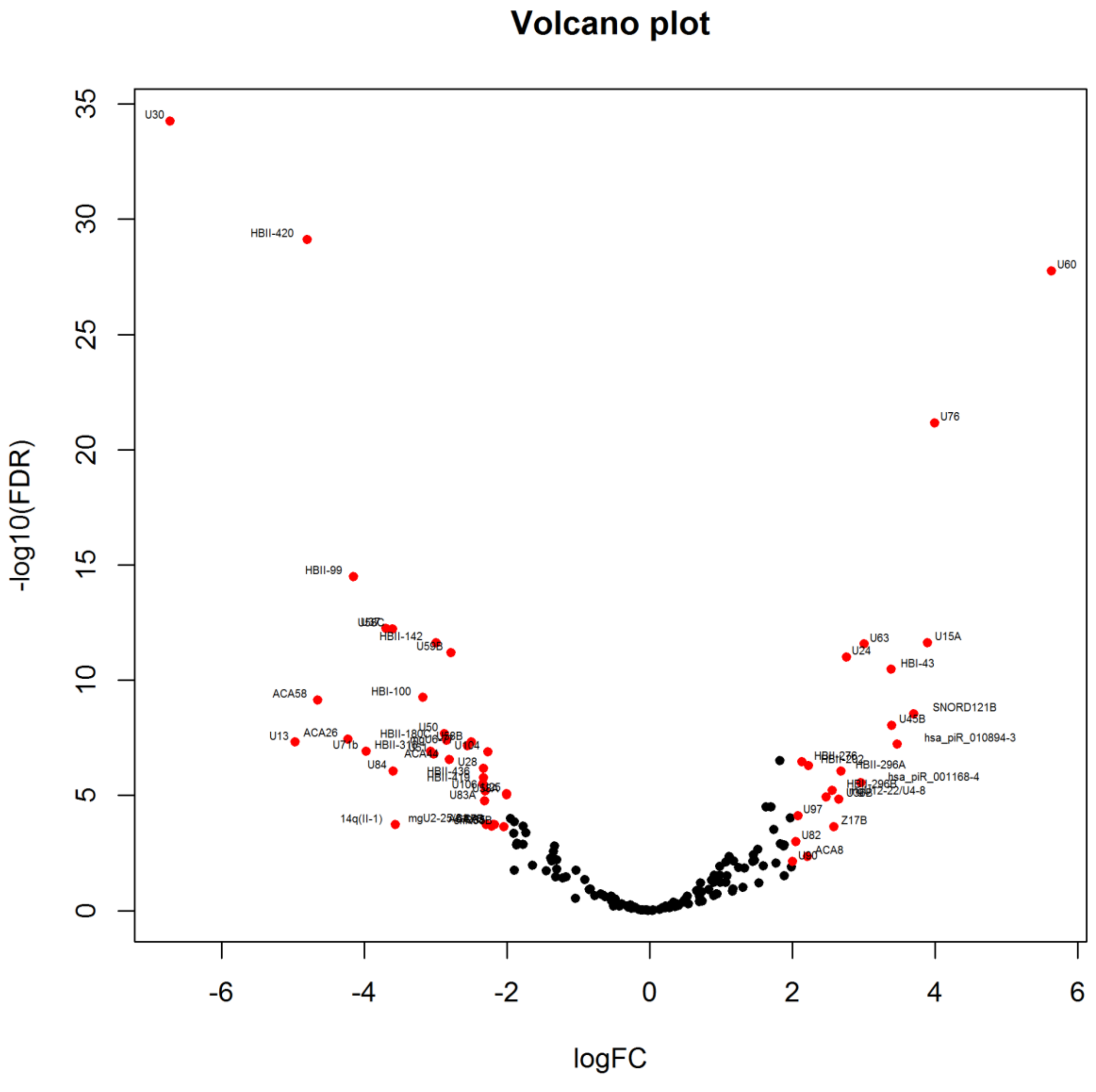


Figure 4. Volcano Plot. The red dots indicate the differentially expressed genes found. The genes on the left side of the plot are up-regulated in non-smokers and the ones one the right side are down-regulated.

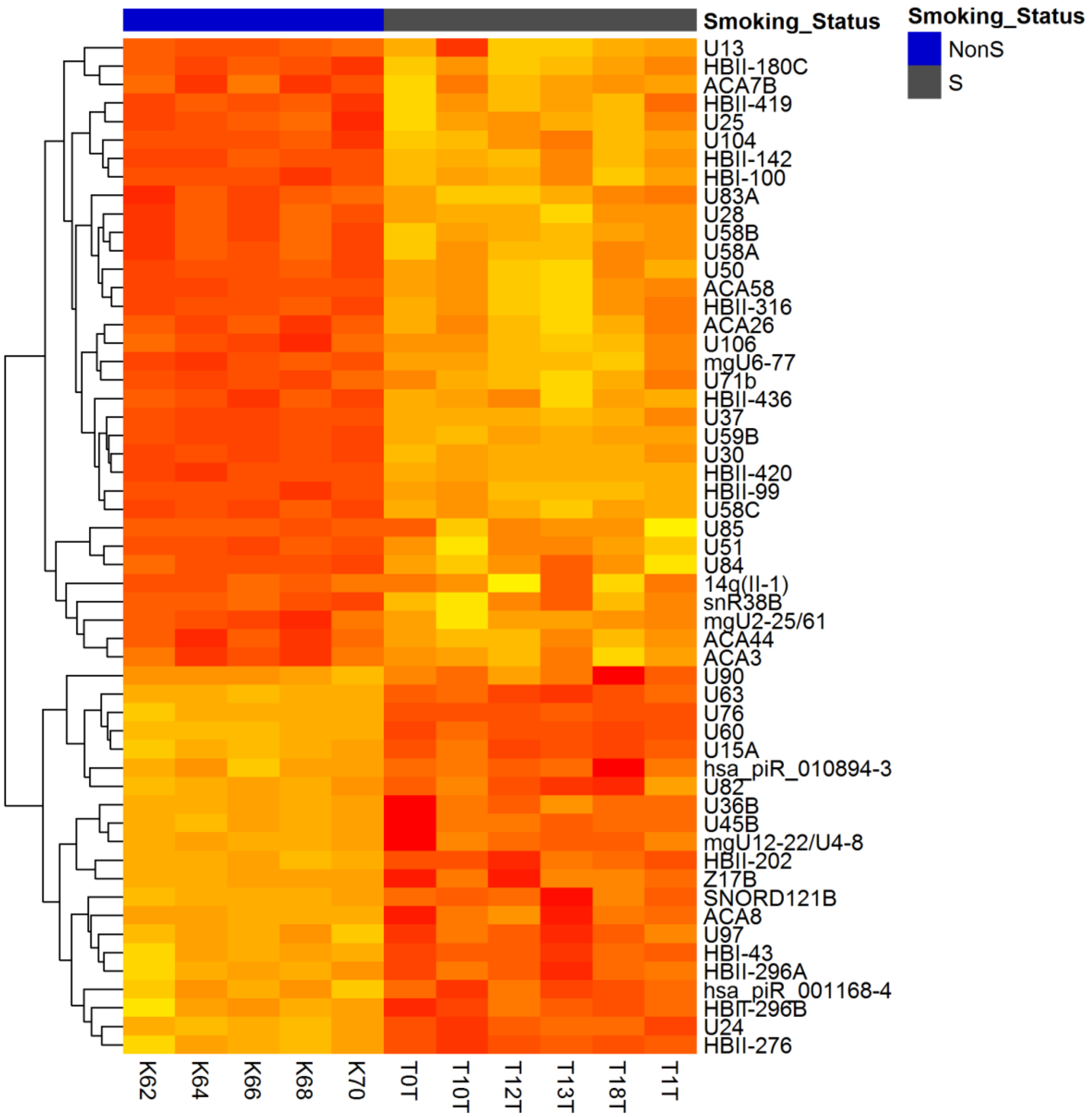


Figure 5. Heatmap. The samples starting with 'K' are from Non-smokers and the ones starting with 'T' are from smokers. A total of 34 snoRNAs were found up-regulated in smokers (yellow area on top right of the heatmap) and 21 up-regulated in smokers (yellow area on bottom left).

Table 1. Differentially expressed snoRNAs and piRNAs.

| Gene | K62 | K64 | K66 | K68 | K70 | T0T | T10T | T12T | T13T | T18T | T1T | logFC | logCPM | PValue | FDR |
|---|---|---|---|---|---|---|---|---|---|---|---|---|---|---|---|
| U30 | 3212.44 | 4628.22 | 3651.12 | 5559.13 | 2749.54 | 649423.86 | 227655.27 | 440893.34 | 552802.19 | 486680.09 | 147937.56 | -6.72 | 17.81 | 3.22E-37 | 5.38E-35 |
| HBII-420 | 586.06 | 324.28 | 651.24 | 667.08 | 778.51 | 19334.48 | 11472.18 | 18097.53 | 16094.53 | 20878.04 | 14834.88 | -4.80 | 13.20 | 8.90E-32 | 7.43E-30 |
| U60 | 1374383.79 | 1270126.57 | 1742409.52 | 696181.90 | 641741.89 | 11057.66 | 49190.05 | 19873.68 | 21536.12 | 13119.92 | 23450.95 | 5.63 | 19.02 | 3.04E-30 | 1.69E-28 |
| U76 | 9133.01 | 4647.45 | 4615.44 | 3690.63 | 4276.96 | 372.24 | 371.40 | 288.02 | 397.65 | 269.59 | 290.88 | 4.00 | 11.35 | 1.62E-23 | 6.78E-22 |
| HBII-99 | 35.96 | 30.54 | 28.36 | 18.00 | 29.61 | 306.55 | 275.11 | 648.05 | 711.59 | 703.92 | 421.27 | -4.15 | 8.07 | 9.61E-17 | 3.21E-15 |
| U37 | 99.57 | 63.35 | 75.27 | 119.01 | 121.34 | 1160.51 | 1389.32 | 1464.12 | 1925.48 | 1078.35 | 451.37 | -3.69 | 9.45 | 1.96E-14 | 5.46E-13 |
| U58C | 109.71 | 170.44 | 115.09 | 212.39 | 100.95 | 1620.33 | 797.83 | 1560.13 | 3055.66 | 1332.96 | 1975.98 | -3.60 | 9.94 | 2.49E-14 | 5.95E-13 |
| U15A | 6911.99 | 2595.78 | 3377.85 | 2285.05 | 2043.35 | 153.27 | 591.49 | 120.01 | 188.36 | 104.84 | 210.64 | 3.89 | 10.75 | 1.26E-13 | 2.34E-12 |
| HBII-142 | 2720.42 | 2313.36 | 4186.73 | 3567.34 | 3181.02 | 32581.77 | 28597.90 | 31322.65 | 11678.48 | 33488.74 | 15396.58 | -3.00 | 13.91 | 1.26E-13 | 2.34E-12 |
| U63 | 18888.66 | 20295.31 | 24019.69 | 17031.38 | 18081.95 | 2583.77 | 3920.35 | 1512.13 | 1004.60 | 1737.34 | 3881.74 | 3.01 | 13.33 | 1.60E-13 | 2.67E-12 |
| U59B | 242.17 | 209.28 | 195.81 | 270.98 | 219.87 | 1729.81 | 2132.12 | 1344.11 | 1569.69 | 1273.05 | 1364.13 | -2.78 | 9.88 | 4.07E-13 | 6.17E-12 |
| U24 | 8056.15 | 9648.22 | 8083.29 | 10369.09 | 5966.48 | 963.44 | 660.27 | 1440.12 | 1716.19 | 1872.13 | 842.55 | 2.76 | 12.15 | 7.04E-13 | 9.79E-12 |
| HBI-43 | 5247.53 | 1262.82 | 2129.36 | 1668.01 | 2090.91 | 131.38 | 302.62 | 288.02 | 104.65 | 344.47 | 240.73 | 3.38 | 10.32 | 2.63E-12 | 3.38E-11 |
| HBI-100 | 183.16 | 201.74 | 230.17 | 121.45 | 215.01 | 2233.43 | 1416.83 | 1632.14 | 586.02 | 3235.05 | 1233.73 | -3.18 | 9.98 | 4.65E-11 | 5.55E-10 |
| ACA58 | 42.10 | 43.74 | 59.45 | 59.51 | 48.54 | 656.89 | 412.67 | 1992.17 | 3892.83 | 434.34 | 341.03 | -4.66 | 9.44 | 6.55E-11 | 7.29E-10 |
| SNORD121B | 478.50 | 355.58 | 285.81 | 262.13 | 178.61 | 21.90 | 13.76 | 24.00 | 0.00 | 59.91 | 20.06 | 3.70 | 7.47 | 2.74E-10 | 2.86E-09 |
| U45B | 397.98 | 634.99 | 237.26 | 357.65 | 284.42 | 0.00 | 68.78 | 48.00 | 20.93 | 29.95 | 40.12 | 3.39 | 7.75 | 9.07E-10 | 8.91E-09 |
| U50 | 318.39 | 429.87 | 460.89 | 544.41 | 385.37 | 2233.43 | 1801.98 | 4632.39 | 6467.11 | 1183.19 | 2648.01 | -2.88 | 10.89 | 2.24E-09 | 2.08E-08 |
| ACA26 | 122.31 | 61.09 | 96.54 | 32.04 | 131.05 | 1379.47 | 412.67 | 2064.17 | 4562.56 | 1347.94 | 240.73 | -4.23 | 9.84 | 4.11E-09 | 3.61E-08 |
| HBII-180C | 127.23 | 69.38 | 116.18 | 89.41 | 63.58 | 1072.92 | 385.16 | 1032.09 | 816.24 | 449.31 | 331.00 | -2.85 | 8.60 | 4.74E-09 | 3.96E-08 |
| U13 | 60.85 | 38.08 | 36.00 | 61.64 | 82.51 | 1423.26 | 13.76 | 3984.34 | 3139.38 | 1332.96 | 662.00 | -4.97 | 9.89 | 6.17E-09 | 4.83E-08 |
| U58B | 228.34 | 517.35 | 358.35 | 629.55 | 338.29 | 3722.38 | 1678.18 | 2424.21 | 2992.87 | 1902.09 | 1424.31 | -2.50 | 10.50 | 6.37E-09 | 4.83E-08 |
| hsa_piR_010894-3 | 433.94 | 219.83 | 973.59 | 339.34 | 317.91 | 43.79 | 68.78 | 24.00 | 41.86 | 0.00 | 60.18 | 3.47 | 7.98 | 7.92E-09 | 5.75E-08 |
| mgU6-77 | 169.95 | 142.16 | 251.44 | 253.89 | 247.05 | 919.65 | 880.36 | 1464.12 | 1653.40 | 1976.97 | 621.88 | -2.55 | 9.57 | 1.01E-08 | 7.01E-08 |
| U71b | 38.11 | 27.15 | 50.73 | 28.99 | 102.90 | 197.07 | 797.83 | 960.08 | 1988.27 | 644.01 | 120.36 | -3.98 | 8.73 | 1.88E-08 | 1.21E-07 |
| HBII-316 | 100.49 | 134.99 | 116.72 | 153.19 | 87.36 | 919.65 | 591.49 | 1536.13 | 2155.70 | 554.15 | 270.82 | -3.07 | 9.17 | 1.89E-08 | 1.21E-07 |

| | | | | | | | | | | | | | | |
|---|---|---|---|---|---|---|---|---|---|---|---|---|---|---|
| U104 | 13107.31 | 11522.66 | 12682.36 | 14415.84 | 7868.11 | 94548.44 | 66879.75 | 39723.36 | 24026.69 | 75095.06 | 44193.70 | -2.27 | 15.16 | 2.02E-08 | 1.25E-07 |
| U51 | 429.64 | 390.65 | 355.08 | 485.82 | 390.71 | 1926.88 | 8418.43 | 1320.11 | 1255.75 | 1962.00 | 5135.54 | -3.03 | 10.96 | 2.68E-08 | 1.60E-07 |
| ACA44 | 96.19 | 30.17 | 76.91 | 38.15 | 111.15 | 437.93 | 632.76 | 624.05 | 209.29 | 733.88 | 331.00 | -2.81 | 8.15 | 4.84E-08 | 2.79E-07 |
| HBII-276 | 3006.23 | 1401.96 | 1590.48 | 2061.06 | 1277.94 | 416.03 | 261.36 | 432.04 | 523.23 | 404.38 | 521.58 | 2.13 | 10.11 | 6.45E-08 | 3.47E-07 |
| HBII-202 | 1398.62 | 1333.71 | 1199.95 | 1928.62 | 1449.27 | 262.76 | 261.36 | 120.01 | 481.37 | 464.29 | 280.85 | 2.22 | 9.74 | 9.65E-08 | 5.04E-07 |
| U28 | 361.41 | 704.38 | 465.80 | 861.17 | 591.65 | 2561.87 | 3108.77 | 2712.23 | 6111.32 | 1887.11 | 1695.13 | -2.33 | 10.89 | 1.29E-07 | 6.54E-07 |
| HBII-296A | 5229.71 | 1931.38 | 1867.01 | 2203.27 | 1158.06 | 175.17 | 605.25 | 312.03 | 104.65 | 434.34 | 651.97 | 2.68 | 10.41 | 1.78E-07 | 8.70E-07 |
| U84 | 1483.14 | 851.06 | 968.69 | 1032.06 | 715.42 | 7291.48 | 20949.79 | 4272.36 | 1339.47 | 4717.78 | 34715.02 | -3.60 | 12.80 | 1.82E-07 | 8.70E-07 |
| HBII-436 | 94.04 | 63.35 | 48.54 | 85.75 | 54.36 | 350.34 | 261.36 | 168.01 | 627.88 | 299.54 | 391.18 | -2.33 | 7.69 | 3.62E-07 | 1.68E-06 |
| hsa_piR_001168-4 | 547.34 | 141.40 | 323.44 | 163.26 | 619.80 | 65.69 | 13.76 | 96.01 | 20.93 | 29.95 | 60.18 | 2.96 | 7.71 | 6.29E-07 | 2.84E-06 |
| HBII-419 | 2309.84 | 3844.66 | 3093.14 | 3698.87 | 1867.65 | 29560.07 | 8666.03 | 17641.49 | 11992.41 | 17717.88 | 4222.78 | -2.34 | 13.21 | 7.06E-07 | 3.10E-06 |
| HBII-296B | 1532.00 | 388.76 | 380.71 | 596.29 | 478.08 | 43.79 | 68.78 | 216.02 | 125.58 | 89.86 | 150.46 | 2.55 | 8.61 | 1.40E-06 | 5.99E-06 |
| U106 | 420.42 | 309.20 | 199.08 | 146.17 | 412.55 | 897.75 | 990.40 | 1968.17 | 2281.28 | 2036.88 | 682.06 | -2.31 | 9.85 | 1.53E-06 | 6.39E-06 |
| U25 | 1464.70 | 1564.86 | 1986.46 | 2634.77 | 976.05 | 12896.95 | 5405.95 | 5184.44 | 6613.62 | 8012.74 | 3470.50 | -2.00 | 12.15 | 2.00E-06 | 8.16E-06 |
| U58A | 393.07 | 739.07 | 608.16 | 1048.84 | 580.00 | 3809.97 | 1884.52 | 3384.29 | 3767.25 | 1407.85 | 1996.04 | -2.00 | 10.78 | 2.29E-06 | 9.11E-06 |
| mgU12-22/U4-8 | 209.90 | 194.95 | 210.54 | 249.62 | 163.57 | 0.00 | 55.02 | 24.00 | 20.93 | 14.98 | 80.24 | 2.47 | 7.02 | 3.01E-06 | 1.17E-05 |
| U36B | 476.35 | 501.13 | 291.81 | 437.60 | 346.54 | 0.00 | 96.29 | 24.00 | 188.36 | 44.93 | 40.12 | 2.65 | 7.92 | 3.81E-06 | 1.44E-05 |
| U83A | 108.18 | 286.58 | 179.99 | 291.43 | 338.29 | 1072.92 | 1788.23 | 2064.17 | 1276.68 | 599.08 | 431.30 | -2.32 | 9.54 | 4.69E-06 | 1.74E-05 |
| U97 | 303.33 | 200.60 | 283.62 | 143.73 | 379.55 | 21.90 | 82.53 | 48.00 | 20.93 | 59.91 | 110.33 | 2.08 | 7.39 | 2.19E-05 | 7.64E-05 |
| mgU2-25/61 | 137.68 | 86.35 | 85.09 | 51.27 | 212.59 | 394.13 | 1554.38 | 456.04 | 397.65 | 299.54 | 260.79 | -2.29 | 8.43 | 5.61E-05 | 1.80E-04 |
| 14q(II-1) | 64.54 | 58.82 | 130.36 | 85.75 | 211.62 | 175.17 | 481.45 | 4584.39 | 83.72 | 2336.42 | 210.64 | -3.57 | 9.52 | 5.89E-05 | 1.86E-04 |
| ACA7B | 254.16 | 94.65 | 311.44 | 76.90 | 126.68 | 1532.74 | 275.11 | 1104.09 | 669.73 | 524.20 | 601.82 | -2.17 | 8.92 | 6.09E-05 | 1.86E-04 |
| ACA3 | 115.25 | 30.54 | 47.45 | 28.99 | 102.41 | 197.07 | 261.36 | 360.03 | 125.58 | 599.08 | 240.73 | -2.20 | 7.47 | 6.11E-05 | 1.86E-04 |
| U85 | 37.19 | 36.95 | 37.63 | 29.91 | 45.14 | 43.79 | 247.60 | 72.01 | 104.65 | 104.84 | 421.27 | -2.22 | 6.65 | 7.02E-05 | 2.09E-04 |
| Z17B | 112.48 | 90.88 | 66.00 | 73.85 | 55.33 | 0.00 | 13.76 | 0.00 | 20.93 | 29.95 | 10.03 | 2.58 | 5.81 | 7.93E-05 | 2.28E-04 |
| snR38B | 4048.05 | 3574.67 | 5181.05 | 2906.67 | 2273.41 | 16947.78 | 35173.08 | 7536.64 | 3871.90 | 18736.32 | 6790.54 | -2.05 | 13.25 | 8.10E-05 | 2.29E-04 |
| U82 | 8460.59 | 6273.02 | 4618.16 | 6182.88 | 2920.87 | 1051.02 | 2104.61 | 600.05 | 376.73 | 284.56 | 3791.47 | 2.05 | 11.71 | 3.69E-04 | 9.78E-04 |
| ACA8 | 46.41 | 35.45 | 53.45 | 51.57 | 61.15 | 0.00 | 13.76 | 24.00 | 0.00 | 14.98 | 10.03 | 2.21 | 5.26 | 1.95E-03 | 4.40E-03 |
| U90 | 121.70 | 126.32 | 93.81 | 134.58 | 428.57 | 65.69 | 27.51 | 144.01 | 41.86 | 0.00 | 20.06 | 2.00 | 6.93 | 3.64E-03 | 7.42E-03 |
