# Supplementary material for: snoRNA and piRNA expression levels modified by tobacco use in women with lung adenocarcinoma
Source: PLoS One. 2017 Aug 17;12(8):e0183410. doi: 10.1371/journal.pone.0183410 (PMC5560661; doi:10.1371/journal.pone.0183410)
Supplement: S10 File — (PDF) [file pone.0183410.s010.pdf]

Supplemental File 10

snoRNA and piRNA analysis

Normal Non-Smoker x Tumor Non-Smoker

for the manuscript: “snoRNA and piRNA expression levels modified by tobacco use in women with lung adenocarcinoma” by

Natasha Andressa Nogueira Jorge, Gabriel Wajnberg, Carlos Gil Ferreira, Benilton de Sa Carvalho, Fabio Passetti

The CPM counts were calculated using the EdgeR Bioconductor package and normalized using the TMM methodology. Figure 1 shows the total raw and normalized counts.

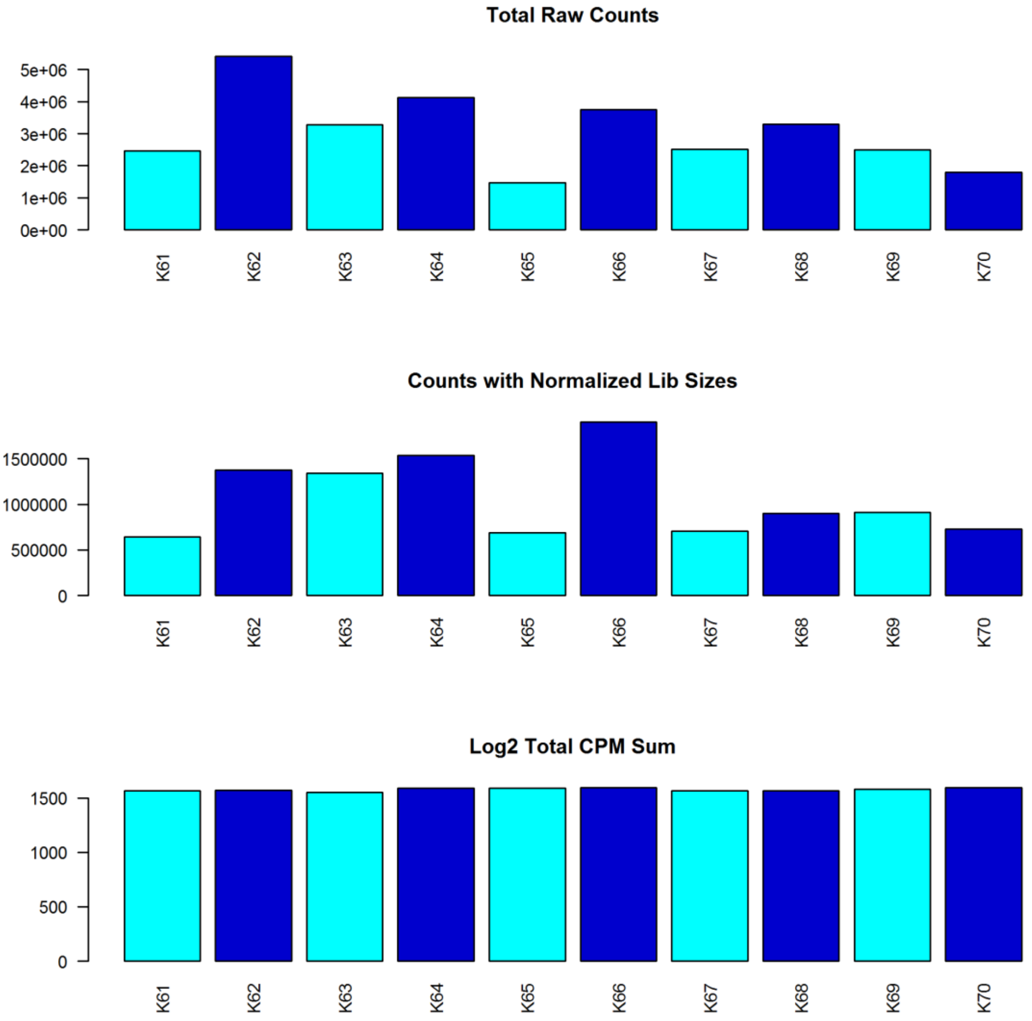

Figure 1. Raw, Normalized and log2 Normalized Total Counts. Light blue bars indicate normal samples and dark blue bars indicate tumor samples.

Hierarchical clustering was performed on the normalized CPM counts (Figure 2). The samples show some variability because two tumor samples, K68 and K70, were classified as normal. However, there is still a distinction between non-smokers and smokers.

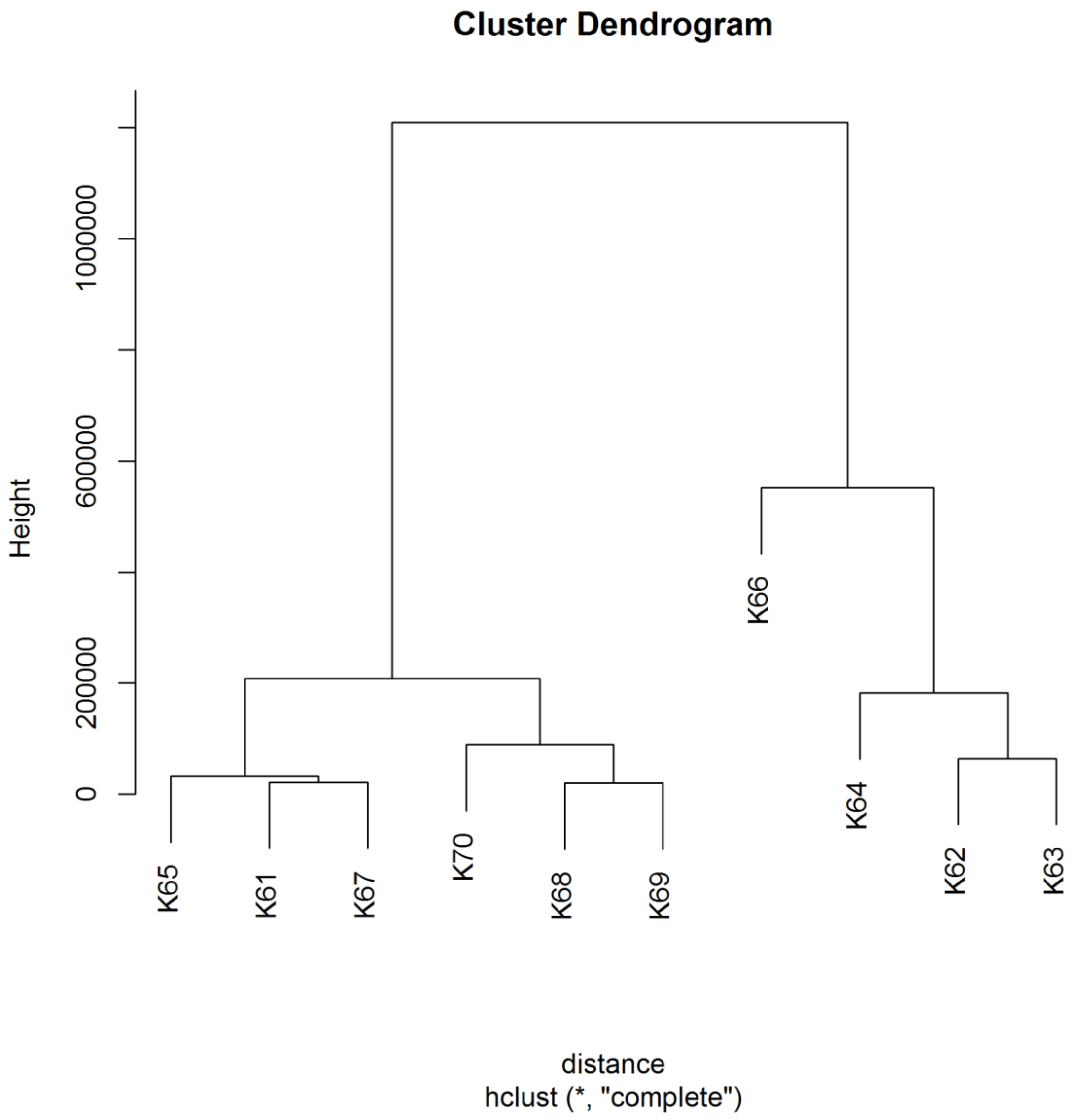

Figure 2. Hierarchical cluster for normalized counts. Samples ending in odd numbers correspond to normal samples and the ones ending in even numbers to tumor samples.

In order to further investigate the distribution of our samples, we used the normalized counts to perform principal component analysis. As noticed on the hierarchical clustering analysis, the PCA also did not show a clear distinction between normal and tumor samples (Figure 3).

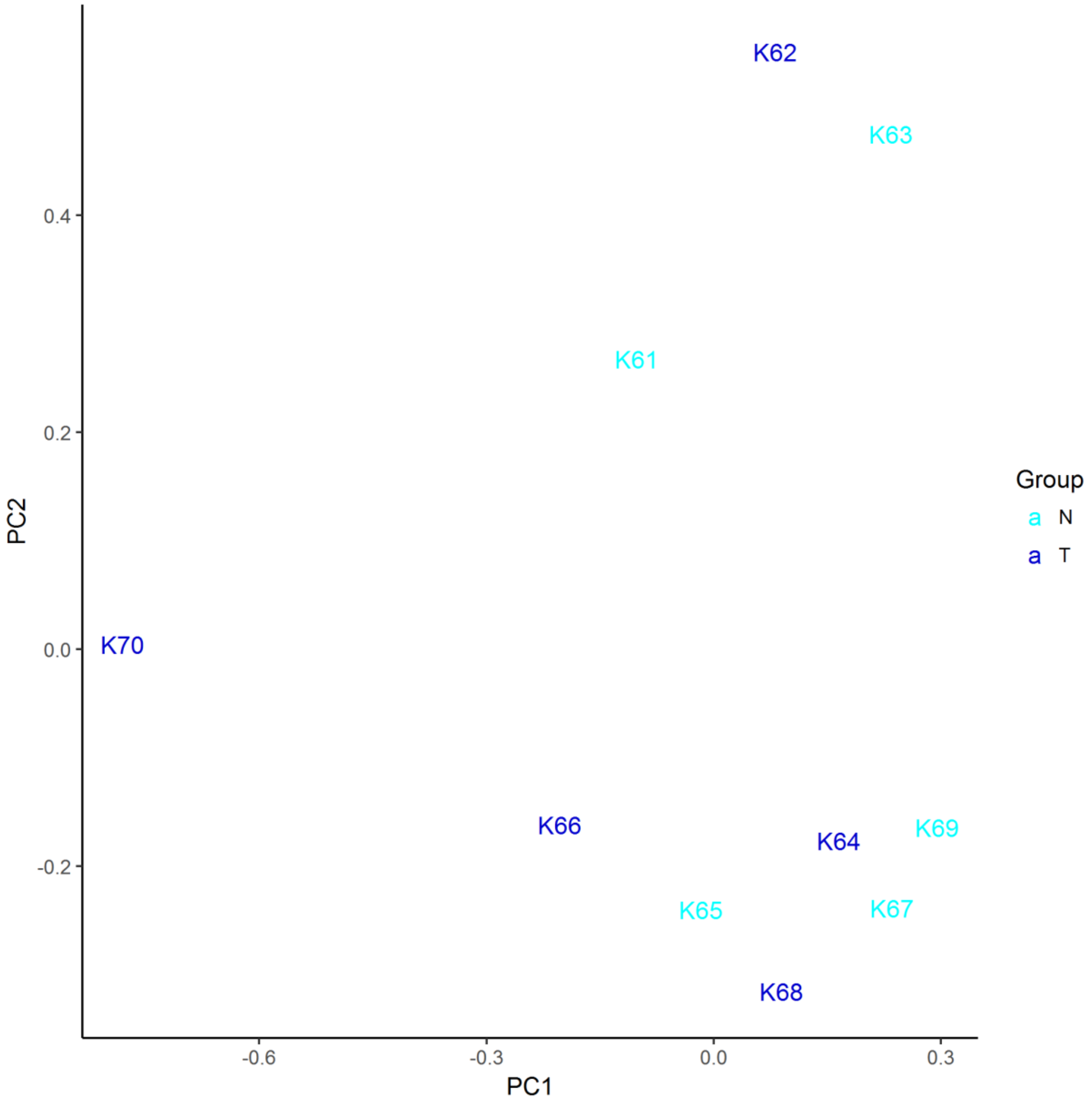

Figure 3. PCA analysis.

After applying our differential expression filters, FDR < 0.01 and logFC > 2 or logFC < -2, no snoRNA or piRNAs was found differentially expressed between normal and tumor samples (igure 4).

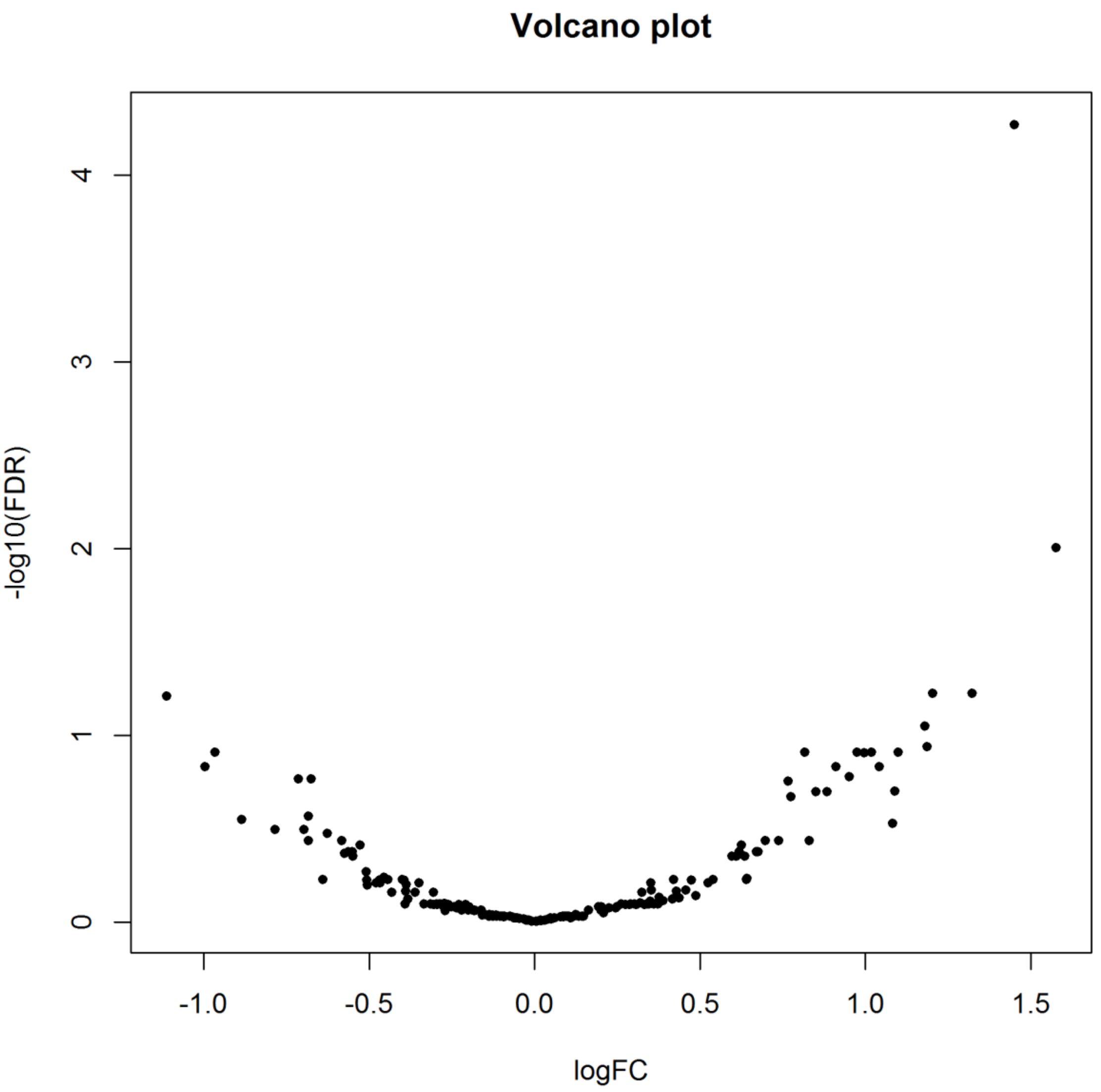

Figure 4. Volcano Plot. The red dots indicate the differentially expressed genes found. The genes on the left side of the plot are up-regulated in non-smokers and the ones one the right side are down-regulated.
