# Supplementary material for: snoRNA and piRNA expression levels modified by tobacco use in women with lung adenocarcinoma
Source: PLoS One. 2017 Aug 17;12(8):e0183410. doi: 10.1371/journal.pone.0183410 (PMC5560661; doi:10.1371/journal.pone.0183410)
Supplement: S11 File — (PDF) [file pone.0183410.s011.pdf]

Supplemental File 11

snoRNA and piRNA analysis

Normal Smoker x Tumor Smoker

The CPM counts were calculated using the EdgeR Bioconductor package and normalized using the TMM methodology. Figure 1 shows the total raw and normalized counts.

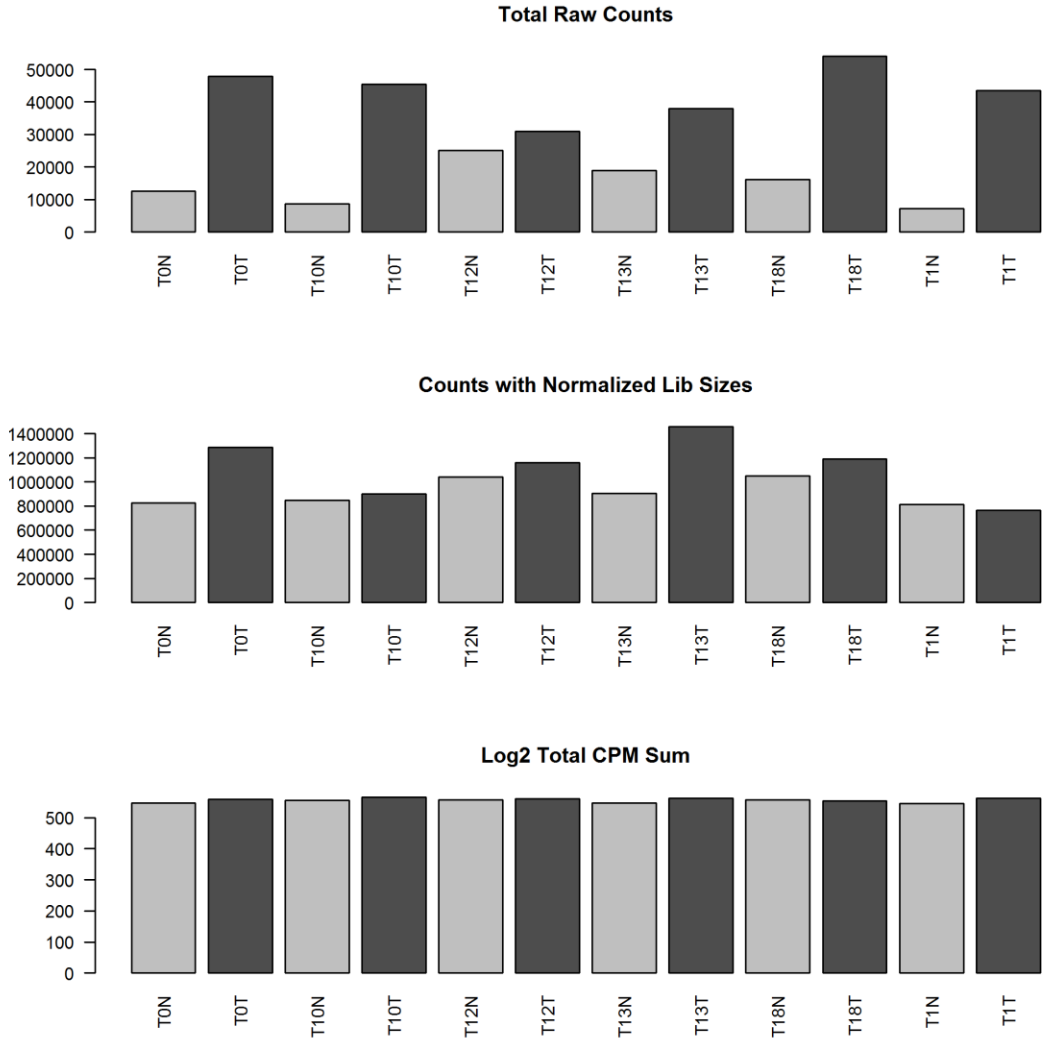

Figure 1. Raw, Normalized and log2 Normalized Total Counts. Light gray bars indicate normal samples and dark gray bars indicate tumor samples.

Hierarchical clustering was performed on the normalized CPM counts (Figure 2). The samples show some variability because two tumor samples, T10T and T1T, were classified as normal. However, there is still a distinction between non-smokers and smokers.

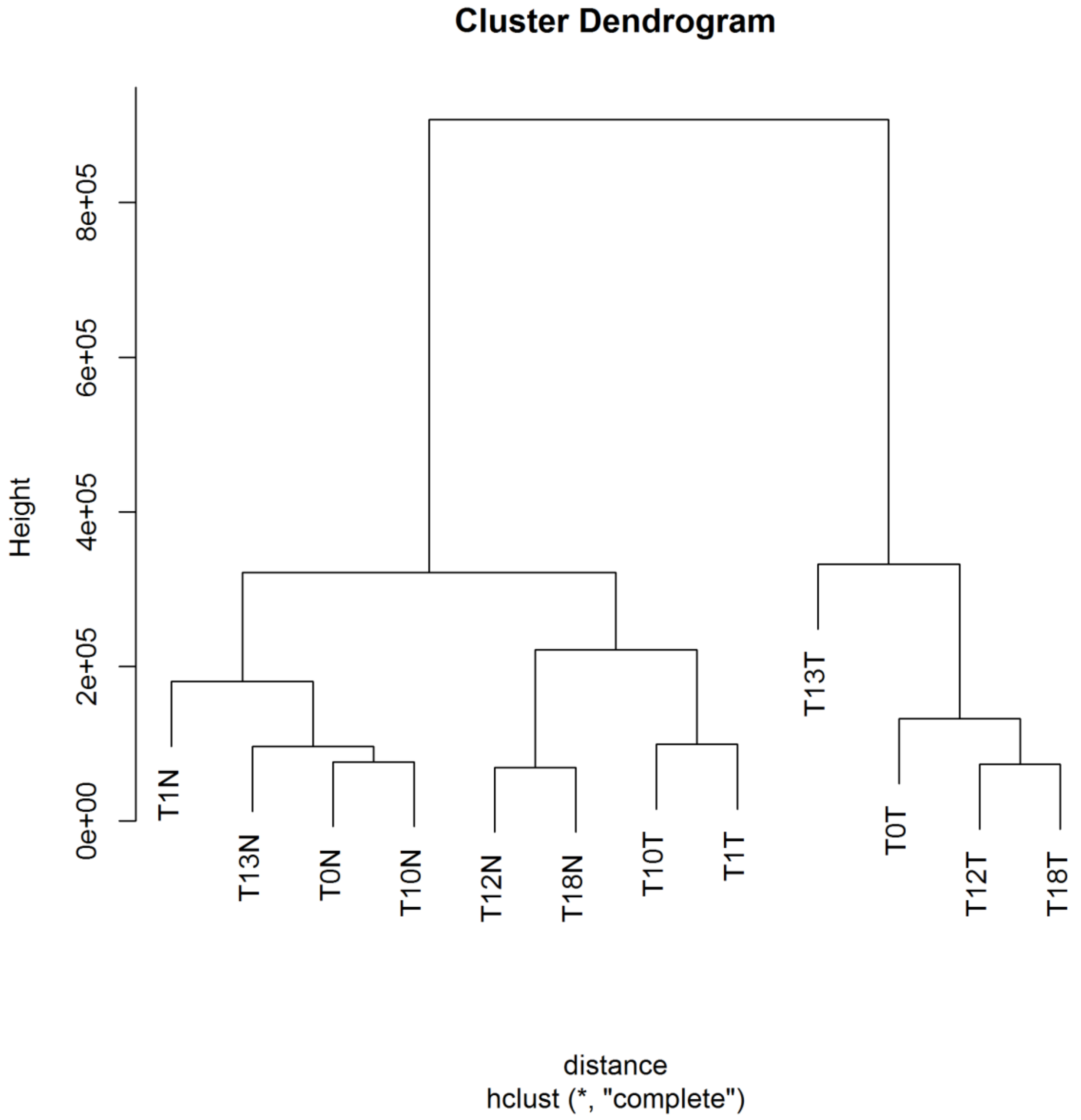

Figure 2. Hierarchical cluster for normalized counts. Samples ending in 'N' correspond to normal samples and the ones ending in 'T' to tumor samples.

In order to further investigate the distribution of our samples, we used the normalized counts to perform principal component analysis. Differently from the hierarchical clustering, the PCA was able to distinct between normal and tumor samples (Figure 3).

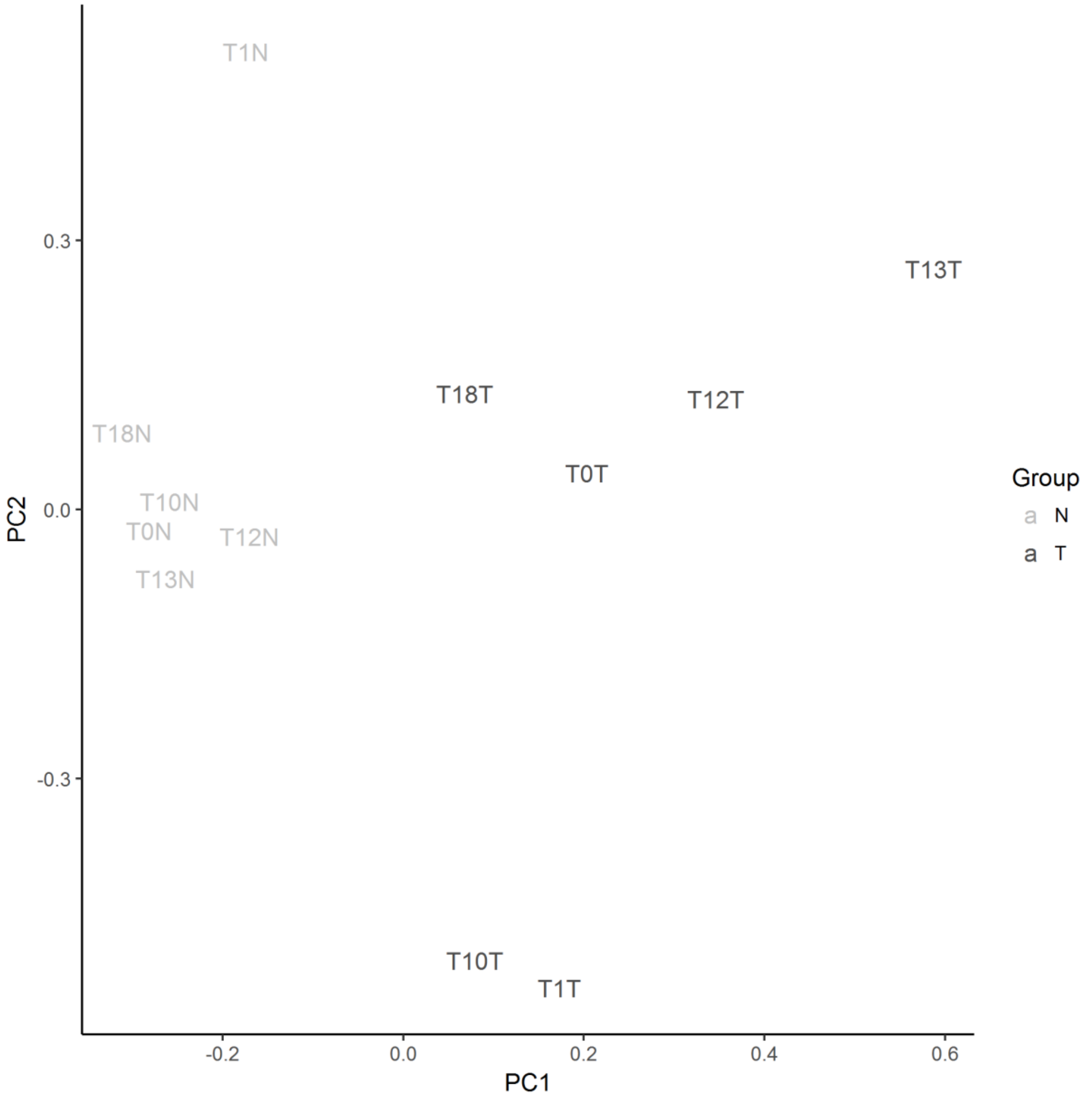

Figure 3. PCA analysis.

After applying our differential expression filters, FDR < 0.01 and logFC > 2 or logFC < -2, we found 6 differentially expressed snoRNA (igure 4). Only one snoRNA was found up-regulated in normal samples, while the rest are up-regulated in tumor samples (Figure 5). Table 1 shows the normalized cpm counts, logFC, and FDR for the differentially expressed snoRNAs.

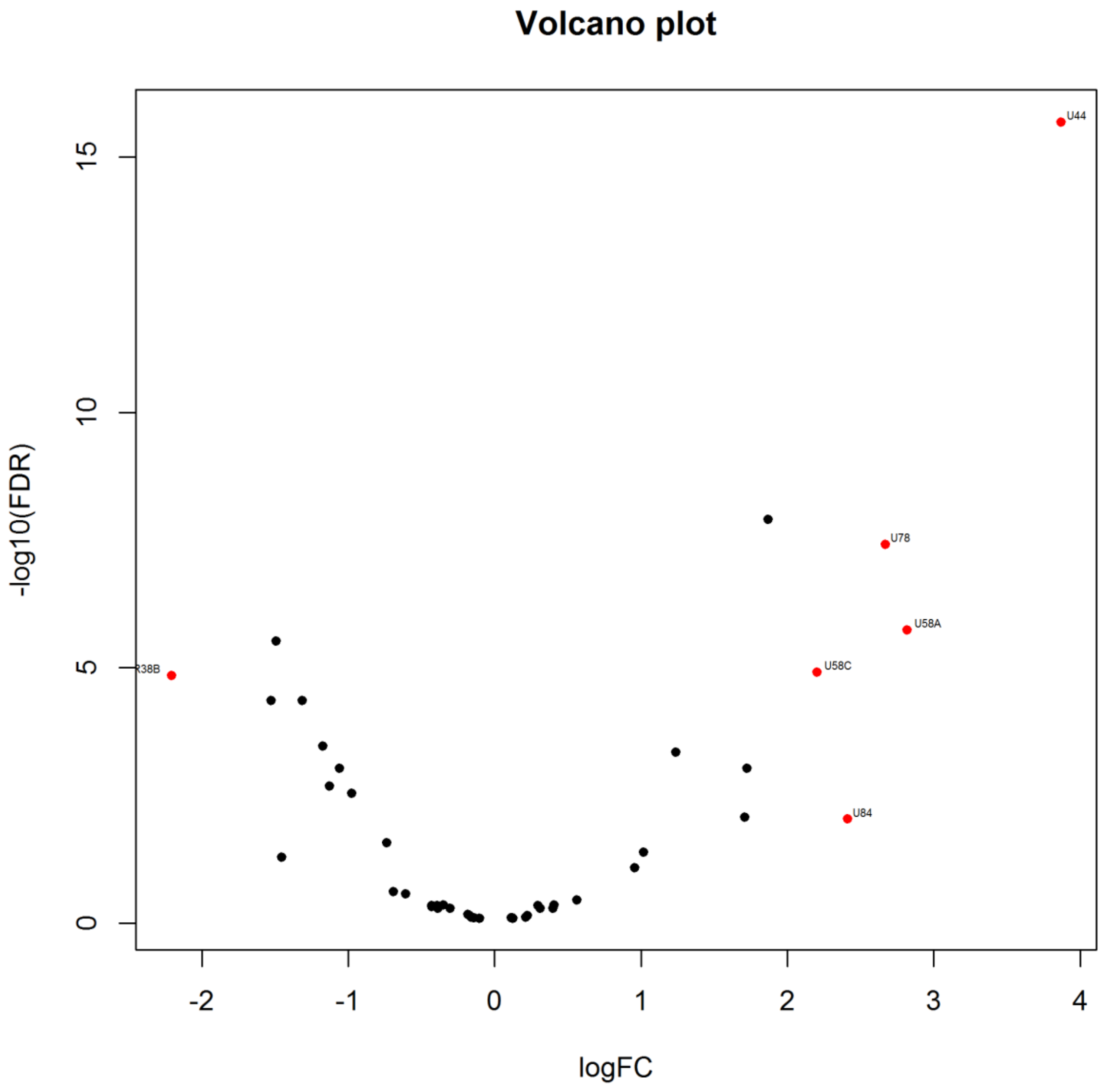

Figure 4. Volcano Plot. The red dots indicate the differentially expressed genes found. The genes on the left side of the plot are up-regulated in non-smokers and the ones one the right side are down-regulated.

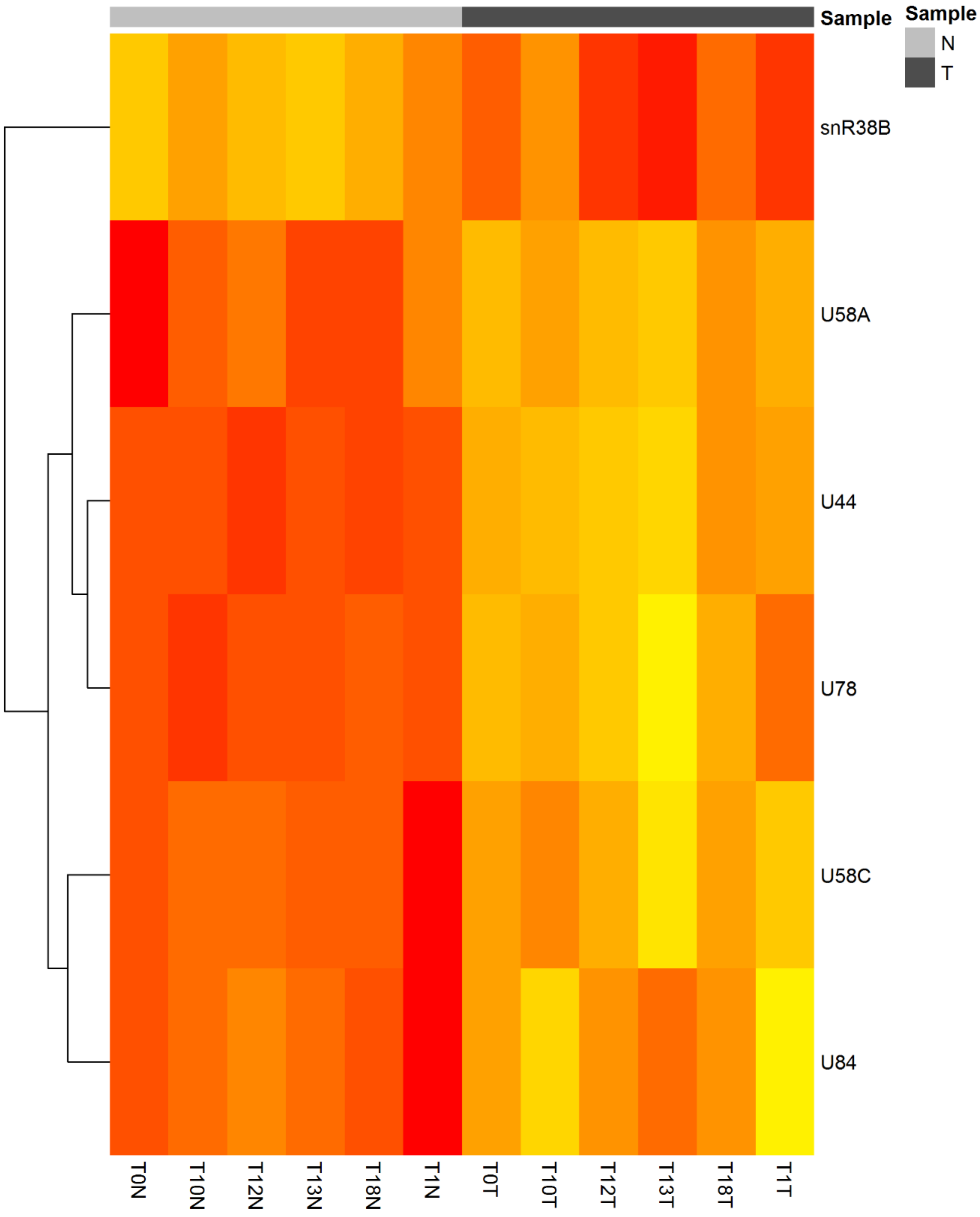

Figure 5. Differentially expressed snoRNAs.

Table 1. Differentially expressed snoRNAs.

Gene	T0N	T0T	T10N	T10T	T12N	T12T	T13N	T13T	T18N	T18T	T1N	T1T	logFC	logCPM	LR	PValue	FDR
U44	788.58	6763.85	683.94	10888.76	373.59	12832.99	670.00	19679.84	517.42	3934.48	684.80	5827.61	3.87	12.41	75.03	4.63E-18	2.04E-16
U78	1051.44	6602.81	586.23	5088.02	1120.76	8642.63	957.15	16950.80	1228.86	5341.23	1027.21	1831.03	2.67	12.08	35.46	2.61E-09	3.82E-08
U58A	65.72	4670.28	586.23	2712.29	1079.25	5275.37	382.86	6918.69	388.06	2066.15	1597.88	3503.61	2.82	11.32	27.40	1.65E-07	1.82E-06
U58C	525.72	1986.21	683.94	1148.27	705.67	2431.91	622.15	5611.83	582.09	1956.25	114.13	3468.40	2.20	10.80	22.94	1.67E-06	1.22E-05
snR38B	112833.05	20774.69	65071.54	50622.83	94559.27	11747.98	121557.98	7110.88	86796.55	27497.43	38919.68	11919.31	-2.21	15.72	22.37	2.25E-06	1.42E-05
U84	1380.02	8937.95	2833.45	30151.96	5230.23	6659.69	2967.16	2459.98	1358.22	6923.81	228.27	60934.60	2.41	13.43	8.50	3.55E-03	9.18E-03
