# Supplementary material for: snoRNA and piRNA expression levels modified by tobacco use in women with lung adenocarcinoma
Source: PLoS One. 2017 Aug 17;12(8):e0183410. doi: 10.1371/journal.pone.0183410 (PMC5560661; doi:10.1371/journal.pone.0183410)
Supplement: S12 File — (PDF) [file pone.0183410.s012.pdf]

Supplemental File 12

Constitutive snoRNA analysis

Non-smokers

for the manuscript: “snoRNA and piRNA expression levels modified by tobacco use in women with lung adenocarcinoma” by

Natasha Andressa Nogueira Jorge, Gabriel Wajnberg, Carlos Gil Ferreira, Benilton de Sa Carvalho, Fabio Passetti

To identify the snoRNAs or piRNAs which expression levels that do not change between normal and tumor samples w performed a dispersion analysis. We considered as constitutive all the genes which expression is greater than 1 CPM in all samples, none of the samples are 2 times greater or lower than the average log2 expression, and standard deviation is lower than 1. In this analysis, we found 179 constitutive sncRNAs. Table *1* shows the log2 CPM for each evaluated sample, variance, and standard deviation of all genes

Table 1. Constitutively expressed sncRNAs.

**Genes**	**K61**	**K62**	**K63**	**K64**	**K65**	**K66**	**K67**	**K68**	**K69**	**K70**	**Var**	**SD**
U51	8.45	8.48	8.54	8.58	8.79	8.37	8.57	8.76	8.53	8.35	0.02	0.15
U57	12.20	12.38	12.34	12.30	12.07	11.92	12.37	12.36	12.25	11.67	0.05	0.23
HBII-429	9.42	9.37	9.57	9.40	9.81	9.60	9.34	8.92	9.72	9.42	0.06	0.25
HBII-95	5.89	5.62	5.37	5.94	5.87	5.94	5.77	6.12	5.38	5.63	0.06	0.25
U21	13.34	13.27	14.15	13.56	13.60	13.52	13.54	13.68	13.77	13.30	0.07	0.26
U35B	4.98	4.85	4.84	4.14	4.83	4.45	4.93	4.82	4.96	4.74	0.07	0.26
U37	6.65	6.37	5.98	5.95	6.25	6.13	6.21	6.73	6.32	6.67	0.08	0.28
U75	9.08	9.38	8.77	9.23	8.66	8.88	9.01	8.94	8.90	8.42	0.08	0.28
U95	12.08	12.11	12.30	11.66	12.48	11.85	12.26	11.87	12.36	12.45	0.08	0.28
ACA8	5.96	5.27	4.95	5.11	5.50	5.64	5.45	5.52	5.36	5.68	0.08	0.29
mgh28S-2411	11.48	12.00	11.48	12.16	12.15	12.05	12.01	12.25	11.97	11.57	0.08	0.29
U15B	9.48	8.86	8.70	8.81	9.27	8.98	8.83	8.77	8.42	8.95	0.09	0.29
U38B	9.69	9.60	9.17	9.56	10.27	9.62	9.92	9.87	9.85	9.86	0.08	0.29
U59B	7.89	7.65	7.28	7.67	8.24	7.51	8.10	7.92	8.00	7.52	0.09	0.3
HBII-276	10.68	11.28	10.73	10.42	10.80	10.53	10.90	10.84	10.86	10.06	0.11	0.32
HBII-85-24	5.90	6.18	5.69	5.89	6.09	5.13	6.07	5.72	5.98	5.48	0.1	0.32
HBII-85-23	4.57	4.71	4.87	5.02	4.32	4.27	4.90	4.43	4.55	3.95	0.11	0.33
U20	10.71	10.54	11.16	11.11	10.63	10.23	10.56	10.52	11.11	10.37	0.11	0.33
HBII-316	6.20	6.38	6.56	7.04	6.67	6.77	6.91	7.09	6.97	6.19	0.11	0.34
mgU2-19/30	8.01	8.68	8.29	7.70	7.83	7.97	7.98	7.75	7.92	7.40	0.12	0.34
U17b	8.12	8.01	8.01	7.98	8.33	8.49	7.76	8.02	7.76	8.86	0.12	0.34
U47	8.98	8.76	8.32	8.86	9.15	9.05	9.29	8.80	9.08	9.58	0.11	0.34
U88	5.30	5.57	6.16	5.74	5.80	5.70	5.16	5.43	5.30	5.04	0.12	0.34
snR38C	7.60	8.41	7.45	8.11	8.41	8.50	8.19	8.34	7.97	7.84	0.13	0.36
U101	10.15	10.49	10.42	10.15	9.93	10.19	10.14	10.11	10.16	9.18	0.13	0.36
U74	11.15	11.11	11.15	10.58	11.04	11.33	10.53	10.66	10.61	11.60	0.13	0.36
U81	10.70	11.21	11.39	11.03	11.10	11.32	11.45	11.18	11.64	10.44	0.13	0.36
U87	7.18	7.04	6.99	6.65	7.76	6.81	6.70	6.81	7.16	6.51	0.13	0.36
SNORD127	8.28	7.61	7.94	8.26	8.72	8.10	8.31	8.20	8.66	8.80	0.13	0.37
U27	13.88	14.26	14.27	14.51	14.37	14.61	14.52	14.97	14.38	13.64	0.14	0.37
U30	11.30	11.38	11.66	12.14	12.01	11.73	11.90	12.28	11.94	11.17	0.14	0.37
hsa-mir-1201	6.40	6.66	6.93	7.61	7.11	6.68	7.28	6.74	7.18	6.51	0.15	0.38

mgU12-22/U4-8	7.86	7.44	8.05	7.57	8.44	7.62	8.05	7.80	7.98	7.10	0.14	0.38
U59A	8.66	8.93	9.29	8.85	9.56	8.88	9.47	9.06	9.31	8.33	0.15	0.38
U63	13.05	13.93	13.78	14.27	14.04	14.45	13.70	13.89	14.12	13.89	0.14	0.38
U84	9.91	10.26	10.58	9.70	10.27	9.82	10.07	9.85	10.31	9.23	0.15	0.38
HBII-234	5.92	5.95	5.13	6.45	6.12	6.15	5.94	6.17	5.73	6.49	0.15	0.39
HBII-85-15	5.17	4.74	4.52	5.24	5.27	4.88	5.73	5.20	5.18	4.43	0.15	0.39
U26	11.44	11.01	11.38	11.87	11.86	11.83	12.00	12.22	12.10	11.38	0.15	0.39
HBII-210	10.69	11.71	11.55	12.00	11.97	12.00	11.65	11.91	11.87	11.40	0.16	0.4
SNORD121B	7.91	8.63	8.39	8.44	8.24	8.06	7.84	7.87	7.84	7.22	0.16	0.4
U24	13.12	12.70	13.84	13.20	12.92	12.88	13.00	13.18	13.14	12.29	0.16	0.4
U36B	8.72	8.62	9.53	8.93	8.70	8.09	8.76	8.61	8.68	8.18	0.16	0.4
U83	7.74	7.19	7.59	7.03	7.50	7.38	7.02	6.52	7.21	7.88	0.16	0.4
snR38B	11.12	11.71	11.71	11.77	11.79	12.24	11.31	11.34	11.98	10.89	0.17	0.41
U50	8.39	8.04	8.14	8.71	8.56	8.75	9.09	8.92	9.31	8.33	0.17	0.41
HBII-85-29	5.04	5.68	5.28	5.39	4.87	4.38	5.67	5.26	5.76	5.29	0.18	0.42
U31	13.46	13.68	13.68	14.22	14.01	14.38	13.94	14.62	14.24	13.33	0.17	0.42
HBII-82B	7.90	7.22	7.82	6.93	7.91	6.85	7.83	7.65	7.92	7.96	0.19	0.43
U48	10.90	12.06	11.17	11.48	11.15	11.92	11.03	11.41	12.09	11.53	0.18	0.43
U53	6.77	7.62	7.49	7.26	6.74	6.40	7.38	6.63	7.32	6.64	0.19	0.43
U96a	8.37	7.40	7.28	8.05	8.03	8.51	7.92	8.13	7.65	8.52	0.19	0.43
Z17B	6.66	6.54	6.37	6.47	5.65	5.94	5.91	6.04	5.51	5.53	0.18	0.43
HBII-202	10.65	10.18	11.14	10.35	10.76	10.13	11.23	10.75	11.27	10.24	0.19	0.44
U105	8.07	8.11	8.22	8.03	8.92	8.70	8.54	8.81	8.57	7.49	0.19	0.44
U36C	8.56	8.80	9.23	8.58	8.23	7.85	8.04	7.83	8.23	8.18	0.19	0.44
U42B	9.69	9.13	9.01	9.68	9.32	9.23	10.22	9.79	9.89	8.87	0.19	0.44
U45B	8.69	8.37	7.96	9.28	8.10	7.79	8.38	8.32	8.16	7.89	0.19	0.44
U80	8.72	8.46	8.34	7.53	7.53	7.46	8.12	7.72	7.94	8.31	0.2	0.44
HBII-420	9.47	8.92	8.13	8.31	8.69	9.25	8.63	9.22	8.71	9.35	0.2	0.45
U23	4.53	4.38	4.35	4.77	4.25	5.03	4.67	4.21	3.46	3.91	0.2	0.45
U76	12.41	12.89	12.78	12.15	11.53	12.07	11.87	11.68	12.13	11.81	0.2	0.45
HBII-239	9.99	9.22	10.17	9.47	10.73	9.51	10.05	9.81	10.36	9.71	0.21	0.46
HBII-55	9.74	10.07	10.51	10.07	10.06	9.85	10.14	9.99	10.17	8.77	0.21	0.46
U18B	7.44	6.92	6.32	7.39	7.31	7.25	6.89	7.19	6.62	7.94	0.21	0.46
U73a	8.93	8.68	8.29	7.75	9.00	8.03	8.71	8.57	8.10	9.11	0.21	0.46
U97	8.09	7.97	8.45	7.61	7.95	8.05	7.29	7.00	7.46	8.31	0.21	0.46
HBII-108B	9.05	8.69	8.06	9.18	8.24	8.67	9.30	9.36	9.00	8.25	0.22	0.47
HBII-251	11.12	11.24	11.78	12.10	12.10	12.07	11.84	12.02	12.45	11.08	0.22	0.47
U105B	5.86	5.45	5.44	5.73	6.67	6.43	6.67	6.52	6.11	6.13	0.22	0.47
U49A	7.39	7.55	6.99	6.70	6.65	6.99	6.14	6.30	6.30	6.49	0.22	0.47
ACA61	6.86	7.14	6.88	6.33	7.15	7.25	6.26	6.94	6.72	7.93	0.23	0.48
HBII-419	10.49	10.90	11.02	11.87	11.48	11.49	11.52	11.69	11.60	10.61	0.23	0.48
HBII-99	4.26	4.90	3.32	4.90	4.15	4.73	4.51	4.01	4.57	4.63	0.23	0.48
snR39B	13.27	13.08	14.02	13.12	13.08	12.99	13.36	13.29	13.57	12.13	0.23	0.48

U13	6.33	5.66	5.41	5.22	6.40	5.07	6.31	5.78	5.77	6.11	0.23	0.48
U55	6.75	6.03	5.91	6.05	6.71	5.81	5.41	5.63	5.59	6.58	0.23	0.48
U58C	7.61	6.51	6.49	7.38	7.23	6.75	7.40	7.57	7.37	6.40	0.23	0.48
HBII-85-20	4.61	4.87	3.44	4.49	4.54	4.38	4.51	3.93	4.97	3.79	0.24	0.49
U28	9.32	8.23	8.11	9.43	8.72	8.76	9.14	9.59	8.59	8.95	0.24	0.49
U50B	8.44	8.28	8.93	7.59	7.81	7.71	8.15	7.95	8.30	7.20	0.24	0.49
U82	12.55	12.78	13.05	12.58	12.33	12.07	12.54	12.43	12.67	11.26	0.24	0.49
U94	6.03	5.18	6.00	5.43	6.83	6.06	5.50	5.32	5.69	5.51	0.24	0.49
ACA58	5.61	5.12	5.75	5.42	4.68	5.79	5.95	5.73	6.54	5.34	0.25	0.5
HBII-142	10.86	11.14	11.96	11.14	12.39	11.93	11.90	11.64	12.13	11.38	0.25	0.5
mgh18S-121	9.42	8.88	8.83	10.14	9.16	8.53	9.57	9.21	9.99	9.38	0.25	0.5
mgU6-77	7.73	7.14	6.48	7.12	8.02	7.87	7.92	7.82	7.11	7.69	0.25	0.5
SNORD123	6.58	7.53	7.87	6.79	8.08	7.10	7.76	7.35	7.76	7.83	0.25	0.5
U8	8.92	7.17	7.76	7.75	7.91	8.03	7.37	7.64	7.40	7.39	0.25	0.5
U25	9.62	10.25	9.95	10.58	10.55	10.86	10.63	11.20	10.07	9.67	0.27	0.52
U54	6.40	6.78	6.24	6.61	6.65	5.70	6.84	6.46	6.62	5.21	0.27	0.52
HBII-85-1	4.85	5.05	4.49	4.86	4.29	3.78	5.02	4.08	5.02	3.65	0.28	0.53
SNORD119	12.99	13.43	13.70	12.79	12.89	12.53	13.19	12.73	13.55	11.89	0.29	0.53
U35A	4.92	4.33	3.54	4.20	4.96	4.42	4.99	4.78	4.01	5.25	0.28	0.53
U43	12.19	11.64	12.48	13.20	12.89	13.05	13.01	13.24	12.90	12.16	0.29	0.53
HBII-295	11.64	10.07	11.07	11.70	11.55	11.76	11.85	11.84	11.56	11.30	0.29	0.54
hsa-mir-1259	7.07	6.95	5.91	7.90	6.33	6.88	7.00	7.09	6.39	6.65	0.29	0.54
U56	6.96	6.48	5.53	6.69	7.36	6.46	7.36	7.16	6.63	6.96	0.3	0.54
ACA33	4.48	4.98	3.74	5.24	4.36	5.05	4.06	3.74	4.16	4.83	0.3	0.55
U65	7.27	6.82	7.50	7.46	8.25	7.11	6.67	7.01	6.66	8.11	0.31	0.55
ACA57	6.54	8.17	8.34	7.78	8.04	8.26	7.71	7.50	8.03	7.13	0.32	0.56
HBII-240	7.24	6.32	5.96	7.24	5.64	6.15	6.37	6.83	5.85	6.20	0.31	0.56
HBII-296A	11.07	12.08	11.59	10.88	10.97	10.77	11.24	10.94	11.12	9.92	0.31	0.56
HBII-436	6.23	6.28	5.87	5.95	7.01	5.50	7.05	6.26	6.80	5.51	0.32	0.56
U61	11.83	11.83	12.01	12.13	12.35	11.68	12.90	11.84	13.08	11.23	0.31	0.56
U83B	7.73	6.63	7.21	8.59	8.28	7.87	8.09	7.94	8.14	7.81	0.31	0.56
HBI-100	6.25	7.25	7.02	7.62	8.03	7.75	6.56	6.76	7.51	7.49	0.32	0.57
U16	6.57	5.81	5.34	5.07	5.73	4.88	4.60	5.20	5.01	5.59	0.32	0.57
ACA48	6.19	6.99	6.00	5.93	7.04	6.35	6.13	5.70	5.48	7.13	0.33	0.58
U52	11.75	12.22	11.13	10.67	10.57	11.26	10.46	11.11	10.48	10.70	0.34	0.58
U58B	8.66	7.56	7.61	8.98	8.81	8.38	9.14	9.13	8.70	8.15	0.34	0.58
U64	6.43	7.06	6.87	5.84	5.67	5.83	5.87	5.73	5.16	6.22	0.34	0.58
U71b	5.15	4.98	4.74	4.73	5.58	5.56	4.65	4.69	4.68	6.43	0.34	0.58
ACA45	9.64	10.98	10.71	9.98	10.43	9.99	9.58	9.17	10.06	10.85	0.35	0.59
HBII-13	9.14	9.15	9.42	9.19	9.75	8.89	9.68	9.22	9.82	7.77	0.34	0.59
HBII-296B	9.74	10.31	9.63	8.57	9.05	8.47	9.20	9.06	8.75	8.64	0.35	0.59
U29	9.16	8.06	8.48	9.00	9.45	9.01	9.81	9.78	9.37	9.85	0.35	0.59
U33	6.83	6.42	5.97	6.41	6.79	6.36	6.08	6.41	6.03	8.00	0.35	0.59

U85	5.94	4.95	5.56	5.17	6.61	5.13	4.74	4.74	4.78	5.24	0.36	0.6
U93	4.88	5.18	5.94	6.05	6.27	6.11	5.30	4.39	5.71	5.76	0.37	0.6
U104	12.92	13.41	13.86	13.46	14.65	13.53	14.07	13.65	14.40	12.68	0.37	0.61
U22	7.32	6.65	5.69	6.38	6.87	6.43	6.20	6.56	5.41	7.22	0.37	0.61
U44	12.05	12.68	12.31	13.89	12.60	13.87	12.72	13.27	12.67	12.70	0.38	0.61
U90	7.72	6.66	6.93	6.95	7.69	6.45	6.98	6.91	7.51	8.49	0.38	0.61
U18A	8.68	8.09	8.11	9.16	8.81	8.69	9.72	9.13	10.01	8.75	0.38	0.62
SNORD124	5.14	5.67	5.29	5.72	5.07	4.42	5.88	4.82	5.91	4.02	0.4	0.63
ACA42	3.78	4.83	4.06	4.93	5.11	4.38	4.90	5.47	4.36	5.85	0.4	0.64
U38A	8.20	9.46	8.35	10.16	9.04	9.56	8.34	8.53	8.71	8.81	0.4	0.64
U42A	9.41	8.46	8.71	10.11	9.84	9.93	10.11	9.97	10.20	10.26	0.41	0.64
ACA44	5.36	6.32	4.59	4.88	5.48	6.16	5.06	5.09	5.42	6.54	0.42	0.65
snR38A	6.53	7.52	6.58	8.58	6.94	7.82	6.88	7.44	6.76	6.88	0.42	0.65
U15A	12.20	12.48	12.30	11.31	11.07	11.62	10.81	10.99	11.06	10.74	0.42	0.65
U58A	9.76	8.35	7.90	9.49	9.58	9.15	9.73	9.87	9.25	8.92	0.42	0.65
HBII-135	8.72	8.44	8.27	7.70	7.44	6.98	9.22	8.15	7.97	7.56	0.43	0.66
HBII-180C	4.62	6.72	5.20	6.08	6.11	6.76	6.33	6.32	5.96	5.73	0.44	0.66
HBII-336	10.22	9.71	11.37	10.66	11.58	11.19	11.03	11.21	11.47	11.89	0.44	0.66
hsa_piR_010894-3	8.42	8.49	8.44	7.75	7.95	9.83	7.31	8.24	8.25	8.06	0.43	0.66
U106	8.78	8.44	8.26	8.24	6.86	7.54	8.30	7.03	8.51	8.43	0.44	0.66
U45A	9.26	9.12	9.47	10.79	9.51	9.21	10.20	9.77	10.70	8.92	0.43	0.66
ACA16	6.52	6.15	4.69	5.34	5.98	6.37	6.22	6.33	6.04	7.15	0.45	0.67
ACA18	5.49	5.36	4.78	5.75	5.58	5.74	4.87	5.04	5.04	7.12	0.45	0.67
ACA21	4.18	4.73	3.87	4.23	4.85	3.78	3.93	3.03	4.74	5.34	0.44	0.67
HBI-43	11.68	12.09	11.09	10.27	10.78	10.96	10.00	10.54	10.11	10.77	0.45	0.67
mgh28S-2409	9.15	8.16	7.46	9.33	8.86	8.98	9.57	9.76	9.11	9.24	0.46	0.68
mgU2-25/61	6.96	6.83	6.90	6.40	7.88	6.31	6.06	5.52	6.41	7.47	0.47	0.69
U83A	8.10	6.49	6.25	8.13	8.13	7.39	7.90	8.02	7.51	8.15	0.5	0.71
U46	7.72	7.04	5.91	6.56	6.95	5.82	6.85	6.87	6.27	8.15	0.54	0.73
U60	18.73	20.12	20.06	20.24	18.64	20.63	18.76	19.24	19.24	19.03	0.53	0.73
14q(II-1)	4.83	5.74	7.06	5.84	5.86	6.93	6.53	6.26	6.18	7.47	0.58	0.76
14q(II-3)	5.02	5.61	6.06	5.57	5.34	7.29	6.25	6.42	5.82	7.28	0.59	0.76
ACA7	5.83	7.59	5.98	6.92	7.04	8.55	6.92	6.91	7.14	7.18	0.58	0.76
U34	8.65	8.66	8.58	8.22	8.54	7.52	7.64	7.67	8.26	10.17	0.58	0.76
ACA26	4.51	6.66	5.39	5.90	5.44	6.49	5.15	4.84	5.78	6.78	0.6	0.77
U79	9.16	8.56	8.35	7.89	6.90	6.79	8.41	8.07	8.01	8.92	0.6	0.77
14q(0)	5.53	6.55	7.20	5.92	5.41	7.22	5.80	5.69	5.83	7.44	0.6	0.78
ACA3-2	6.29	5.63	4.59	4.51	5.32	4.48	5.57	5.28	4.55	6.77	0.63	0.79
HBII-95B	6.77	5.49	4.76	4.42	4.83	4.38	4.95	5.10	3.87	5.44	0.63	0.79
U45C	7.07	6.24	5.76	8.70	6.76	7.35	6.30	7.12	6.61	6.55	0.65	0.8
E2	6.16	4.64	4.41	6.33	6.00	6.04	4.32	5.18	5.38	6.39	0.65	0.81
hsa_piR_020828-2	9.01	9.04	9.94	7.78	8.51	8.91	7.80	7.24	8.19	9.21	0.66	0.81
U18C	7.00	6.73	5.00	8.08	6.04	7.05	6.99	7.14	6.39	6.88	0.65	0.81

ACA3	5.28	6.58	4.64	4.90	4.70	5.47	4.13	4.69	4.22	6.42	0.71	0.84
ACA50	5.35	3.83	3.95	5.19	4.92	3.73	4.62	4.37	4.89	6.52	0.71	0.84
ACA63	5.43	4.98	7.08	5.31	7.13	6.41	7.14	5.61	6.89	5.79	0.71	0.84
U17a	7.55	7.07	6.61	7.12	8.26	7.58	6.07	6.81	6.74	8.92	0.7	0.84
14q(II-17)	4.61	4.50	5.54	5.02	4.98	6.54	5.58	5.57	5.56	7.27	0.72	0.85
HBII-166	6.07	4.11	5.29	4.31	5.90	3.93	3.70	3.85	4.48	4.81	0.72	0.85
HBII-289	11.35	8.81	10.76	10.35	12.06	10.76	10.86	10.61	11.51	11.04	0.74	0.86
U32A	7.86	7.64	6.65	7.81	8.79	7.83	8.18	8.48	8.22	9.99	0.75	0.86
14q(I-8)	3.22	4.45	4.91	4.75	4.29	6.10	4.93	5.20	4.70	6.29	0.77	0.88
U78	11.71	13.10	12.28	13.98	10.99	12.99	11.65	12.75	11.34	12.39	0.84	0.92
14q(II-26)	3.44	5.33	4.93	5.07	4.78	7.14	4.89	5.67	5.19	5.95	0.89	0.94
HBII-85-22	4.77	5.68	5.41	4.29	3.42	3.55	4.67	4.01	5.75	3.16	0.89	0.94
hsa_piR_009294	10.98	11.77	12.88	12.50	13.62	13.50	13.38	12.73	13.67	11.60	0.89	0.94
U3	6.51	4.49	6.46	6.93	6.62	5.45	4.44	4.65	5.64	6.06	0.88	0.94
U102	10.26	8.67	9.58	9.90	10.57	11.31	9.19	8.68	9.80	8.28	0.89	0.95
ACA7B	5.02	7.72	5.41	6.53	6.19	8.18	6.04	6.10	5.98	6.73	0.93	0.96
14q(II-14)	3.61	4.14	4.82	4.42	5.07	6.26	4.79	5.29	5.42	6.99	0.99	0.99
hsa_piR_018165	7.70	7.93	7.04	7.04	5.53	6.59	5.42	5.42	5.82	7.56	0.98	0.99

Var: Variance. SD: Standard deviation
