# Supplementary material for: snoRNA and piRNA expression levels modified by tobacco use in women with lung adenocarcinoma
Source: PLoS One. 2017 Aug 17;12(8):e0183410. doi: 10.1371/journal.pone.0183410 (PMC5560661; doi:10.1371/journal.pone.0183410)
Supplement: S13 File — (PDF) [file pone.0183410.s013.pdf]

Supplemental File 13

<u>Constitutive snoRNA analysis</u>

<u>Smokers</u>

for the manuscript: “snoRNA and piRNA expression levels modified by tobacco use in women with lung adenocarcinoma” by

Natasha Andressa Nogueira Jorge, Gabriel Wajnberg, Carlos Gil Ferreira, Benilton de Sa Carvalho, Fabio Passetti

To identify the snoRNAs or piRNAs which expression levels that do not change between normal and tumor samples we performed a dispersion analysis. We considered as constitutive all the genes which expression is greater than 1 CPM in all samples, none of the samples are 2 times greater or lower than the average log2 expression, and standard deviation is lower than 1. In this analysis, we found 33 constitutive sncRNAs. Table 1 shows the log2 CPM for each evaluated sample, variance, and standard deviation of all genes

Table 1. Constitutively expressed sncRNAs.

Gene	T0N	T0T	T10N	T10T	T12N	T12T	T13N	T13T	T18N	T18T	T1N	T1T	Var	SD
U59B	11.50	11.05	11.56	11.58	11.53	11.03	11.90	11.49	11.74	10.87	11.08	11.23	0.1	0.32
U25	13.24	13.95	12.68	12.93	12.97	12.98	13.14	13.57	13.13	13.52	12.62	12.57	0.17	0.41
U57	12.71	12.29	12.37	12.40	12.28	11.78	12.25	12.91	12.67	11.72	11.69	11.88	0.17	0.41
U48	12.81	13.26	13.23	13.56	13.49	13.46	13.34	12.75	13.38	14.38	13.39	12.89	0.19	0.43
U27	14.04	14.30	13.27	14.65	14.07	13.93	14.05	14.04	13.93	13.40	12.96	13.92	0.21	0.46
U21	11.90	11.95	12.52	11.94	12.28	12.87	11.28	12.62	12.36	12.85	13.21	12.03	0.29	0.53
HBII-420	14.95	14.53	15.03	14.01	14.24	14.78	14.67	14.85	14.49	14.90	16.25	14.67	0.3	0.55
U20	13.15	13.07	13.00	11.70	12.94	12.96	12.70	13.61	12.90	11.75	12.98	12.22	0.33	0.57
U43	13.73	12.91	12.84	12.45	13.74	13.25	13.43	12.72	14.26	13.67	14.02	12.59	0.36	0.6
snR39B	12.67	12.55	12.00	11.33	12.42	13.40	12.90	12.97	12.19	12.85	11.79	13.31	0.38	0.62
U63	12.27	11.63	12.28	12.46	12.18	11.20	12.12	10.85	12.47	11.32	11.23	12.73	0.38	0.62
U104	17.27	16.82	17.30	16.55	16.87	15.92	17.33	15.43	17.25	16.75	17.59	16.24	0.42	0.65
HBII-336	12.08	11.69	10.78	12.76	11.98	10.98	11.22	10.43	12.02	11.32	11.00	11.58	0.43	0.66
HBII-419	14.61	15.15	13.80	13.61	14.70	14.75	14.90	14.43	14.73	14.67	13.85	12.86	0.44	0.66
SNORD119	13.00	12.25	12.70	13.11	12.27	12.45	12.61	13.67	12.57	10.82	12.67	12.45	0.45	0.67
U28	11.59	11.62	12.97	12.13	11.16	12.05	11.34	13.45	12.19	11.44	12.04	11.54	0.46	0.68
U42A	11.87	12.52	11.61	11.70	11.46	12.97	11.71	13.60	11.66	11.92	12.59	11.46	0.46	0.68
U38A	12.04	10.90	12.34	11.37	12.33	11.98	11.92	13.31	12.63	11.57	13.10	11.76	0.48	0.69
U59A	11.21	10.72	11.31	11.46	11.60	11.01	11.36	10.72	10.82	10.01	9.16	11.29	0.49	0.7
HBII-55	12.04	10.85	11.90	10.98	11.21	9.81	12.01	10.66	11.95	10.29	11.00	10.80	0.52	0.72
U31	15.83	15.19	16.15	15.06	15.28	14.09	15.84	14.40	15.77	14.12	14.70	14.35	0.53	0.73
HBII-142	16.20	15.29	15.99	15.33	16.61	15.58	15.88	14.39	16.44	15.58	16.84	14.72	0.55	0.74
ACA45	13.56	12.18	13.04	11.64	12.95	11.67	13.20	12.50	13.76	12.01	13.55	12.17	0.57	0.75
HBII-210	11.85	11.05	12.25	12.32	10.99	11.16	11.67	11.47	11.30	11.24	11.16	13.63	0.58	0.76
U52	11.99	11.79	11.70	12.15	12.12	11.20	12.41	9.72	11.97	12.52	11.00	11.14	0.6	0.78
U95	13.33	13.61	13.60	14.44	13.36	12.87	13.48	12.38	13.14	13.69	13.08	15.54	0.64	0.8
HBII-251	13.07	11.81	12.90	11.38	12.92	11.43	12.97	11.05	13.24	11.63	12.86	11.67	0.65	0.81
HBI-100	11.36	11.42	11.78	10.99	11.48	11.31	11.65	10.07	11.54	12.21	13.55	11.08	0.67	0.82
U60	13.97	13.73	13.95	16.11	13.88	14.92	13.82	15.27	14.71	14.23	13.23	15.33	0.71	0.84
U51	11.62	11.21	12.37	13.56	11.72	11.01	11.92	11.17	11.85	11.49	10.29	13.14	0.82	0.91
U50	10.50	11.42	12.07	11.34	11.18	12.82	10.79	13.54	10.72	10.76	11.08	12.18	0.88	0.94

HBII-295	13.54	12.37	14.96	12.56	13.67	12.31	13.01	12.36	14.02	12.76	14.72	12.39	0.9	0.95
U30	17.33	19.60	17.71	18.32	18.71	19.39	17.98	19.95	18.52	19.45	16.98	17.99	0.9	0.95

Var: Variance. SD: Standard deviation
